# Prefrontal, parietal, and limbic condition-dependent differences in bipolar disorder: a large-scale meta-analysis of functional neuroimaging studies

**DOI:** 10.1038/s41380-023-01974-8

**Published:** 2023-02-13

**Authors:** Maya C. Schumer, Henry W. Chase, Renata Rozovsky, Simon B. Eickhoff, Mary L. Phillips

**Affiliations:** 1 Department of Psychiatry, University of Pittsburgh School of Medicine, Pittsburgh, PA USA; 2 Institute of Systems Neuroscience, Medical Faculty, Heinrich Heine University Düsseldorf, Düsseldorf, Germany; 3 Institute of Neuroscience and Medicine, Brain & Behaviour (INM-7), Research Centre Jülich, Jülich, Germany

**Keywords:** Biomarkers, Neuroscience

## Abstract

**Background:**

Over the past few decades, neuroimaging research in Bipolar Disorder (BD) has identified neural differences underlying cognitive and emotional processing. However, substantial clinical and methodological heterogeneity present across neuroimaging experiments potentially hinders the identification of consistent neural biomarkers of BD. This meta-analysis aims to comprehensively reassess brain activation and connectivity in BD in order to identify replicable differences that converge across and within resting-state, cognitive, and emotional neuroimaging experiments.

**Methods:**

Neuroimaging experiments (using fMRI, PET, or arterial spin labeling) reporting whole-brain results in adults with BD and controls published from December 1999—June 18, 2019 were identified via PubMed search. Coordinates showing significant activation and/or connectivity differences between BD participants and controls during resting-state, emotional, or cognitive tasks were extracted. Four parallel, independent meta-analyses were calculated using the revised activation likelihood estimation algorithm: all experiment types, all resting-state experiments, all cognitive experiments, and all emotional experiments. To confirm reliability of identified clusters, two different meta-analytic significance tests were employed.

**Results:**

205 published studies yielding 506 individual neuroimaging experiments (150 resting-state, 134 cognitive, 222 emotional) comprising 5745 BD and 8023 control participants were included. Five regions survived both significance tests. Individuals with BD showed functional differences in the right posterior cingulate cortex during resting-state experiments, the left amygdala during emotional experiments, including those using a mixed (positive/negative) valence manipulation, and the left superior and right inferior parietal lobules during cognitive experiments, while hyperactivating the left medial orbitofrontal cortex during cognitive experiments. Across all experiments, there was convergence in the right caudate extending to the ventral striatum, surviving only one significance test.

**Conclusions:**

Our findings indicate reproducible localization of prefrontal, parietal, and limbic differences distinguishing BD from control participants that are condition-dependent, despite heterogeneity, and point towards a framework for identifying reproducible differences in BD that may guide diagnosis and treatment.

## Introduction

Bipolar Disorder (BD) is a common, debilitating psychiatric disorder resulting in disease burden worldwide [1]. The past several decades of neuroimaging research have investigated the neural substrates of mechanisms underlying differences in cognitive and emotional processing that are characteristic of BD [2, 3] which has enabled the conceptualization of neural models [2, 4] that are critical to understanding it. The study of neural differences in BD associated with both brain activation (i.e., regional BOLD signaling) and functional connectivity (i.e., the correlation between different brain regions that can elucidate the nature of neural network dynamics) [5] enables the identification of biomarkers that improve diagnostic precision, facilitate early identification, and inform targets for treatment developments [6]. However, the presence of clinical heterogeneity [7, 8] (e.g., differences in healthcare systems [9, 10], diagnostic subtypes [11, 12], mood state [13, 14], treatment response [7, 15], comorbidity [7], chronicity, severity [16]), methodological differences (imaging modality, paradigm), and analytical flexibility [17, 18], as well as the impact of physiological noise sources [19–21] and variability of neural responses to cognitive manipulations [22–25] may all hinder the identification of consistent neural biomarkers of Bipolar-related illness [7, 12, 26, 27].

While high-powered structural studies [28] and qualitative reviews [2, 4] are informative to the development of theoretical models of BD, coordinate-based meta-analysis techniques, such as activation likelihood estimation (ALE) [29, 30], can test meta-analytic hypotheses at the level of the whole brain in a spatially unbiased fashion [31], taking into account hundreds to thousands of participants and disparities in experimental design decisions [29]. Moreover, depending on how the hypothesis is constructed, successful refutation of the null hypothesis can provide preliminary evidence for potential reproducible differences distinguishing individuals with BD from control participants [32]. However, the extent to which this is possible depends on the quality and number of studies included.

It is widely known that functional neuroimaging studies are hampered by great heterogeneity and low power due to small sample sizes, leading to the use of lower, often uncorrected, thresholds to obtain positive results, and thus a substantial risk of frequent false positive findings [33]. It is thus important to acknowledge that the neuroimaging literature on BD is likely to include numerous under-powered studies using phenotypically heterogenous samples and disproportionately characterized by positive exploratory findings rather than evaluation of the magnitude of a priori hypothesized effects [4]. Nevertheless, meta-analyses are needed to reconcile the literature’s pitfalls and provide a framework to test whether the findings of small, heterogenous studies can be reproducible across different studies [31]. Meta-analyses can be used to synthesize results of individual studies in spite of heterogeneity, thereby allowing readers to draw wider conclusions about the state of the literature at large (including whether any reported effects are reproducible). They also highlight irregularities and issues present in the field which, importantly, provides transparency that can guide future study designs and encourage replications [31]. Given the rapid rate at which neuroimaging studies of BD are being conducted and published, meta-analyses are useful in that they comprehensively, quantitatively summarize and integrate disparate findings, building cumulative knowledge and guiding future work [33]. ALE is also statistically conservative [34], using cluster-level family-wise error correction which leads to a low likelihood of false positive convergence, especially if a significant region includes contributing foci from several studies rather than a disproportionate contribution from a single study [31, 35]. Individual studies reporting results at uncorrected thresholds may be used with ALE, given that uncorrected thresholds can provide a favorable balance between false positives and false negatives [36].

Previous coordinate-based meta-analyses have found correlates of BD across emotion-processing experiments distinguishing BD from both non-clinical [37] and clinical controls, such as unipolar depression [38] and schizophrenia [39], across resting-state experiments [40, 41], and across both cognitive and emotional experiments [42]. However, these meta-analyses had a narrower focus and were limited by the available data which often had smaller sample sizes. Additionally, they did not incorporate techniques that have been used more recently in psychiatric neuroimaging research (e.g., Amplitude of Low Frequency Fluctuations (ALFF) [43, 44], Independent Component Analysis (ICA) [45], Regional Homogeneity (ReHo) [46], degree centrality (DC) [47], functional connectivity strength (FCS) [47]). Furthermore, despite there being extensive neurocognitive differences in BD [48–51], there are no ALE meta-analyses of BD solely examining cognitive experiments.

Notwithstanding these gaps and advancements, no meta-analyses have examined the effect of condition (i.e., changes in neural activity and connectivity in response to changing task requirements and/or the level of arousal) via testing for a potential invariant condition-independent, or condition-dependent (i.e., clusters that converge across experiments or paradigms of one type, but not across experiments of a different type) functional marker of BD. Given the extensive evidence showing functional and structural differences in limbic regions, particularly the amygdala [4], across different mood states and neuroimaging modalities (e.g., structural magnetic resonance imaging (MRI), diffusion tensor imaging, resting-state, emotional and cognitive paradigms) [4], there are empirical grounds for suggesting the existence of a condition-independent marker of BD. Such a marker could manifest across a variety of cognitive (e.g., working memory), and emotional paradigms, and contribute to the differences in these processes distinguishing BD [51–57]. Alternatively, functional neural differences in BD may be more selective, with distinct markers being observed across different paradigm types.

Thus, the objective of this investigation was to comprehensively reassess brain activation and functional connectivity in BD in order to identify a reproducible, condition-independent neural correlate of BD that converges across resting-state, cognitive, and emotional experiments combined. To our knowledge, this study is the largest, high-powered, most comprehensive meta-analysis of BD functional neuroimaging experiments to date, which is necessary to justify whether the current state of the literature allows for identification of replicable differences in BD despite significant heterogeneity and the mixed reliability of task-based functional MRI (fMRI) for brain biomarker discovery [58, 59]. An omnibus meta-analysis across all experiment types tested the hypothesis that there would be a condition-independent neural marker differentiating BD from controls that can be seen regardless of task type, akin to a simple localized deficit which is generalizable across paradigms and modalities [28]. Parallel independent, individual meta-analyses of resting-state, cognitive, and emotional processing experiments each tested for condition-dependent neural signatures of BD. Meta-analysis across resting-state experiments tested for a potential core functional difference [2] that is reliable [43, 60] and relatively unconfounded by task effects compared to task-based fMRI [61]. Across cognitive tasks using non-affective stimuli, we hypothesized that there would be a functional difference related to cognition given that individuals with BD have impaired executive functioning, sustained attention, working and verbal memory [3, 48, 50, 51, 57], and this cognitive signature would significantly differ from both the null distribution as well as resting-state and emotional experiments. We further tested whether any significant cognitive differences were hyper/hypoactive in participants with BD. Furthermore, we hypothesized that there would be an emotion-related difference specific to emotion processing-related paradigms, given behavioral differences associated with mood lability and emotion dysregulation in BD [2, 52, 53]. We also tested whether this signature was specific to valence via post-hoc subgroup meta-analyses of experiments associated with negative, positive and mixed (negative and positive) valence, and whether any significant emotion-processing differences were hyper/hypoactive in participants with BD. Finally, we examined the contribution of clinical confounds and nesting (i.e., a single study contributing more than one experiment or contrast to a cluster) using two different meta-analytic significance tests to confirm the reliability of meta-analytic findings.

## Methods

### Search and selection

**Fig. 1:**
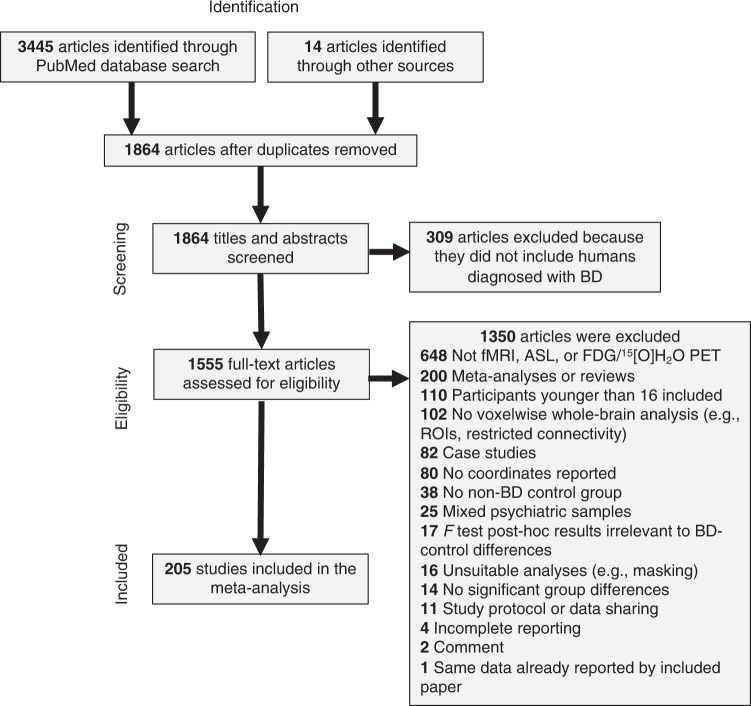
Flowchart of study selection. ASL Arterial Spin Labeling, BD Bipolar Disorder, FDG Fluorodeoxyglucose, fMRI Functional Magnetic Resonance Imaging, PET Positron Emission Tomography, ROI Region Of Interest.

Figure 1 depicts the study selection process and reasons for exclusion. Details on eligibility criteria and literature search terms can be found in the Supplementary Methods. In brief, resting-state and task-based (cognitive and emotional) functional neuroimaging experiments using fMRI, positron emission tomography (PET), or arterial spin labeling (ASL) published online from December 1, 1999 through July 18, 2019 were identified from a systematic PubMed search. To be eligible for inclusion, experiments had to report voxelwise whole-brain results via standard whole-brain analyses, seed-to-voxel functional connectivity (including psychophysiological interactions (PPIs), granger causality mapping (GCM), and beta-series correlation for task-based experiments), ICA, ReHo, ALFF/fractional ALFF (fALFF), voxel-mirrored homotopic connectivity (VMHC), FCS, DC, eigenvector centrality mapping (ECM), in standard stereotaxic space (Montreal Neuroimaging Institute (MNI) or Talairach) that statistically compared adults (≥16 years old) diagnosed with BD to an adult non-BD control group (non-clinical and/or clinical). The minimum age was 16 and the mean age was over 18. While the majority of included studies measured adults aged 18 or older, a minority of studies included participants aged 16. These studies were included so as to increase the number of relevant studies in the meta-analysis and be as inclusive as possible. Pediatric (<16 years of age) and at-risk cases of BD were excluded to mitigate variability in neural activations that might be secondary to developing sex hormone effects [62–66].

Cognitive experiments were operationalized as tasks using a cognitive paradigm with non-affective stimuli, and contrasts comparing a cognitive challenge to either a less-challenging control (e.g., 3-back vs. 1-back) or a baseline condition (e.g., 0-back, rest) were both included.

Emotional experiments were operationalized as tasks presenting an emotional visual, auditory, or sensory (e.g., pain, odor) stimulus or invoking an emotion (e.g., sad mood induction). Contrasts comparing an emotional condition to either a non-emotional/neutral condition, resting/baseline condition, or other emotional condition were all included. Compound emotional/cognitive tasks, operationalized as cognitive paradigms with an emotional manipulation (e.g., go/no-go with emotional distractors), were also included. Emotional tasks were then further separated into valence classes for post-hoc meta-analyses: Negative valence was operationalized as stimuli representing or invoking fear, sadness, anger, disgust, pain, loss, or punishment*;* positive valence was operationalized as stimuli representing or invoking happiness, pleasure, or rewards*;* mixed valence was operationalized as positive and negative valence stimuli/conditions collapsed (e.g., all emotional faces vs. baseline); neutral was defined as a non-emotional condition or stimulus (e.g., blank face, shape).

### Data extraction and experimental design

Information was extracted from each experiment on (a) sample size, (b) imaging technique (fMRI, PET, or ASL), (c) task type (resting-state, cognitive, or emotional), (d) directionality (i.e., group differences reported by *t*-tests, or nondirectional group effects and group-by-condition interactions reported by *F* statistics), (e) peak MNI or Talairach coordinates, (f) level of arousal (emotional conditions were arousing; cognitive and non-emotional/neutral conditions were non-arousing), and (g) valence. Additional experiment characteristics such as BD mood state (hypo/manic or mixed (collapsed due to low power i.e., there were fewer than 17 mixed state experiments [35]), depressed, euthymic/remitted, or combined/not reported), current presence and/or history of psychosis, medication status, BD diagnostic subtype (I, II, Not Otherwise Specified), control group type (non-clinical and/or clinical), and BD participants’ age and gender were also extracted. Further details on data extraction are provided in the Supplementary Methods.

### Activation likelihood estimation

Meta-analyses were performed using the revised ALE algorithm [30] (detailed description in the Supplementary Methods). All ALE results are reported at *p* < .05 family-wise error (FWE) cluster-level corrected (cluster-forming threshold at voxel-level *p* < .001) in MNI space, consistent with previous ALE meta-analyses. The SPM Anatomy Toolbox [67] was used to obtain MNI coordinates and *z* statistics. In addition to the location of significant clusters, the ALE software indicates the experiments which contributed to a given cluster. This contribution information was used for post-hoc analyses (see ‘assessments of robustness and post-hoc analyses’ section).

### Planned meta-analyses

**Fig. 2:**
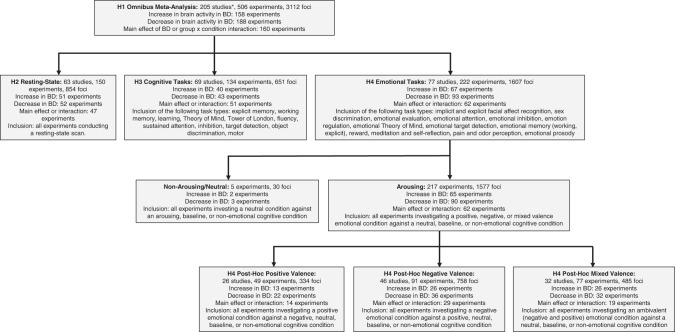
Description of meta-analyses. BD Bipolar Disorder, H Hypothesis. *One study contributed both resting-state and cognitive experiments; two studies contributed both cognitive and emotional experiments. The number of foci for each meta-analysis reflects the total number of foci when all individual (nested) experiments were included.

In total, 4 primary independent meta-analyses were performed: omnibus, resting-state, cognition, and emotion (Fig. 2; Supplementary Tables 1–4). Meta-analyses were calculated across reported patterns of BD hyper/hypoactivation (see previous ALE meta-analyses [68–70]), which accommodates flexibility in how research groups calculated group differences, i.e., some task-based experiments contrast a task with either a less-difficult control condition (e.g., 3-back >1-back) or a less-tightly controlled comparison condition (e.g., crosshair) on the subject-level, and group-level comparisons are subsequently calculated; whereas other experiments report group-by-condition interactions calculated on the second-level. In this way, the direction of the BD vs. control difference (i.e., hyper/hypoactivation) can depend on the type of control condition, and given that control conditions vary widely across experiments, different calculation approaches may influence the direction of group differences. Thus, this pooled analysis allowing for the discovery of converging activation differentiating BD from controls provides the most comprehensive summary of neuroimaging findings in BD. Additionally, meta-analyses were pooled across mood states and control groups due to asymmetric power i.e., the vast majority of experiments either examined euthymia, depression, or combined mood states, with a small number of hypo/manic and mixed state experiments (these latter two states were often collapsed within a sample), and most experiments compared participants with BD to non-clinical controls (74%). Although there were experiments with clinical controls (26%), half of those comparisons used *F* statistics generated across non-clinical, clinical, and BD groups, while the other half compared participants with BD directly to clinical controls.

The omnibus meta-analysis, collapsed across all experiments, tested for a condition-independent difference in BD i.e., a concordant signature that could be seen across all task types. To confirm that the omnibus meta-analysis represented a distinct, independent hypothesis from the other three, we added a secondary stipulation for this hypothesis, namely that any regions identified must not show a significant bias for one paradigm type over another i.e. that all paradigm types (emotion, cognition, rest) should contribute to the cluster in an approximately similar fashion. To test this hypothesis, we conducted Bayesian *χ*^2^ tests assuming a Poisson sampling plan, which can provide evidence for and against the null hypothesis of no difference between contributing paradigm types [71]. Exploratory meta-analyses of task-based activation experiments are reported in the Supplementary Results and Supplementary Table 6.

The resting-state meta-analysis tested for convergence across resting-state experiments followed by separate post-hoc contrasts against cognitive and emotional experiments testing whether resting-state signatures were significantly different from task-based experiments to provide further evidence for the specificity of group differences for cognitive and emotional manipulations. Post-hoc meta-analyses tested whether there were dissociations in these signatures across mood states. Exploratory meta-analyses of seed-to-voxel resting-state functional connectivity experiments are reported in the Supplementary Results and Supplementary Table 6. We did not perform planned tests of additional meta-analytic connectivity differences specific to other resting-state methods if there were fewer than 17 studies [35]. We did not test for directionality effects in BD due to the variety of different seeds and methods being used.

The cognition meta-analysis tested for a neural signature associated with cognitive task differences in BD that would significantly converge across cognitive experiments and further tested for their specificity to cognitive manipulations only via separate post-hoc contrasts against resting-state and emotional experiments. Post-hoc meta-analyses tested whether these signatures were hyper/hypoactive in participants with BD compared to controls, and whether there were dissociations in these signatures across mood states. Exploratory meta-analyses of working memory paradigms are reported in the Supplementary Results and Supplementary Table 6. We were did not perform planned meta-analyses of additional cognitive domains if there were fewer than 17 studies [35]).

The emotion meta-analysis, collapsed across all emotional tasks, tested for an emotion-specific neural signature of BD, followed by post-hoc subgroup meta-analyses of valence (negative, positive, mixed) that examined the locus of the difference (Supplementary Table 5), and whether any significant effects were significantly different from cognitive and resting-state experiments via separate post-hoc contrasts to further establish that emotional task signatures are unique to emotional manipulations. Additional post-hoc meta-analyses tested for emotional task signatures that were hyper/hypoactive in participants with BD compared to controls as well as whether there were dissociations in these clusters across mood states. Exploratory meta-analyses of emotional reactivity and emotion regulation paradigms are reported in the Supplementary Results and Supplementary Table 6.

### Assessments of robustness and post-hoc analyses

Converging clusters were evaluated for reliability using two different meta-analytic methods. The first, primary method pooled together all coordinates from all contrasts (if more than one was included) from a given study into one experiment [70], such that each study only contributed one experimental contrast to the ALE, thereby enabling the modeling of random effects [72]. To assess the robustness of meta-analytic findings yielded by this initial inference with a different set of assumptions, we also conducted a two-part robustness test. The first step treated each individual contrast as a separate experiment to examine the contribution of different experiment characteristics (e.g., studies that included more than one mood state contrast); this initial approach intentionally allowed for within-study clustering, or nesting, of effects. The second step examined the impact of nesting on meta-analytic findings yielded by the first step: for studies contributing more than one contrast to a significant cluster (i.e., nested studies), we re-ran the ALE keeping the least contributing experiment to the cluster (i.e., having the lowest ALE contribution score; detailed description in the Supplementary Methods) and removing the other experiments belonging to the same study, both the ones that more strongly contributed to the cluster and the ones that did not contribute, thus keeping only one contrast per nested study. This analysis was performed in order to reduce within-study bias that nesting can introduce, which is not accounted for by the ALE algorithm. Clusters that reached significance both when coordinates were initially pooled and then after nested experiments were removed were considered to be significant effects. For each meta-analysis, we focus on findings that survived both the pooled test and the subsequent robustness assessment.

We also examined the contribution of overlapping study samples (i.e., multiple studies whose samples came from the same research group/laboratory) post-hoc for findings passing both significance tests; this information can be found in the Supplementary Results.

Details of the experiments that contributed to each cluster from the pooled, nested, post-hoc, and exploratory meta-analyses are provided in Supplementary Tables 7–32.

Post-hoc analyses of the experiment contribution information were conducted to assess the impact of directionality, mood state, BD diagnostic subtype, medication status, psychosis, age, gender, and control group type so as to examine whether certain experiment types were over/underrepresented in the findings; these analyses were performed to evaluate whether these clinical heterogeneity factors confounded the significant findings of interest [12, 73, 74]. These analyses were conducted using the contribution information from the nested analysis approach that allows for the examination of different experiment characteristics. We conducted two-tailed Fisher’s Exact Tests of independence in SPSS Version 27, in which one variable designated whether a given experiment was contributing or not contributing to a significant cluster and the other represented the observed frequencies for each sub-category of the above factors. Results were FDR-corrected using the Benjamini-Hochberg procedure [75], although uncorrected *p* values are reported alongside FDR-corrected *q* values. Results of all post-hoc analyses are reported in the Supplementary Results, while a summary is provided in the main text.

In addition, we sought to use the contribution information to provide an estimate of the underlying effect size. Effect size estimation in fMRI can be difficult as the statistic representing a cluster’s peak provides a biased (inflated) estimate of effect size [76]. Instead, we used the (observed) proportion of experiments contributing versus not contributing to a cluster for the pooled method, and, using a Fisher’s Exact Test, compared it to a null distribution in which the (expected) rate of contribution was equivalent to the expected false positive rate given an effect size of zero (i.e. alpha). We used *p* < 0.01 uncorrected as an estimate of alpha, given that we included coordinate maps obtained using uncorrected thresholds. The resulting *p* value from the Fisher’s Exact test was converted into a *χ*^2^ statistic via an inverse *χ*^2^ distribution, which was converted into a Pearson’s *r* statistic [77] and then an effect size estimate (Cohen’s *d* [78]).

## Results

205 published studies with 506 individual neuroimaging experiments (yielding 3112 foci total) published from 1999–2019 met the criteria for inclusion in this meta-analysis. Demographic, clinical, and methodological details and citations of included papers are provided in Supplementary Table 33. The total sample covering 13768 participants comprised 5745 individuals with BD, 5919 non-clinical controls, and 2104 clinical controls (Tables 1 and 2).

**Table 1:** Significant clusters and contrasts from a priori ALE meta-analyses.

Task type or contrast	*N* foci	*N* BD	*N* controls	Peak coordinates (MNI)	Location	Cluster Size (*k* voxels)	Peak Intensity (*Z)*
All task types	2910	5745	8023	10, 10, −8 *14, 12, 14* *12, 14, 10* *10, 12, −2*	Anterior Caudate ext. to Ventral Striatum^a^	157	4.44 *3.94* *3.86* *3.42*
Resting-state	786	2218	3093	6, −52, 32	Posterior Cingulate Cortex^b^	144	4.47
Cognitive tasks	619	1848	3034	38, −44, 46 *36, −56, 48* −20, −70, 52 −2, 52, −14 −10, 8, −2 *−16, 6, −18* *−2, 12, −4*	Inferior Parietal Lobule^b^ Superior Parietal Lobule^b^ Medial Orbitofrontal Cortex^a^ Anterior Caudate ext. to Ventral Striatum, Subgenual Anterior Cingulate Cortex^a^	154 121 115 144	5.21 *4.97* 5.87 5.27 4.53 *4.21* *3.94*
Emotional tasks	1505	1762	2028	−26, −6, −20 *−20, −14, −14*	Amygdala ext. to Hippocampus^b^	128	4.25 *3.47*
Resting-state > cognitive	1405	4066	6127	4, −50, 28	Posterior Cingulate Cortex^c^	143	3.43
Cognitive > resting-state	1405	4066	6127	42, −46, 50 *38, −54, 52* −24, −70, 46 2, 50, −12 −6, 10, −2 −18, 4, −16	Inferior Parietal Lobule^c^ Superior Parietal Lobule^c^ Medial Orbitofrontal Cortex Ventral Striatum ext. to Subgenual Anterior Cingulate Cortex Posterior Central Orbitofrontal Cortex	119 83 82 92 10	2.91 *2.18* 2.51 2.64 2.92 1.94
Resting-state > emotional	2291	3980	5121	2, −54, 36	Posterior Cingulate Cortex^c^	93	3.29
Emotional > resting-state	2291	3980	5121	−20, −6, −18	Amygdala^c^	108	3.34
Cognitive > emotional	2124	3610	5062	40, −46, 44 −18, −70, 50 0, 54, −10 −14, 8, −14	Inferior Parietal Lobule^c^ Superior Parietal Lobule^c^ Medial Orbitofrontal Cortex Subgenual Anterior Cingulate Cortex	101 93 9 12	2.32 2.57 1.86 2.02
Emotional > cognitive	2124	3610	5062	−26, −6, −20 *−20, −12, −14*	Amygdala ext. to Hippocampus^c^	128	4.25 *3.47*

To avoid double counting, sample sizes (N) correspond to the total number of study participants, not the number of individual experiment participants.

*BD* Bipolar Disorder, *C* Controls, *ext.* Extended, *MNI* Montreal Neuroimaging Institute.

^a^Seen when experiments were pooled.

^b^Seen both when nested experiments were removed and when experiments were pooled.

^c^Clusters passing both significance tests that were also observed in contrasts.

**Table 2:** Significant clusters and contrasts from post-hoc ALE meta-analyses.

Task type, contrast, or interaction	*N* foci	*N* BD	*N* controls	Peak coordinates (MNI)	Location	Cluster size (*k* voxels)	Peak intensity (*Z)*
Cognitive tasks (BD > C)	118	660	781	−4, 52, −16 −4, 36, −14 *0, 38, 4* *−10, 46, −6*	Medial Orbitofrontal Cortex^b^ Ventral Anterior Cingulate Cortex^a,c^	156 220	5.56 4.6 *4.17* *3.42*
Cognitive tasks (C > BD)	250	816	963	−32, −2, 52	Premotor/Supplementary Motor Cortex^a,c^	121	5.47
Emotional tasks (BD > C)	451	621	824	–	*No Convergence*	–	–
Emotional tasks (C > BD)	690	709	896	32, 30, −14	Ventrolateral Prefrontal Cortex^a,c^	125	5.46
Positive valence	334	623	739	–	*No Convergence*	–	–
Negative valence	706	1127	1220	–	*No Convergence*	–	–
Mixed valence	436	723	988	−28, −8, −24 *−30, −2, −28* *−22, −12, −14*	Amygdala ext. to Hippocampus^b^	196	3.77 *3.66* *3.38*
Euthymic BD & Resting-state	230	526	561	–	*No Convergence*	–	–
Depressed BD & Resting-state	372	1107	1694	6, −52, 32 *−6, −54, 30*	Posterior Cingulate Cortex^b^	157	4.62 *3.51*
Hypo/manic BD & Resting-state	28	52	103	–	*No Convergence*	–	–
Euthymic BD & Cognitive tasks	288	738	1170	−20, −70, 52	Superior Parietal Lobule^b^	121	6.21
Depressed BD & Cognitive tasks	220	329	481	–	*No Convergence*	–	–
Hypo/manic BD & Cognitive tasks	27	163	198	−32, −2, 58	Premotor/Supplementary Motor Cortex^a,c^	118	5.62
Euthymic BD & Emotional tasks	663	828	945	10, 10, −6 *10, 16, 10*	Anterior Caudate ext. to Ventral Striatum^a^	131	4.5 *3.65*
Depressed BD & Emotional tasks	546	321	504	–	*No Convergence*	–	–
Hypo/manic BD & Emotional tasks	110	123	135	–	*No Convergence*	–	–

To avoid double counting, sample sizes (*N*) correspond to the total number of study participants, not the number of individual experiment participants. *No Convergence* indicates a null meta-analytic result i.e., no significant clusters were identified.

*BD* Bipolar Disorder, *C* Controls, *ext.* Extended, *MNI* Montreal Neuroimaging Institute.

^a^Seen when experiments were pooled.

^b^Seen both when nested experiments were removed and when experiments were pooled.

^c^Novel clusters which emerged in the post-hoc meta-analyses and were not observed in the a priori meta-analyses.

### Meta-analyses across all experiments

**Fig. 3:**
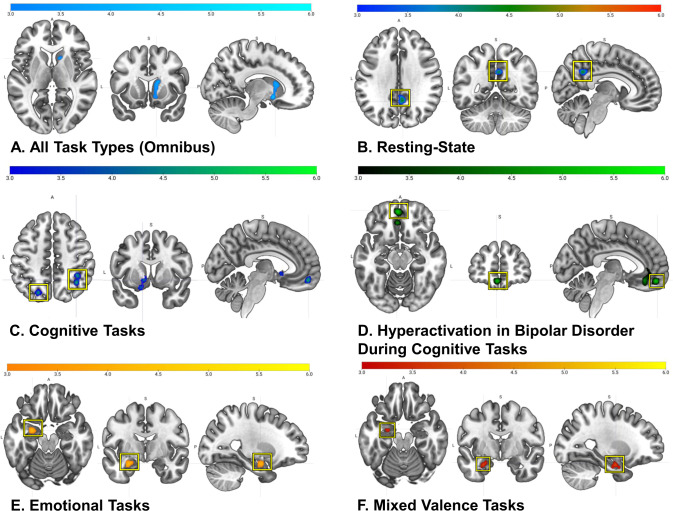
Overview of all significant clusters (pFWE < .05) from the a priori ALE meta-analyses. Meta-analyses with clusters highlighted in yellow boxes indicate that these clusters survived both significance tests, whereas non-highlighted clusters only survived one significance test (i.e., the pooled analysis). Activations displayed in the sagittal slices reflect the position in the y/z axis.

There were no significant findings from the omnibus meta-analysis that survived both the initial pooled test and the subsequent nested robustness assessment. Across all experiments (number of included experiments (NIE) = 205 in the pooled meta-analysis; NIE with nesting = 506), there was convergence in the right anterior caudate extending to the ventral striatum that survived the pooled test, but not the nested test, yielding an estimated effect size *d* = 0.52 (Fig. 3A). Of the 28 experiments that contributed to the striatal cluster in the pooled analysis, 25% were resting-state experiments (primarily employing seed-to-voxel functional connectivity) and 75% were task-based (primarily activation), of which 38% were cognitive paradigms and 62% were emotional. To confirm the independence of these observations across paradigm types, a Bayesian *χ*^2^ test revealed evidence in favor of the null hypothesis of no differences between contributing paradigms in the striatum (Bayes Factor (BF) = 0.18 with an inverse of 5.61, evidence for the null hypothesis).

### Meta-analyses across resting-state experiments

**Fig. 4:**
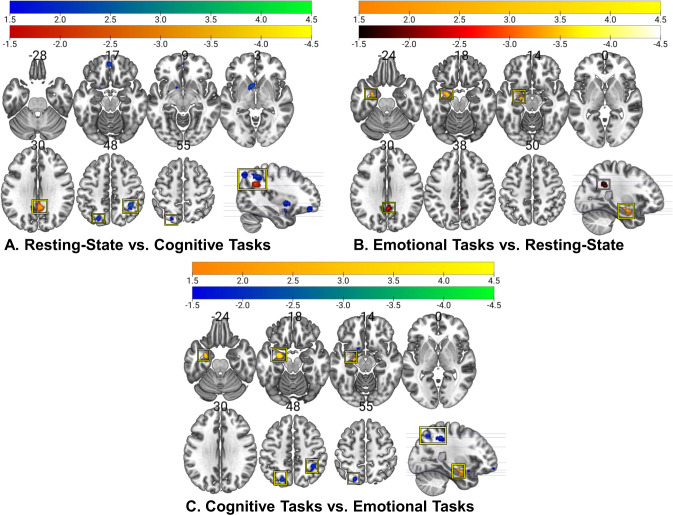
Contrast maps of activation distinguishing BD from controls (pFWE < .05) for specific task types. Activations displayed in the sagittal slices reflect the position in the y/z axis. **A** Resting**-**state > cognitive clusters are shown in orange (slice 30) and red (sagittal slice); cognitive > resting-state clusters are shown in blue. **B** Resting-state > emotional clusters are shown in red (slice 30) and burgundy (sagittal slice); emotional > resting-state clusters are shown in yellow. **C** Emotional > cognitive clusters are shown in yellow; cognitive > emotional clusters are shown in blue.

Across resting-state experiments (pooled NIE = 63; nested NIE = 150), there was significant convergence in the right ventral posterior cingulate cortex (PCC), which survived both significance tests and yielded an estimated effect size *d* = 0.53 (Fig. 3B). The PCC significantly differed from both cognitive experiments (Fig. 4A) and emotional experiments (Fig. 4B), demonstrating that connectivity in this region distinguishing BD from controls is unique to resting-state experiments.

Post-hoc tests evaluating the effect of mood state on resting-state differences revealed preliminary evidence for dissociations across mood states: the meta-analysis of resting-state experiments in depressed BD participants (pooled NIE = 29; nested NIE = 62) revealed significant convergence in the right ventral PCC that survived both significance tests (Supplementary Fig. 1); but meta-analyses of resting-state experiments in euthymic (pooled NIE = 18; nested NIE = 45) and hypo/manic BD participants (pooled NIE = 3; nested NIE = 4) did not reveal significant results.

### Meta-analyses across cognitive experiments

Meta-analysis across cognitive experiments (pooled NIE = 69; nested NIE = 134) yielded four significant clusters of convergence: the right inferior parietal lobule (IPL) extending to the right angular gyrus (estimated effect size *d* = 0.41) and the left superior parietal lobule (SPL) (estimated effect size *d* = 0.41), both of which survived both significance tests, the left medial orbitofrontal cortex (mOFC), which survived the pooled test, but not the nested test (estimated effect size *d* = 0.41) and the left anterior caudate extending to the ventral striatum and subgenual anterior cingulate cortex, which was observed in the pooled analysis only (estimated effect size *d* = 0.54) (Fig. 3C). All four clusters significantly differed from resting-state experiments (Fig. 4A) and from emotional experiments (Fig. 4C), indicating that these signatures are specific to cognitive experiments.

A post-hoc test examining BD hyperactivation experiments alone (pooled NIE = 27; nested NIE = 40) revealed significant convergence in the left mOFC, which survived both significance tests (estimated effect size *d* = 0.65), as well as in one new cluster (i.e., clusters not initially observed in the a priori cognitive tasks meta-analysis): the left ventral anterior cingulate cortex, observed only when experiments were pooled (*d* = 0.84) (Fig. 3D). The meta-analysis of BD hypoactivation experiments alone (pooled NIE = 29; nested NIE = 43) revealed significant convergence in a new cluster, the left premotor/supplementary motor cortex, which survived the pooled test, but not the nested test (estimated effect size *d* = 0.43) (Supplementary Fig. 2).

Post-hoc tests evaluating the effect of mood state on cognitive task activations revealed preliminary evidence for dissociations across mood states, such that the post-hoc meta-analysis across cognitive tasks in euthymic BD participants (pooled NIE = 31; nested NIE = 55) revealed significant convergence in the left SPL, which survived both significance tests (Supplementary Fig. 3). The meta-analysis of cognitive tasks in depressed BD participants (pooled NIE = 16; nested NIE = 41) did not reveal significant results. Despite being underpowered to test the interaction between hypo/manic BD participants and cognitive tasks (pooled NIE = 7; nested NIE = 12), there was still significant convergence, albeit in a new cluster: the left premotor/supplementary motor cortex, which survived the pooled test only, as there was no nesting observed (Supplementary Fig. 4).

### Meta-analyses across emotional experiments

Meta-analysis of emotional experiments (pooled NIE = 77; nested NIE = 222), pooled across valence, showed significant convergence in the left amygdala extending to the left hippocampus, which survived both significance tests (estimated effect size *d* = 0.75) (Fig. 3E). The amygdala significantly differed from resting-state experiments (Fig. 4B) and from cognitive experiments (Fig. 4C) demonstrating its specificity to emotional processing in BD.

There were no significant findings from the meta-analyses of BD hyper- and hypoactivation emotional experiments alone that survived both significance tests. Meta-analysis of BD hypoactivation experiments alone (pooled NIE = 36; nested NIE = 93) revealed significant convergence in one new cluster not previously observed in the emotional tasks meta-analysis: the right ventrolateral prefrontal cortex (VLPFC), which survived the pooled test, but not the nested test (estimated effect size *d* = 0.85) (Supplementary Fig. 5). Meta-analysis of BD hyperactivation experiments alone (pooled NIE = 32; nested NIE = 67) did not reveal significant results.

The post-hoc meta-analysis across mixed valence experiments (pooled NIE = 32; nested NIE = 76) showed significant convergence in the left basolateral amygdala extending to the left hippocampus (Fig. 3F); this finding survived both significance tests (estimated effect size *d* = 1.00). Meta-analyses across negative valence experiments (pooled NIE = 46; nested NIE = 91) and positive valence experiments (pooled NIE = 26; nested NIE = 49) did not reveal significant results.

The meta-analysis across emotional tasks in euthymic BD participants (pooled NIE = 39, nested NIE = 101) revealed significant convergence in the right anterior caudate extending to the ventral striatum, surviving the pooled test (Supplementary Fig. 6). Post-hoc meta-analyses across emotional tasks in depressed (pooled NIE = 14; nested NIE = 44) and hypo/manic BD participants (pooled NIE = 8; NIE = 23) did not reveal significant results.

### Summary of post-hoc fisher’s exact tests of independence

None of the post-hoc Fisher’s Exact Tests of potential confounds were significant at FDR-corrected thresholds for any of the primary or post-hoc meta-analytic findings passing both significance tests. Further details are provided in the Supplemental Results.

## Discussion

The present study yielded several major findings that survived both significance tests: (1) focused meta-analyses of resting-state and cognitive tasks revealed differences distinguishing BD from controls in connectivity of the right PCC specific to resting-state experiments and in activation of the right IPL and left SPL specific to cognitive experiments; (2) there was hyperactivation in BD of the left mOFC across cognitive tasks; and (3) meta-analysis of emotional tasks revealed differences in activation of the left amygdala specific to emotional experiments, including mixed valence manipulations. The omnibus meta-analysis across all experiments revealed differences in activation of the right striatum, surviving one significance test. The absence of findings surviving both significance tests from the omnibus meta-analysis likely reflects the fact that neural differences in BD are context-specific rather than generalizable across different tasks, as we had sufficient power to detect the latter if present. These findings thus collectively suggest that the extant literature provides support for reproducible localization of context-dependent differences in BD despite significant heterogeneity.

Resting-state experiments revealed differences in connectivity of the PCC, a core default mode network (DMN) node implicated in mood disorders [79, 80], consistent with prior evidence [81, 82]. Altered functional coupling and engagement of the PCC in BD may reflect rumination and/or attention dysregulation at rest and potentially during task performance [83–86]. Across cognitive experiments, there were differences in activation of the IPL and SPL, hubs of the frontoparietal network [87, 88]; contributing experiments covered executive control domains such as response inhibition, working memory and sustained/selective attention, suggesting a possible locus of sustained attentional deficits specific to BD [49, 89–91]. Additionally, the mOFC was hyperactivated in BD primarily during working memory and sustained attention tasks. OFC hyperactivation in non-emotional contexts has been proposed to reflect emotional processing interference due to a heightened salience to emotional perception in BD [2] and has also been found across psychiatric disorders during working memory tasks [92], indicating this finding may be transdiagnostic. Differences in amygdala activation observed across all mood states during emotional and mixed valence experiments is consistent with previous BD consensus models showing state- and direction-independent, emotionally-sensitive amygdala dysfunction [4].

While there was indeed convergence across all experiment types in the right striatum (consistent with previous models and reviews [2, 4, 93]) which survived the pooled test, this finding did not survive the nested test, thus we cannot draw definitive, confident conclusions about whether convergence in the striatum is indicative of an invariant, condition-independent difference in BD because this finding was biased by nesting and thus overly dependent on several experiments. Nonetheless, the null Bayesian *χ*^2^ result shows that this region’s activation appears largely independent from the aforementioned condition-dependent differences. The omnibus results are also encouraging in regard to clinical relevance, as they raise the possibility that therapeutics targeting this region (e.g., neuromodulatory interventions such as transcranial magnetic stimulation) may prove widely useful in treating and potentially preventing the onset and occurrence of different presentations and subtypes of BD.

The main strength of this meta-analysis is the inclusion of a large number of experiments, demonstrating one of the largest efforts to increase power to detect underlying associations with BD and allowing us to perform numerous well-powered subgroup meta-analyses that highlighted effects specific to resting-state, emotional, and cognitive processing. However, a consequence of this strength is the extensive clinical and methodological heterogeneity that comes with including such a diverse set of experiments, as well as substantial nesting that introduces within-study bias. In response to this limitation, we developed a robustness assessment that screened for significant effects yielded by our primary inference. There were also methodological biases: resting-state experiments differed from the other conditions in that a wide mixture of methods was used, primarily employing functional connectivity (i.e., seed-to-voxel, ALFF, fALFF, ICA, ReHo, DC, FCS, ECM, VMHC), whereas cognitive and emotional conditions primarily employed GLM-based analyses of task manipulations. Another important limitation of this study was that we did not preregister the meta-analysis before conducting it (see ‘protocol and registration’ section in the Supplementary Methods).

An additional consequence of heterogeneity in included studies meant that several ad-hoc methodological and eligibility criteria needed to be specified. In light of these limitations, we made efforts to be transparent and exhaustive in our reporting of the literature search, eligibility criteria, and methods for quantitative synthesis so as to enable replication of our work, all of which may aid in the formulation of comprehensive meta-analytic methods for future studies. Furthermore, in an effort to be inclusive and comprehensive, this meta-analysis included multiple publications that came from the same laboratory, with a strong accompanying risk of the same patients being included in separate studies (see Supplementary Results). Ideally, many different research groups should contribute a significant finding, and this was generally the case, although some laboratories did contribute two or more studies. In the case of the mOFC hyperactivation finding (BD > non-clinical controls), one laboratory contributed the large majority of studies (see Supplementary Results). Intriguingly, a similar finding has been observed in a transdiagnostic meta-analysis [92], and, of the various possible reasons for this laboratory’s over-representation in identifying this effect, it may be 1) that many research groups are not routinely considering hyperactivity of the DMN in their hypothesis generation, and/or 2) that the methods employed by this laboratory are particularly sensitive to this effect. Overall, our findings may provide a basis for future replication attempts by independent laboratories, and/or more ambitious demonstrations (i.e. employing more divergent techniques) of the same phenomenon. Finally, we did not run a priori illness-specificity meta-analyses examining the effect of control group type (non-clinical vs. clinical) due to power asymmetry in included experiments (and were underpowered to conduct separate meta-analyses directly comparing participants with BD to those with unipolar depression and schizophrenia). Post-hoc analyses did not reveal significant moderation effects of mood state, diagnostic subtype, medication, psychosis, age, gender, or control group type on the primary findings, however, raising the likelihood that these neural differences are reliable across different presentations of BD and relatively robust to mood state-related effects. However, the aforementioned power asymmetry across mood states, i.e., nearly 70% of experiments sampled euthymic or depressed participants with BD, may potentially be introducing a mood state-related bias. Post-hoc mood state by task type dissociations revealing differences in SPL activation converging in euthymic BD participants during cognitive tasks and in PCC connectivity in depressed BD participants during resting-state experiments may be reflective of a potential bias. This can be mitigated in future meta-analyses by conducting more and larger studies of hypo/mania and mixed states, especially because these mood states uniquely characterize BD [94]. Thus, identifying robust mania-related markers will greatly inform our ability to elucidate a neural marker of objective risk for BD. Furthermore, there was a relatively small number of experiments using reward processing paradigms, and thus it was not possible to perform a separate analysis of these data. Future studies should determine whether these functional differences are pathognomonic of BD or illness-severity effects (hence more studies with clinical controls are needed), and whether these effects hold in larger studies of hypo/manic and mixed samples. A next stage will be to determine how the neural differences identified in the present adult BD meta-analysis compare with child onset BD, and in at-risk child and adolescent groups. This may inform early risk identification. Another future direction will be to examine effects of mood state, treatments and illness duration.

In the present work, we included a novel estimate of the underlying effect size, based on a *χ*^2^ distribution. This estimate suggested that effect sizes tended to be medium to large. This effect size estimate does not include an estimate of the effect of heterogeneity, which is likely to be substantial in this sample due to the great variety of experimental approaches which were adopted. Further work may clarify whether there is indeed a medium-large underlying effect size in the regions identified, or whether there are even larger effect sizes which are obscured by the heterogeneity of the included studies.

## Conclusions

The present study aimed to evaluate whether identification of a concordant, reproducible functional difference distinguishing BD from controls was tractable given substantial heterogeneity across experiments and found reliable, stable neural signatures that were condition-dependent. This study is the most comprehensive meta-analysis of BD functional neuroimaging studies to date. Our findings highlight core regions involved in BD that are not only context-specific, but also observed across mood states, given that our analyses were pooled across euthymic, depressed, hypo/manic and mixed mood state participants. We acknowledge, however, that these regions might more likely represent trait-like markers due to an overrepresentation of euthymic/remitted participants, which is a limitation of this meta-analytic dataset. To move towards the goal of being able to identify robust, group-level trait- and/or state-like markers of BD in future work, including future meta-analyses, better power-matched subgroup comparisons and larger subgroup analyses of different mood states are needed. These findings nevertheless build on existing neural models of BD (e.g., the OFC, amygdala, striatum, and VLPFC are components of previous models of BD [2, 4, 5]) and may help feed back onto studies that will develop new models, as our resting-state and cognition meta-analyses revealed additional condition-specific regions (e.g., the PCC, SPL, and IPL) that evolve and expand the consensus, complexities, and context of the neural circuitry implicated in BD, which can guide future hypothesis-testing [95]. Our present findings highlighting associations with BD of the amygdala and key prefrontal and parietal cortical regions in large-scale networks (e.g., the central executive network) also support other models of BD which emphasize that different mood episodes in BD might result from a progression from prefrontal cortical-subcortical differences to differences in functional coupling among largescale neural networks [96–98].

Future research should compare task and resting-state differences in the PCC and OFC, determine the neurodevelopmental trajectories of these regions in at-risk populations, and investigate whether BD medications (e.g., lithium) modulate these regions in non-clinical controls [99], which can elucidate potentially stabilizing or even normalizing [100] mechanisms of pharmacological treatments on functional differences in BD. Future studies should further examine these regions when shaping neural models of BD, and aim to identify mood state-related neural differences in BD, given that there are not enough existing studies of hypo/mania, mixed states, and clinical comparisons. Nevertheless, we hope the findings of this meta-analysis show promise for the ability of functional neuroimaging to identify group-level differences distinguishing BD that can be leveraged to advance therapeutics in the coming years.

### Supplementary information

Data Supplement
