# Supplementary material for: Prefrontal, parietal, and limbic condition-dependent differences in bipolar disorder: a large-scale meta-analysis of functional neuroimaging studies
Source: Mol Psychiatry. 2023 Feb 13;28(7):2826–38. doi: 10.1038/s41380-023-01974-8 (PMC10615766; doi:10.1038/s41380-023-01974-8)
Supplement: Supplementary file 1 — Data Supplement [file 41380_2023_1974_MOESM1_ESM.docx]

Data Supplement for Schumer et al., Prefrontal, Parietal, and Limbic Condition-Dependent Differences in Bipolar Disorder: A Large-Scale Meta-Analysis of Functional Neuroimaging Studies

**CONTENTS**

| Section / Figure / Table Title | Page Number |
| --- | --- |
| **Supplementary Methods.** Registration, Search, Inclusion Criteria, Extraction, Activation Likelihood Estimation | 5 |
| **Supplementary Results.** Exploratory Meta-Analyses, Contribution of Multiple Studies from the Same Laboratory, Post-Hoc Analyses of Clinical Factors | 15 |
| **Supplementary Table 1.** Summary of Experiments Included in the Omnibus Meta-Analysis Across All Task Types | 23 |
| **Supplementary Table 2.** Summary of Experiments Included in the Resting-State Meta-Analysis | 24 |
| **Supplementary Table 3.** Summary of Experiments Included in the Cognitive Tasks Meta-Analysis | 25 |
| **Supplementary Table 4.** Summary of Experiments Included in the Emotional Tasks Meta-Analyses | 26 |
| **Supplementary Table 5.** Summary of Experiments Included in the Valence Meta-Analyses | 27 |
| **Supplementary Figure 1.** Significant Clusters of Connectivity Differences in Depressed Bipolar Disorder Participants During Resting-State Experiments | 28 |
| **Supplementary Figure 2.** Significant Clusters of Hypoactivation in Bipolar Disorder Across Cognitive Tasks | 29 |
| **Supplementary Figure 3.** Significant Clusters of Activation Differences in Euthymic Bipolar Disorder Participants Across Cognitive Tasks | 30 |
| **Supplementary Figure 4.** Significant Clusters of Activation Differences in Hypo/manic Bipolar Disorder Participants Across Cognitive Tasks | 31 |
| **Supplementary Figure 5.** Significant Clusters of Hypoactivation in Bipolar Disorder Across Emotional Tasks | 32 |
| **Supplementary Figure 6.** Significant Clusters of Activation Differences in Euthymic Bipolar Disorder Participants Across Emotional Tasks | 33 |
| **Supplementary Figure 7.** Significant Clusters of Activation Differences in Bipolar Disorder Across Task-Based Experiments | 34 |
| **Supplementary Figure 8.** Significant Clusters of Activation Differences in Bipolar Disorder Across Working Memory Paradigms | 35 |
| **Supplementary Table 6**. Significant Clusters from Exploratory ALE Meta-Analyses | 36 |
| **Supplementary Table 7.** Studies Contributing to the Striatum Cluster from the Pooled Omnibus Meta-Analysis of 205 Studies | 37 |
| **Supplementary Table 8.** Experiments Contributing to the Striatum Cluster from the Nested Omnibus Meta-Analysis of 506 Experiments | 38 |
| **Supplementary Table 9.** Studies Contributing to the Posterior Cingulate Cortex Cluster from the Pooled Resting-State Meta-Analysis of 63 Studies | 41 |
| **Supplementary Table 10.** Experiments Contributing to the Posterior Cingulate Cortex Cluster from the Nested Resting-State Meta-Analysis of 150 Experiments | 42 |
| **Supplementary Table 11.** Experiments Contributing to the Posterior Cingulate Cortex Cluster from the Depressed Bipolar Disorder & Pooled Resting-State Meta-Analysis of 29 Studies | 43 |
| **Supplementary Table 12.** Experiments Contributing to the Posterior Cingulate Cortex Cluster from the Depressed Bipolar Disorder & Nested Resting-State Meta-Analysis of 62 Experiments | 44 |
| **Supplementary Table 13.** Studies Contributing to the Inferior Parietal Lobule, Superior Parietal Lobule, Orbitofrontal Cortex, and Striatum Clusters from the Pooled Cognitive Tasks Meta-Analysis of 69 Studies | 45 |
| **Supplementary Table 14.** Experiments Contributing to the Inferior Parietal Lobule, Superior Parietal Lobule, and Orbitofrontal Cortex Clusters from the Nested Cognitive Tasks Meta-Analysis of 134 Experiments | 47 |
| **Supplementary Table 15.** Studies Contributing to the Orbitofrontal Cortex and Ventral Anterior Cingulate Cortex Clusters from the Pooled Cognitive Tasks Meta-Analysis of Bipolar Disorder Hypoactivation Alone (27 Studies) | 49 |
| **Supplementary Table 16.** Experiments Contributing to the Orbitofrontal Cortex and Ventral Anterior Cingulate Cortex Clusters from the Nested Cognitive Tasks Meta-Analysis of Bipolar Disorder Hyperactivation Alone (40 Experiments) | 50 |
| **Supplementary Table 17.** Studies Contributing to the Premotor Cortex Cluster from the Pooled Cognitive Tasks Meta-Analysis of Bipolar Disorder Hypoactivation Alone (29 Studies) | 52 |
| **Supplementary Table 18.** Experiments Contributing to the Premotor Cortex Cluster from the Nested Cognitive Tasks Meta-Analysis of Bipolar Disorder Hypoactivation Alone (43 Experiments) | 53 |
| **Supplementary Table 19.** Studies Contributing to the Superior Parietal Lobule Cluster from the Euthymic Bipolar Disorder & Pooled Cognitive Tasks Meta-Analysis of 31 Studies | 54 |
| **Supplementary Table 20.** Experiments Contributing to the Superior Parietal Lobule Cluster from the Euthymic Bipolar Disorder & Nested Cognitive Tasks Meta-Analysis of 55 Experiments | 55 |
| **Supplementary Table 21.** Studies Contributing to the Premotor Cortex Cluster from the Hypo/manic Bipolar Disorder & Pooled Cognitive Tasks Meta-Analysis of 7 Studies | 56 |
| **Supplementary Table 22.** Experiments Contributing to the Premotor Cortex Cluster from the Hypo/manic Bipolar Disorder & Nested Cognitive Tasks Meta-Analysis of 12 Experiments | 57 |
| **Supplementary Table 23.** Studies Contributing to the Amygdala Cluster from the Pooled Emotional Tasks Meta-Analysis of 77 Studies | 58 |
| **Supplementary Table 24.** Experiments Contributing to the Amygdala Cluster from the Nested Emotional Tasks Meta-Analysis of 222 Experiments | 59 |
| **Supplementary Table 25.** Studies Contributing to the Ventrolateral Prefrontal Cortex Cluster from the Pooled Emotional Tasks Meta-Analysis of Bipolar Disorder Hypoactivation Alone (36 Studies) | 61 |
| **Supplementary Table 26.** Experiments Contributing to the Ventrolateral Prefrontal Cortex Cluster from the Nested Emotional Tasks Meta-Analysis of Bipolar Disorder Hypoactivation Alone (93 Experiments) | 62 |
| **Supplementary Table 27.** Studies Contributing to the Amygdala Cluster from the Pooled Mixed Valence Meta-Analysis of 32 Studies | 63 |
| **Supplementary Table 28.** Experiments Contributing to the Amygdala Cluster from the Nested Mixed Valence Meta-Analysis of 77 Experiments | 64 |
| **Supplementary Table 29.** Studies Contributing to the Striatum Cluster from the Euthymic Bipolar Disorder & Pooled Emotional Tasks Meta-Analysis of 39 Studies | 66 |
| **Supplementary Table 30.** Experiments Contributing to the Striatum Cluster from the Euthymic Bipolar Disorder & Nested Emotional Tasks Meta-Analysis of 101 Experiments | 67 |
| **Supplementary Table 31.** Studies Contributing to the Ventrolateral Prefrontal Cortex and Dorsal Anterior Cingulate Clusters from the Pooled Exploratory Task-Based Activation Meta-Analysis of 132 Studies | 68 |
| **Supplementary Table 32.** Studies Contributing to the Premotor Cortex and Orbitofrontal Cortex Clusters from the Pooled Exploratory Working Memory Paradigms Meta-Analysis of 28 Studies | 70 |
| **Supplementary Table 33.** Descriptions of the 205 Studies Included in the Meta-Analyses | 71 |
| **Supplementary References** | 96 |

**Supplementary Methods. Registration, Search, Inclusion Criteria, Extraction, Activation Likelihood Estimation**

**Protocol and Registration**

The time frame of our work was not appropriate for protocol preregistration on PROSPERO (www.crd.york.ac.uk/prospero/) based on their acceptance criterion established on October 1, 2019 that data extraction had to have not yet started. As our systematic literature search was conducted in July 2019, and data extraction began shortly after, the meta-analytic review did not meet the criteria to be registered. Nonetheless, this meta-analysis was conducted using the Preferred Reporting Items for Systematic Reviews and Meta-Analyses (PRISMA) guidelines(1).

**Literature Search and Eligibility Criteria**

Neuroimaging experiments using functional magnetic resonance imaging (fMRI), arterial spin labeling (ASL), fluorodeoxyglucose (FDG) and ^15^[O]H_2_O positron emission tomography (PET) were identified by PubMed during the week of July 10-18, 2019. The search strategy used the following key words in different combinations with *bipolar*, *bipolar disorder,* and *mania*: *resting-state, resting state, connectivity, functional connectivity, fMRI, functional MRI, functional imaging, functional magnetic resonance imaging, neuroimaging, cerebral blood flow, arterial spin labeling, perfusion, positron emission tomography, PET, fluorodeoxyglucose, ALFF, brain activity, brain activation,* and *functional abnormalities*. MeSH terms such as *bipolar disorder, magnetic resonance imaging, humans,* and *adults* were also used. No language or publication status restrictions were imposed on the systematic search. In light of PubMed being redesigned in 2020 and reintroduced as PubMed 2.0(2), a second search was conducted in August 2020 (using the same search terms and original search dates) to be sure that all relevant articles were captured. This included articles published online up until July 18, 2019. References of retrieved papers, relevant reviews, and previous meta-analyses on BD were also searched for additional papers not identified by the computerized search. After removing all duplicate articles, the papers retrieved from the search were screened first by title and abstract, and then by the inclusion criteria to create a definitive list of studies to be included in the meta-analysis. Additionally, for published papers in which experiments derived from whole-brain analyses were described as being collected but not reported, we contacted the study authors requesting the unpublished data.

Experiments^[[1]](#footnote-1)^ were included if they met the following criteria:

- Must compare participants diagnosed with BD (n>5) to a non-BD control group composed of either non-clinical individuals and/or another clinical cohort (e.g., unipolar depression, schizophrenia, non-clinical relatives). Studies using a mixed psychiatric sample (e.g., BD combined with unipolar depression) were excluded.
- For longitudinal studies with more than one scanning session, only baseline experimental results were reported.
- The minimum age was 16 and the mean age was over 18. While the majority of included studies measured adults aged 18 or older, a minority of studies included participants aged 16. These studies were included so as to increase the number of relevant studies in the meta-analysis and be as inclusive as possible. Pediatric (<16 years of age) and at-risk cases of BD were excluded to mitigate variability in neural activations that might be secondary to developing sex hormone effects(3–7).
- Must use fMRI, ASL, or FDG/^15^[O]H_2_O PET. We elected to use a multi-modal inclusion approach given that these modalities largely overlap in what they measure and their sensitivity(8–10), as previous ALE meta-analyses have done(11–14).
- Must report voxelwise whole-brain results of significant group differences or group x condition interactions (BD versus non-BD controls) using standard MNI or Talairach coordinates. To avoid statistical bias that would violate the assumptions of ALE (in which the null distribution reflects a random spatial association between findings across the entire brain under the assumption that each voxel has the same a priori chance of being activated(15,16)), study experiments using small-volume correction, atlas-defined regions of interest (ROIs, including post-hoc functional ROIs and ROI-based parcellations), restricted connectivity (e.g., effective connectivity within a select network), or masking (e.g., between-group analyses masked by selective significant voxels from within-group analyses) were excluded.
- Must report significant group differences or group x condition interactions using directional two-sample *t*-tests or nondirectional *F* tests. While planned *t* statistics were the preferred method for reporting between-group differences so that directionality (hyper/hypoactivation relative to BD) could be specified, experiments that modeled group differences using an analysis of variance (ANOVA) were also included and coded as nondirectional *F* statistics in the event that no planned *t*-tests were conducted. We reported *F* statistics instead of post-hoc *t*-test directional results to prevent selection bias, given that post-hoc effects are often restricted to a small subset of significant voxels to reduce the search space(17) (however, post-hoc directional effects were reported if they were unbiased i.e., conducted at the whole-brain level). For experiments that reported both planned, unbiased *t* and *F* statistics, we only reported the preferred *t* tests. Experiments in which an ANOVA was used but *F* test coordinates were not reported (only post-hoc *t-*test coordinates were shown) were considered to be incomplete reporting and thus excluded. Furthermore, in an effort to remain unbiased but also not report results ambiguous to BD (i.e., results driven by differences between other control groups or BD subgroups), for experiments reporting an ANOVA with more than one non-BD control group and/or more than one BD subgroup, at least 75% of post-hoc tests or coordinate sets, if applicable, had to be reporting significant differences between BD and non-BD controls. This percentage was chosen as it was the median percentage of post-hoc tests relevant to BD participants from eligible ANOVA papers with 3 or more groups. However, *F* test experiments including groups below age 16 were excluded, for reasons described above.

Literature search and article screening were conducted by one reviewer (MCS), who read each identified article, applied the eligibility criteria, subsequently included or excluded each article, and if included, categorized the article’s characteristics into the various subcategories for data extraction (see ‘data extraction and experimental design’ section below) accordingly. Any discrepancies in eligibility criteria (including anomalous studies) were discussed and resolved by a second judge (HWC). When data of interest (e.g., whole-brain results) were not available via the published manuscript, the study authors were contacted. The database containing citations and reason(s) for exclusion for all articles identified by the literature search can be made available by request.

To our knowledge, there were no established comprehensive criteria for assessing bias at the level of individual functional neuroimaging experiments.

**Data Extraction and Experimental Design**

Meta-analytic data—peak coordinates and study sample characteristics—were extracted from each experiment by one coder (MCS) and cross-checked by a second coder (RR) who also resolved any coding discrepancies along with a second judge (HWC).

The following information was extracted and coded in the meta-analytic database:

- - Imaging Modality: *fMRI, PET,* or *ASL*.
  - Contrast Direction: Hypoactivation *C>BD*, hyperactivation *BD>C*, or main effect of BD *F_CBD.*
  - Peak Coordinate Stereotaxic Space: Peak coordinates of experiments were reported in either MNI or Talairach space based on the software used in the study, such that analyses conducted using SPM (Statistical Parametric Mapping) after SPM96 or FSL (FMRIB Software Library), both of which use the standard MNI template, were treated as MNI coordinates unless a transformation to Talairach space was explicitly reported (i.e., via the Lancaster transform(18,19)), whereas analyses using AFNI (Analysis of Functional Images) or BrainVoyager QX(20) were reported in Talairach space(21).
  - Type of Experiment: *Resting-state, Cognitive*, or *Emotional.* Experiments in which there was an emotional manipulation on a cognitive task (e.g., emotional Stroop, emotional faces N-back, Go/NoGo with negative distractors) were coded as *Emotional* unless the cognitive contrast (e.g., 3-back>0-back) was regressed out in which case the experiment was coded as *Cognitive*.
  - Valence: Experimental task stimuli valence was coded as *negative* (e.g., fear, sadness, anger, disgust, monetary loss, pain)*, positive* (anticipation of reward, happiness, pleasure)*, mixed* (positive and negative collapsed), or *neutral* (e.g., blank face, shape). If an affective task contrast collapsed neutral with negative or positive stimuli (e.g., happy and neutral faces > baseline), valence was considered net negative or net positive, respectively.
  - Level of Arousal: Experiments were coded as *arousing* if their valence was coded as positive, negative, or mixed (as was often the case for affective task experiments), or *non-arousing* for cognitive experiments or neutral conditions of emotional experiments.
  - BD Mood State: BD samples were coded as either (exclusively) *Hypo/Manic or Mixed* (combined due to both low power and studies collapsing hypo/manic and mixed participants into one sample)*, Depressed,* or *Euthymic/Remitted,* if explicitly stated by the authors. *Euthymic* included samples described as both euthymic and/or remitted, due to the interchangeability and overlap in the description and criteria of both terms(22). Samples consisting of combined mood states or unspecified mood state (including samples described as “clinically stable” or “stabilized on medication”) were coded as *Not Specified.*
  - BD Diagnostic Subtype: Coded exclusively as *BD I, BD II, Mix* (both BD I and II), *Not Otherwise Specified (NOS,* if any of the included participants were diagnosed with BD NOS), or *Not Reported.*
  - Medication Status: *Unmedicated*^[[2]](#footnote-2)^, ≤*75% Medicated,* *>75%* *Medicated, 100% Medicated*, or *Not Reported*. The cut-off value of 75% was chosen as it was the mean percentage of the included experiment samples that were medicated. Two studies(24,25) that described their sample as “mostly” medicated (no percentage specified) were coded as *>75% Medicated.*
  - Psychosis: *All with* *Current Psychotic Features* (if current psychotic features were explicitly stated by the authors and/or if BD participants are measured by the Positive and Negative Syndrome Scale (PANSS)(26)), *All with* *History of Psychosis, Partial with Current Features* (if part of the BD sample is described as currently presenting with psychotic features), *Partial with History* (if part of the BD sample is described as having a history of psychosis), *No Current Features* (if explicitly stated by the authors that the BD sample shows no current psychotic features or if psychotic disorders were excluded), *No History* (if explicitly stated by the authors that the BD sample does not have a history of psychosis, or if psychotic disorders were excluded) or *Not Reported*. Six studies reported that their BD sample had both a history of psychosis and displayed current psychotic features; five of those studies’ experiments were coded as having current features in order to better represent the most current characteristics of the sample (if at least 2 participants displayed psychotic features), with the exception of Blumberg et al. 2003, where only 1 participant with BD displayed current psychotic features.
  - Control Group: *Non-Clinical* (if unrelated, non-clinical individuals i.e., without a psychiatric illness) or *Clinical* (having a psychiatric diagnosis e.g., unipolar or major depression, schizophrenia, ADHD, borderline personality disorder; or relatives of participants with BD). Experiments using an ANOVA model that included both non-clinical and clinical control groups with the BD group were coded as *Clinical*.
  - Age of BD Participants: *Greater* *Than* or *Less* *Than/Equal To* the median BD sample age calculated across all studies included in this meta-analysis, which was 36.4 years.
  - Gender of BD Participants: *Greater Than 50% Female* or *Less Than/Equal To 50% Female.* Gender was not reported in Adler et al. 2004(27).

For studies reporting results both with and without clinical or disorder-related covariates (e.g., medication, comorbid diagnoses, symptom scale scores)(28), results without covariates were preferentially included in this meta-analysis. However, studies only reporting results with covariates were also included, although results without covariates were preferred out of concern that including covariates such as medication could lead to suppression effects(29) because they will be strongly confounded with the disease process(30). This reporting decision did not apply to studies using common non-clinical covariates such as demographics (e.g., age, gender) and motion.

For studies in which authors sent un-thresholded, uncorrected whole-brain data (as per our request), we analyzed the data in SPM Anatomy(31) using a *t* or *z* value corresponding to *P*<0.005 and a cluster extent threshold of *k*=10 voxels (with the exception of one study’s data sent in AFNI format). Since SPM Anatomy can report up to 11 maximum peak coordinate sets, in an effort to reduce reporting bias (i.e., over-reporting repetitive coordinates) without mischaracterizing large clusters, we first reported the tallest peak with the highest *t*/*z* statistic, and then included a secondary peak if it was greater than 10 mm in any of the three (x/y/z) dimensions of the tallest peak; if it was within 10 mm in all three dimensions, it was not reported. Subsequent peaks were reported if outside the 10 mm threshold for any included peaks in any of the three dimensions.

**Activation Likelihood Estimation (ALE)**

All meta-analyses were performed using the revised ALE algorithm(32) implemented in MATLAB. ALE is random-effects method that identifies significant areas of convergence (clusters) of reported coordinates for group differences in activation during resting, cognitive, or emotional tasks that are higher than expected under a random, spatially uncertain null distribution. Reported peak coordinates (i.e., foci) are treated as centers of 3D Gaussian probability distribution that captures the spatial uncertainty associated with each focus, where the width of the Gaussian distribution is based on empirical data on the between-subject variance. The between-subject variance is weighted by experimental sample size, with larger sample sizes having a higher localizing power(33), such that experiments with a larger sample size may have a taller, more narrow peak that is more spatially precise and towards the center of the cluster, whereas an experiment with a smaller sample size will have a flatter, wider gaussian function that indicates uncertainty about the location of the foci peak and may display activation a few millimeters to the right of the cluster, hence the probability function around each foci relates to spatial precision that is determined by the smaller sample. In the present meta-analysis, when the sample sizes of the BD and control group(s) were unequal, we chose to weight the between-subject variance by the smaller group’s sample size for a statistically tighter comparison. The probability distributions for all foci of a given experiment are then aggregated into a modeled activation map, giving a voxelwise ALE score that is the experiment’s modeled activation map’s given focus (i.e., spatial coordinates of the peak voxels) which incorporates the spatial uncertainty around each focus. The union of all modeled activation maps across all experiments results in voxelwise ALE scores designating particular locations across the brain where experimental results converge, and these scores at points of convergence are then tested against an analytically derived null-distribution reflecting a random spatial association between experiments so as to differentiate areas of true convergence from random overlap. The subsequent application of a random-effects inference assesses for above-chance convergence between experiments, such that observed ALE scores are tested against the expected ALE scores under the null distribution, which is formed by randomly sampling voxels from each of the modeled activation maps and then taking the union of these randomly sampled values. The resulting non-parametric *p* values in each meta-analysis were then thresholded at *P*<0.05 family-wise error (FWE) cluster-level corrected (cluster-forming threshold at voxel-level *P*<.001), and *z-*transformed for statistical images. In the present meta-analysis, the initial, primary approach pooled together all coordinates from all contrasts from a given study into one contributing experiment. For the secondary robustness assessment, each study contrast was treated as a separate experiment; in this method, a study may provide multiple experiments; such studies were considered nested and could thus contribute more than once. For seed-based studies reporting multiple seeds, each seed (including bilateral seeds) was coded as a separate experiment for the nested analysis.

**The ALE Contribution Score**

We conducted a two-part robustness assessment that tested the degree to which meta-analytic results yielded by the primary, pooled method were robust to the impact of nesting using an ALE summary statistic computed for each cluster’s ALE contribution analysis, the non-linear contribution score, to determine which nested experiments to remove. Each experiment that contributes to a significant cluster is given an average contribution score. The statistic is computed from the ratio of ALE scores at the location of the significant cluster with and without the contributing experiment. An experiment with a low contribution score indicates that removing the experiment from the analysis will have little impact on the ALE score, whereas higher scores indicate that the ALE score is driven more strongly by those experiments. Removal of a low-powered, more spatially uncertain experiment that is peripherally related to the cluster thus may not have an effect on the significant cluster volume; these experiments will thus have lower contribution scores.

**Supplementary Results. Exploratory Meta-Analyses, Contribution of Multiple Studies from the Same Laboratory, Post-Hoc Analyses of Clinical Factors**

The final set of experiments consisted of 205 published studies with 506 individual neuroimaging experiments (yielding 3112 foci total when nested experiments were included, and 2910 foci when coordinates were pooled). Foci per individual experiment ranged from 1 to 68 (mean=6.15, median=4). Sample size ranged from 10 to 310 (mean=67.16, median=50). The mean age of participants with BD in each study ranged from 22.59 to 58.7 (mean=36.96, median=36.4).

**Exploratory Meta-Analyses (Supplementary Table 6)**

Five exploratory meta-analyses were conducted: 1) experiments measuring task-based activation; 2) experiments measuring seed-based resting-state functional connectivity; 3) working memory paradigms; 4) emotional reactivity paradigms; and 5) emotion regulation paradigms. Given that these analyses were exploratory and thus beyond the present study’s scope of a priori objectives and interests, these meta-analyses were performed using the primary pooled approach and no further post-hoc tests were conducted.

The meta-analysis across task-based activation experiments (number of included experiments (NIE)=132) revealed significant convergence in the right ventrolateral prefrontal cortex (VLPFC) and bilateral dorsal anterior cingulate cortex extending to the medial orbitofrontal cortex (mOFC) (Supplementary Figure 7; see Supplementary Table 31 for contributing experiments).

The meta-analysis across seed-based resting-state functional connectivity experiments (NIE=27) did not reveal significant results.

The meta-analysis across working memory paradigms (NIE=28) revealed significant convergence in two regions: the left premotor/supplementary motor cortex and the left mOFC (Supplementary Figure 8; see Supplementary Table 32 for contributing experiments).

The meta-analyses across emotional reactivity paradigms (NIE=63, with emotional reactivity defined as the experience of or response to an emotion or emotional cue) and emotion regulation paradigms (NIE=17, with emotion regulation defined as cognitive control deployed specifically for the regulation of emotion (e.g. the suppression or amplification of emotional experience, or a heightened control of attention in the presence of emotional cues), as compared to cognitive control in non-emotional contexts, or conditions, emotional or otherwise, without cognitive control requirements.) did not reveal significant results.

**Contribution of Multiple Studies from the Same Laboratory on the Primary Findings**

For the 6 findings passing both the primary, pooled test and the subsequent nested robustness assessment, we examined the contribution of overlapping study samples (i.e., multiple studies whose samples come from the same research group/laboratory). Of the 13 resting-state studies contributing to differences in BD in functional connectivity of the posterior cingulate cortex (PCC), 5 came from Jinan University in Guangzhou, China, authored by Y. Wang, Y. Jia, L. Huang, and colleagues. Of the 9 studies contributing to differences in the inferior parietal lobule (IPL) activation across cognitive tasks, 2 came from the Vulnerability to Bipolar Disorders Study (VIBES) cohort, by S. Frangou and colleagues. Of the 8 studies contributing to differences in the superior parietal lobule (SPL) activation, 2 came from the VIBES cohort, by S. Frangou and colleagues. Of the 6 studies contributing to mOFC hyperactivation in BD across cognitive tasks, 4 came from E. Pomarol-Clotet, S. Sarró, and colleagues. Of the 20 studies contributing to differences in amygdala activation across emotional tasks, 2 came from M.L. Phillips’ laboratory; 2 from W.A. Cunningham, J. Gruber, and colleagues; 4 from G.S. Malhi’s laboratory; 2 from L.L. Altshuler, J. Townsend, and colleagues; and 2 from E.T. Bullmore, B.R. Lennox, and colleagues. Of the 11 studies contributing to differences in amygdala activation specific to emotional tasks using mixed valence manipulations, 3 came from M.L. Phillips’s laboratory; 2 from W.A. Cunningham, J. Gruber, and colleagues; and 2 from G.S. Malhi’s laboratory.

**Post-Hoc Analyses of Clinical Factors (Fisher’s Exact Tests of Independence)**

Post-hoc Fisher’s Exact tests of independence assessed whether clinical factors such as directionality, mood state, BD diagnostic subtype, medication status, psychosis, age and gender of participants with BD, and control group type confounded significant findings of interest via examining whether the clinical characteristics of contributing experiments were higher or lower than the base rate of experiments of a particular factor subgroup. Results of all tests were FDR-corrected, although uncorrected *p* values are reported alongside FDR-corrected *q* values below.

**Fisher’s Exact Tests for Meta-Analysis Across All Experiments (Omnibus)**

There were no significant effects at FDR-corrected thresholds of clinical and demographic variables on striatal activation differences in BD observed across all task types. Nonetheless, there was a marginally significant association with medication status (*q*=0.504, *p*=0.03), where BD samples that were 100% medicated were overrepresented in the cluster (57.57%) compared to the base rate (43.67%), samples that were greater than 75% medicated were underrepresented (21.21%) compared to the base rate (29.45%), samples that were less than 75% medicated were overrepresented in the cluster (15.15%) compared to the base rate (7.51%), and unmedicated samples were underrepresented (3.03%) compared to the base rate (17%). There were no significant associations between striatal activation and mood state (*q=*0.95, *p*=0.78), BD diagnostic subtype (*q=*0.938; *p*=0.67), psychosis (*q=*0.971, *p*=0.74), control group type (*q=*0.91, *p*=0.52), age (*q=*0.535; *p=*0.14), gender (*q=*0.683, *p*=0.26), or directionality (*q=*0.921, *p*=0.57), where the contribution of experiments with BD hyper/hypoactivation was relatively equal (see Supplementary Table 8).

**Fisher’s Exact Tests for Meta-Analyses Across Resting-State Experiments**

There were no significant associations between connectivity differences in the right PCC in BD and directionality (*q=*0.822, *p*=0.46), mood state (*q=*0.49, *p=0.*07), medication status (*q=*0.738, *p*=0.36), BD diagnostic subtype (*q=*0.84, *p*=0.46), psychosis (*q=*0.965, *p*=0.77), control group type (*q=*0.951, *p*=0.60), age (*q=*0.69, *p*=0.23), or gender (*q=*0.557, *p*=0.053) (see Supplementary Table 10).

**Fisher’s Exact Tests for Meta-Analyses Across Cognitive Experiments**

There were no significant effects at FDR-corrected thresholds of clinical and demographic variables on differences in mOFC activation observed across cognitive tasks in BD relative to controls. Nonetheless, there was a marginally significant association with directionality (*q=*0.48, *p=*0.04), where experiments in which individuals with BD showed hyperactivation were overrepresented (55.56%) compared to the base rate (29.85%). There were no significant associations between the mOFC and mood state (*q=*0.672, *p*=0.24), medication (*q/p*>0.99), BD diagnostic subtype (*q=*0.78, *p*=0.39), psychosis (*q=*0.954, *p*=0.67), control group type (*q/p*>0.99), age (*q=*0.989, *p*=0.73), or gender (*q=*0.969, *p*=0.75) (see Supplementary Table 14).

There were no significant associations between differences in IPL activation across cognitive tasks in BD relative to controls and mood state (*q=*0.987, *p*=0.67), medication status (*q=*0.537, *p*=0.115), BD diagnostic subtype (*q=*0.544, *p=0*.11), psychosis (*q=*0.970, *p*=0.67), control group type (*q=*0.711, *p*=0.22), age (*q/p*>0.99), gender (*q/p*>0.99), or directionality (*q>*0.99, *p* =0.91), where there was a non-significant tendency towards hypoactivation in BD (see Supplementary Table 14).

There were also no significant associations between the differences in SPL activation across cognitive tasks in BD relative to controls and mood state (*q=*0.741, *p*=0.30), medication (*q=*0.504, *p*=.06), BD diagnostic subtype (*q=*0.588, *p*=0.14), psychosis history (*q=*0.452, *p*=0.07), control group type (*q*/*p*>0.99), age (*q=*0.448, *p*=0.08), gender (*q=*0.56, *p*=0.14), or directionality (*q/p*>0.99), where there was a non-significant tendency towards hypoactivation in BD (see Supplementary Table 14).

BD hyperactivation in the left mOFC across cognitive tasks was not significantly associated with mood state (*q=*0.584, *p*=0.16), medication status (*q=*0.749, *p*=0.33), BD diagnostic subtype (*q=*0.975, *p*=0.65), psychosis (*q=*0.595, *p*=0.17), control group type (*q=*0.747, *p*=0.32), age (*q=*0.739, *p*=0.22), or gender (*q/p*>0.99) (see Supplementary Table 16).

There were no significant effects at FDR-corrected thresholds of clinical and demographic variables on BD hyperactivation in the left vACC observed across cognitive tasks in BD. Nonetheless, there was a marginally significant association with gender *(q=*0.56, *p=*0*.*04), where experiments with more female BD participants (27.27%) were underrepresented compared to the base rate (55%), and experiments with fewer female BD participants (72.73%) were overrepresented compared to the base rate (42.5%). There were no significant associations between the left vACC and mood state (*q=*0.977, *p*=0.64), medication status (*q=*0.677, *p*=0.25), BD diagnostic subtype (*q=*0.922, *p*=0.56), psychosis (*q=*0.756, *p*=0.36), control group type (*q/p*>.99), or age (*q=*0.56, *p*=0.06) (see Supplementary Table 16).

There were no significant effects at FDR-corrected thresholds of clinical and demographic variables on BD hypoactivation in the left premotor cortex observed across cognitive tasks in BD. Nonetheless, there was a significant association with mood state (*q=*0.252, *p*=0.003), such that the number of BD samples that were hypo/manic was overrepresented (50%) compared to the base rate (14%), as was the number of depressed BD samples (50%) compared to the base rate (25%), and the number of euthymic samples (0%) was underrepresented compared to the base rate (42%). There was also a significant association between premotor cortex hypoactivation and psychosis (*q=*0.168, *p*=0.004), such that the number of contributing BD samples with current psychotic features (50%) was larger than the base rate (12%), as was the number of contributing BD samples that partially had a history of psychosis (33.33%) compared to the base rate (14%); the number of BD samples that did not report information about psychosis was underrepresented (16.67%) compared to the base rate (70%), as was the number of BD samples in which part of the sample displayed psychotic features (contribution: 0%, base rate: 4%). Finally, there was a marginally significant association between premotor cortex hypoactivation and age (*q=*0.42, *p*=0.02), such that experiments with older BD participants were overrepresented (100%) compared to the base rate (51.16%) and experiments with younger BD participants were underrepresented (0%) compared to the base rate (48.84%). There were no significant associations between the premotor cortex and medication status (*q=*0.821, *p*=0.43), BD diagnostic subtype (*q=*0.535, *p*=0.07), control group type (*q/p*>.99) or gender (*q/p*>.99) (see Supplementary Table 18).

**Fisher’s Exact Tests for Meta-Analyses Across Emotional Experiments**

There were no significant associations between differences in BD in activation in the left amygdala across all emotional experiments and mood state (*q=*0.948, *p*=0.79), medication status (*q=*0.575, *p*=0.13), BD diagnostic subtype (*q=*0.987, *p*=0.74), psychosis (*q*=0.578, *p*=0.11), control group type (*q=*0.98, *p*=0.63), age (*q>*0.99, *p*=0.84), gender (*q=*0.95, *p*=0.69), or directionality (*q*=0.695, *p*=0.24), where there was a non-significant trend towards hyperactivation in BD (see Supplementary Table 24).

There were no significant associations between BD hypoactivation in the right VLPFC across all emotional experiments and mood state (*q*=0.716, *p*=0.23), medication status (*q=*0.48, *p*=0.08), BD diagnostic subtype (*q/p*>0.99), psychosis (*q=*0.909, *p*=0.53), control group type (*q=*0.774, *p*=0.35), age (*q=*0.98, *p*=0.77), or gender (*q=*0.924, *p*=0.55) (see Supplementary Table 26).

There were no significant effects at FDR-corrected thresholds of clinical and demographic variables on differences in left amygdala activation observed across mixed valence manipulations in BD. Nonetheless, there was a marginally significant association between mixed valence amygdala activation and medication status (*q=*0.168, *p*=0.006), such that BD samples that were 100% medicated were overrepresented (87.5%) compared to the base rate (60%) as well as samples that were less than 75% medicated (6.25%) compared to the base rate (4%), whereas samples that were greater than 75% medicated were underrepresented (0%) compared to the base rate (23%); the contribution of unmedicated samples (0%) was relatively similar to the base rate (1%); and the contribution of samples where medication was not reported (6.25%) was larger than the base rate (4%). There were no significant associations between the amygdala and mood state (*q=*0.744, *p*=0.31), BD diagnostic subtype (*q=*0.738, *p*=0.29), psychosis (*q=*0.754, *p*=0.35), control group type (*q/p*>0.99), age (*q=*0.82, *p*=0.42), gender (*q=*0.964, *p*=0.78), or directionality (*q=*0.803, *p*=.43), where the contribution of experiments with BD hyper/hypoactivation was relatively equal (see Supplementary Table 28).

**Summary**

While there were no effects reaching FDR-corrected significance, the only effect considered significant at an uncorrected threshold for a cluster passing both the pooled and nested tests was the effect of medication on mixed valence amygdala activation, *p*=0.006. Of the clusters that were only observed in the pooled analysis and did not survive the nested test, there were six effects considered significant at uncorrected thresholds. The left premotor cortex, hypoactive in BD during cognitive tasks, was moderated by mood state, *p*=0.003, psychosis, *p*=0.004, and age, *p*=0.02. Altered activation in the right striatum across all experiments was moderated by medication, *p*=0.03. Altered activation in the left mOFC across cognitive tasks was moderated by directionality, *p*=0.04, such that it was hyperactive in participants with BD. Hyperactivation of the left vACC in BD across cognitive tasks was moderated by gender, *p*=0.04.

**Supplementary Table 1.** Summary of Experiments Included in the Omnibus Meta-Analysis Across All Task Types*

*This table describes the overall data considered for this meta-analysis in order to depict all of the included experiments’ relevant characteristics (thus there is nesting).

| **Experiment Sample/Design Characteristics** | **All Experiments (506 Contributing Experiments)**  **No. (%)** |
| --- | --- |
| Imaging Modality  FMRI  PET  ASL | 464 (91.7)  41 (8.1)  1 (0.2) |
| Contrast Direction  BD > Control  Control > BD  Main effect/Interaction | 158 (31.23)  188 (37.15)  160 (31.62) |
| Mood State  Euthymic/Remitted  Depressed  Hypo/Manic or Mixed  Not Specified/Combined | 201 (39.72)  147 (29.05)  40 (7.91)  118 (23.32) |
| Medication Status  100% Medicated  >75% Medicated  <75% medicated  Unmedicated  Not Reported | 221 (43.67)  149 (29.45)  38 (7.51)  86 (17)  12 (2.37) |
| BD Diagnostic Subtype  BD I  BD II  BD I/II  BD NOS  Not Reported | 263 (51.98)  34 (6.72)  84 (16.6)  32 (6.32)  93 (18.38) |
| Psychosis  100% Currently Psychotic  Partial Currently Psychotic  None Currently Psychotic  100% with Psychosis History  Partial with Psychosis History  None with History  Not Reported | 31 (6.13)  27 (5.34)  55 (10.87)  19 (3.75)  66 (13.04)  26 (5.14)  282 (55.73) |
| Control Group  Non-Clinical vs. BD  Clinical vs. BD  Non-Clinical/Clinical | 376 (74.31)  66 (13.04)  64 (12.65) |
| Age  Above Median Age  Below/Equal to Median Age | 209 (41.3)  297 (58.7) |
| Gender  Greater Than 50% Female  Less Than/Equal to 50% Female  Not Reported | 251 (49.6)  254 (50.2)  1 (.2) |

Abbreviations: ASL = Arterial Spin Labeling; BD = Bipolar Disorder; FMRI = Functional Magnetic Resonance Imaging; No. = Number; NOS = Not Otherwise Specified; PET = Positron Emission Tomography.

**Supplementary Table 2.** Summary of Experiments Included in the Resting-State Meta-Analysis*

*This table describes the overall data considered for this meta-analysis in order to depict all of the included experiments’ relevant characteristics (thus there is nesting).

| **Experiment Sample/Design Characteristics** | **All Resting-State (150 Contributing Experiments)**  **No. (%)** |
| --- | --- |
| Imaging Modality  FMRI  PET  ASL | 131 (87.33)  18 (12)  1 (0.66) |
| Contrast Direction  BD > Control  Control > BD  Main effect/Interaction | 51 (34)  52 (34.67)  47 (31.33) |
| Mood State  Euthymic/Remitted  Depressed  Hypo/Manic or Mixed  Not Specified/Combined | 45 (30)  62 (41.33)  4 (2.67)  39 (26) |
| Medication Status  100% Medicated  >75% Medicated  <75% medicated  Unmedicated  Not Reported | 53 (35.33)  61 (40.67)  4 (2.66)  31 (20.67)  1 (0.67) |
| BD Diagnostic Subtype  BD I  BD II  BD I/II  BD NOS  Not Reported | 45 (30)  24 (16)  33 (22)  2 (1.33)  46 (30.67) |
| Psychosis  100% Currently Psychotic  Partial Currently Psychotic  None Currently Psychotic  100% with Psychosis History  Partial with Psychosis History  None with History  Not Reported | 10 (6.67)  5 (3.33)  1 (0.66)  10 (6.67)  20 (13.33)  19 (12.67)  85 (56.67) |
| Control Group  Non-Clinical vs. BD  Clinical vs. BD  Non-Clinical/Clinical | 99 (66)  22 (14.67)  29 (19.33) |
| Age  Above Median Age  Below/Equal to Median Age | 44 (29.33)  106 (70.67) |
| Gender  Greater Than 50% Female  Less Than/Equal to 50% Female  Not Reported | 69 (46)  81 (54)  0 (0) |

Abbreviations: ASL = Arterial Spin Labeling; BD = Bipolar Disorder; FMRI = Functional Magnetic Resonance Imaging; No. = Number; NOS = Not Otherwise Specified; PET = Positron Emission Tomography.

**Supplementary Table 3.** Summary of Experiments Included in the Cognitive Tasks Meta-Analysis*

*This table describes the overall data considered for this meta-analysis in order to depict all of the included experiments’ relevant characteristics (thus there is nesting).

| **Experiment Sample/Design Characteristics** | **All Cognitive Tasks (134 Contributing Experiments)**  **No. (%)** | **BD Hyperactivation (40 Contributing Experiments)**  **No. (%)** | **BD Hypoactivation (43 Contributing Experiments)**  **No. (%)** |
| --- | --- | --- | --- |
| Imaging Modality  FMRI  PET  ASL | 111 (82.84)  23 (17.16)  0 (0) | 39 (97)  1 (3)  0 (0) | 36 (84)  7 (16)  0 (0) |
| Contrast Direction  BD > Control  Control > BD  Main effect/Interaction | 40 (29.85)  43 (32.09)  51 (38.06) | 40 (100)  0  0 | 0 (0)  43 (100)  0 |
| Mood State  Euthymic/Remitted  Depressed  Hypo/Manic or Mixed  Not Specified/Combined | 55 (41.04)  41 (30.6)  13 (9.7)  25 (18.66) | 14 (35)  15 (38)  6 (15)  5 (12) | 18 (42)  11 (25)  6 (14)  8 (19) |
| Medication Status  100% Medicated  >75% Medicated  <75% medicated  Unmedicated  Not Reported | 62 (46.27)  23 (17.16)  17 (12.69)  31 (23.13)  1 (0.75) | 22 (55)  9 (23)  3 (7)  6 (15)  0 (0) | 21 (49)  9 (21)  3 (7)  10 (23)  0 (0) |
| BD Diagnostic Subtype  BD I  BD II  BD I/II  BD NOS  Not Reported | 79 (58.96)  5 (3.73)  25 (18.66)  2 (1.49)  23 (17.16) | 24 (60)  1 (3)  3 (7)  0 (0)  12 (30) | 29 (67)  3 (7)  4 (9)  0 (0)  7 (16) |
| Psychosis  100% Currently Psychotic  Partial Currently Psychotic  None Currently Psychotic  100% with Psychosis History  Partial with Psychosis History  None with History  Not Reported | 12 (8.96)  5 (3.73)  2 (1.49)  3 (2.24)  29 (21.64)  0 (0)  83 (61.94) | 5 (12)  2 (5)  0 (0)  2 (5)  9 (23)  0 (0)  22 (55) | 5 (12)  2 (4)  0 (0)  0 (0)  6 (14)  0 (0)  30 (70) |
| Control Group  Non-Clinical vs. BD  Clinical vs. BD  Non-Clinical/Clinical | 101 (75.37)  12 (8.96)  21 (15.67) | 34 (85)  6 (15)  0 (0) | 38 (88)  5 (12)  0 (0) |
| Age  Above Median Age  Below/Equal to Median Age | 78 (58.2)  56 (41.8) | 26 (65)  14 (35) | 22 (51.16)  21 (48.84) |
| Gender  Greater Than 50% Female  Less Than/Equal to 50% Female  Not Reported | 62 (46.27)  71 (52.98)  1 (.75) | 22 (55)  17 (42.5)  1 (2.5) | 18 (41.86)  25 (58.14)  0 (0) |

Abbreviations: ASL = Arterial Spin Labeling; BD = Bipolar Disorder; FMRI = Functional Magnetic Resonance Imaging; n = Sample Size; No. = Number; NOS = Not Otherwise Specified; PET = Positron Emission Tomography.

**Supplementary Table 4.** Summary of Experiments Included in the Emotional Tasks Meta-Analyses*

*This table describes the overall data considered for this meta-analysis in order to depict all of the included experiments’ relevant characteristics (thus there is nesting).

| **Experiment Sample/Design Characteristics** | **All Emotional Tasks (222 Contributing Experiments)**  **No. (%)** | **BD Hyperactivation**  **(67 Contributing Experiments)**  **No. (%)** | **BD Hypoactivation**  **(93 Contributing Experiments)**  **No. (%)** |
| --- | --- | --- | --- |
| Imaging Modality  FMRI  PET  ASL | 222 (100)  0 (0)  0 (0) | 67 (100)  0 (0)  0 (0) | 93 (100)  0 (0)  0 (0) |
| Contrast Direction  BD > Control  Control > BD  Main effect/Interaction | 67 (30)  93 (42)  62 (28) | 67 (100)  0 (0)  0 (0) | 0 (0)  93 (100)  0 (0) |
| Mood State  Euthymic/Remitted  Depressed  Hypo/Manic or Mixed  Not Specified/Combined | 101 (46)  44 (20)  23 (10)  54 (24) | 28 (42)  17 (25)  10 (15)  12 (18) | 38 (41)  21 (22)  11 (12)  23 (25) |
| Medication Status  100% Medicated  >75% Medicated  <75% medicated  Unmedicated  Not Reported | 106 (48)  65 (29)  17 (8)  24 (11)  10 (4) | 33 (49)  20 (30)  4 (6)  4 (6)  6 (9) | 48 (52)  19 (20)  7 (8)  15 (16)  4 (4) |
| BD Diagnostic Subtype  BD I  BD II  BD I/II  BD NOS  Not Reported | 139 (63)  5 (2)  26 (12)  28 (13)  24 (11) | 44 (66)  2 (3)  8 (12)  3 (4)  10 (15) | 45 (48)  3 (3)  9 (10)  25 (27)  11 (12) |
| Psychosis  100% Currently Psychotic  Partial Currently Psychotic  None Currently Psychotic  100% with Psychosis History  Partial with Psychosis History  None with History  Not Reported | 9 (4)  17 (8)  52 (23)  6 (3)  17 (8)  7 (3)  114 (51) | 4 (6)  6 (9)  20 (30)  0 (0)  2 (3)  3 (4)  32 (48) | 5 (5)  7 (8)  17 (18)  4 (4)  1 (1)  3 (3)  56 (60) |
| Control Group  Non-Clinical vs. BD  Clinical vs. BD  Non-Clinical/Clinical | 176 (79)  32 (14)  14 (6) | 53 (79)  14 (21)  0 (0) | 82 (88)  11 (12)  0 (0) |
| Age  Above Median Age  Below/Equal to Median Age | 87 (39)  135 (61) | 22 (33)  45 (67) | 40 (43)  53 (57) |
| Gender  Greater Than 50% Female  Less Than/Equal to 50% Female  Not Reported | 120 (54)  102 (46)  0 (0) | 33 (49)  34 (51)  0 (0) | 35 (38)  58 (62)  0 (0) |

Abbreviations: ASL = Arterial Spin Labeling; BD = Bipolar Disorder; FMRI = Functional Magnetic Resonance Imaging; n = Sample Size; No. = Number; NOS = Not Otherwise Specified; PET = Positron Emission Tomography.

**Supplementary Table 5.** Summary of Experiments Included in the Valence Meta-Analyses*

*This table describes the overall data considered for this meta-analysis in order to depict all of the included experiments’ relevant characteristics (thus there is nesting).

| **Experiment Sample/Design Characteristics** | **Mixed Valence**  **(77 Contributing Experiments)**  **No. (%)** | **Negative Valence**  **(91 Contributing Experiments)**  **No. (%)** | **Positive Valence**  **(49 Contributing Experiments)**  **No. (%)** |
| --- | --- | --- | --- |
| Imaging Modality  FMRI  PET  ASL | 77 (100)  0 (0)  0 (0) | 91 (100)  0 (0)  0 (0) | 49 (100)  0 (0)  0 (0) |
| Contrast Direction  BD > Control  Control > BD  Main effect/Interaction | 26 (34)  32 (41)  19 (25) | 26 (29)  36 (39)  29 (32) | 13 (26)  22 (45)  14 (29) |
| Mood State  Euthymic/Remitted  Depressed  Hypo/Manic or Mixed  Not Specified/Combined | 30 (39)  17 (22)  11 (14)  19 (25) | 43 (47)  13 (14)  9 (10)  26 (29) | 24 (49)  14 (29)  3 (6)  8 (16) |
| Medication Status  100% Medicated  >75% Medicated  <75% medicated  Unmedicated  Not Reported | 46 (60)  24 (31)  3 (4)  1 (1)  3 (4) | 35 (38)  25 (28)  13 (14)  14 (15)  4 (4) | 24 (49)  13 (27)  1 (2)  9 (18)  2 (4) |
| BD Diagnostic Subtype  BD I  BD II  BD I/II  BD NOS  Not Reported | 36 (47)  1 (1)  14 (18)  13 (17)  13 (17) | 65 (71)  3 (3)  7 (8)  9 (10)  7 (8) | 34 (69)  1 (2)  4 (8)  6 (12)  4 (8) |
| Psychosis  100% Currently Psychotic  Partial Currently Psychotic  None Currently Psychotic  100% with Psychosis History  Partial with Psychosis History  None with History  Not Reported | 0 (0)  8 (10)  17 (22)  4 (5)  4 (5)  0 (0)  44 (57) | 8 (9)  6 (7)  14 (15)  1 (1)  10 (11)  3 (3)  49 (54) | 1 (2)  3 (6)  19 (39)  1 (2)  3 (6)  2 (4)  20 (41) |
| Control Group  Non-Clinical vs. BD  Clinical vs. BD  Non-Clinical/Clinical | 56 (73)  18 (23)  3 (4) | 75 (82)  7 (8)  9 (10) | 40 (82)  7 (14)  2 (4) |
| Age  Above Median Age  Below/Equal to Median Age | 41 (53.25)  36 (46.75) | 39 (42.86)  52 (57.14) | 6 (12.24)  43 (87.76) |
| Gender  Greater Than 50% Female  Less Than/Equal to 50% Female  Not Reported | 40 (51.95)  37 (48.05)  0 (0) | 50 (54.95)  41 (45.05)  0 (0) | 28 (57.14)  21 (42.86)  0 (0) |

Abbreviations: ASL = Arterial Spin Labeling; BD = Bipolar Disorder; FMRI = Functional Magnetic Resonance Imaging; n = Sample Size; No. = Number; NOS = Not Otherwise Specified; PET = Positron Emission Tomography.

**Supplementary Figure 1.** Significant Clusters of Connectivity Differences in Depressed Bipolar Disorder Participants During Resting-State Experiments

**
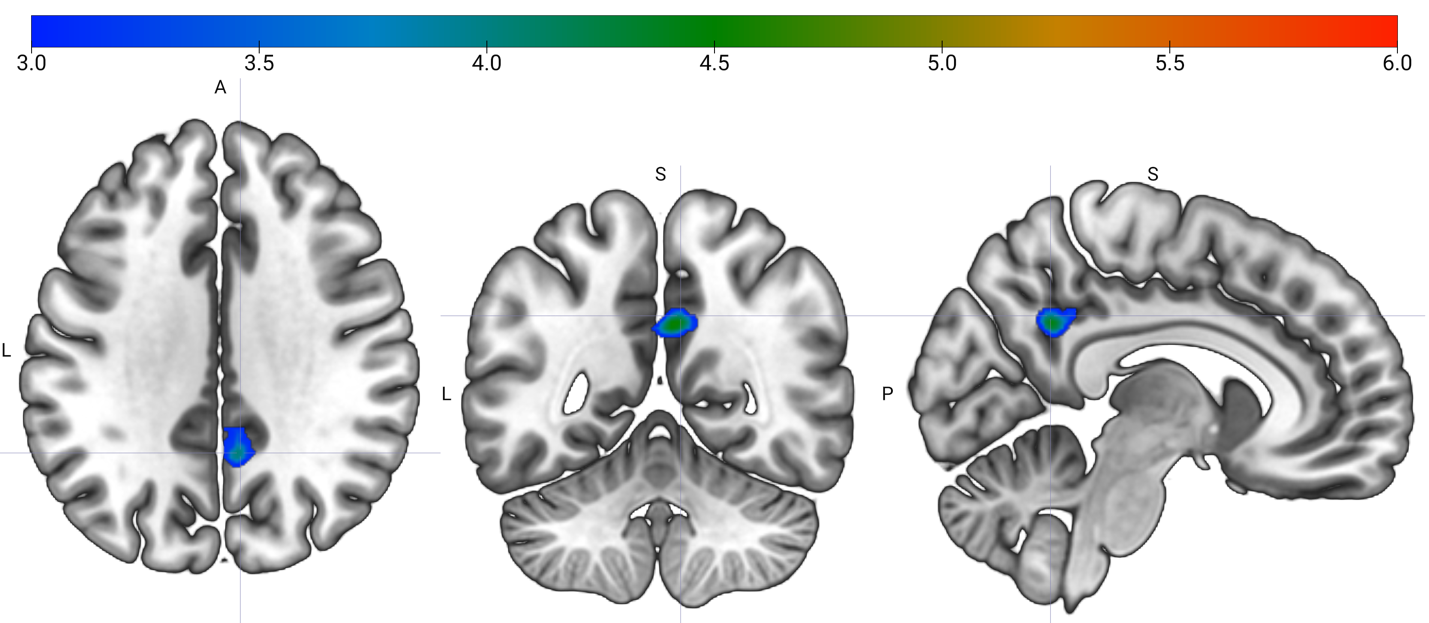
**

**Supplementary Figure 2.** Significant Clusters of Hypoactivation in Bipolar Disorder Across Cognitive Tasks

**
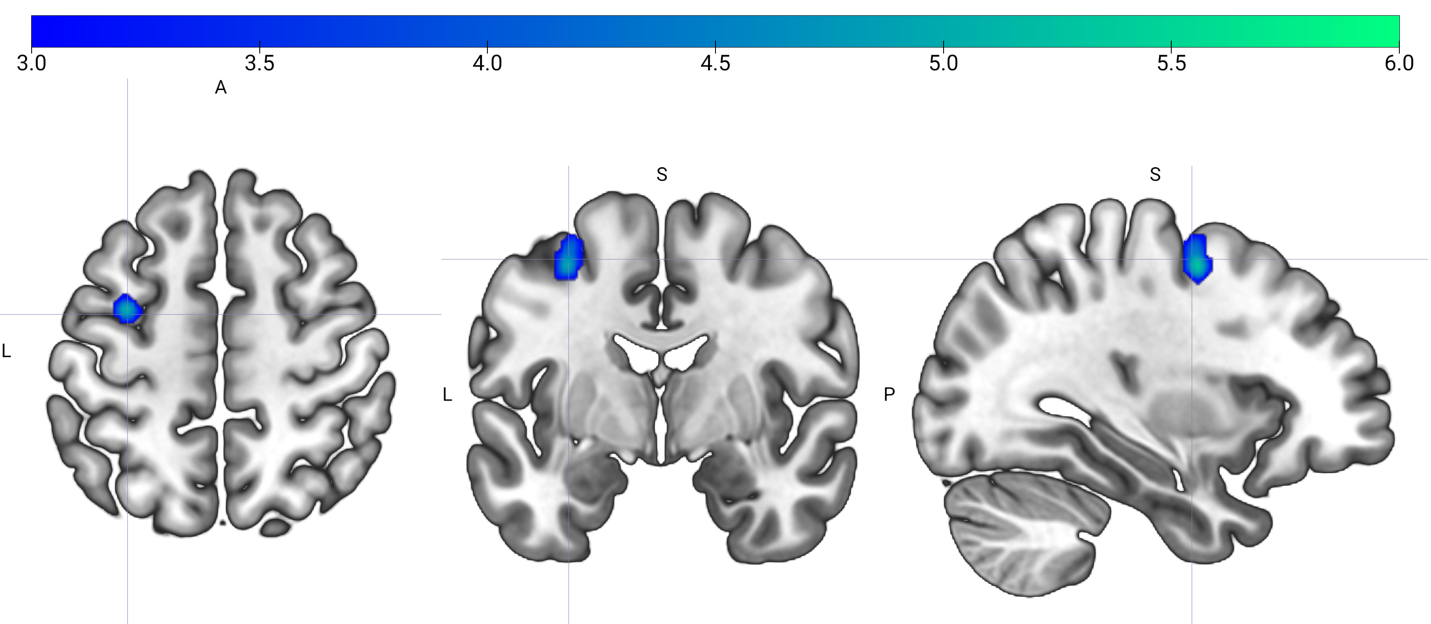
**

**Supplementary Figure 3.** Significant Clusters of Activation Differences in Euthymic Bipolar Disorder Participants Across Cognitive Tasks


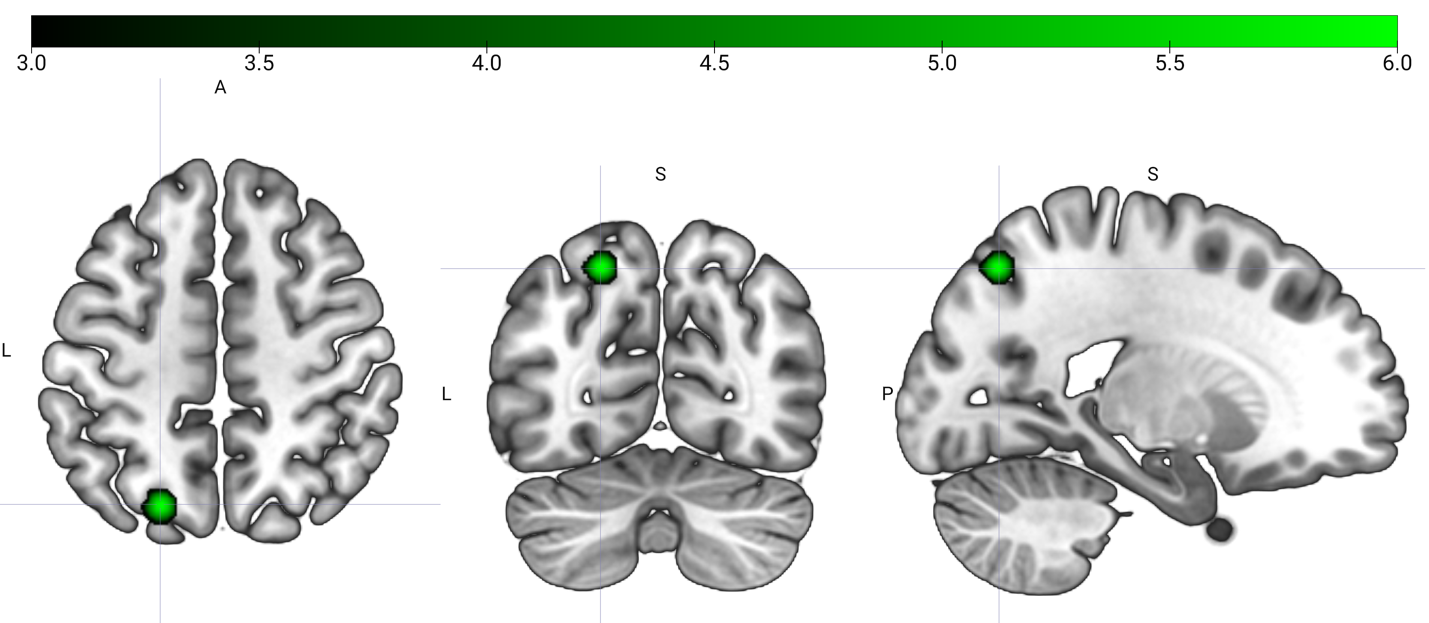


**Supplementary Figure 4.** Significant Clusters of Activation Differences in Hypo/manic Bipolar Disorder Participants Across Cognitive Tasks

**
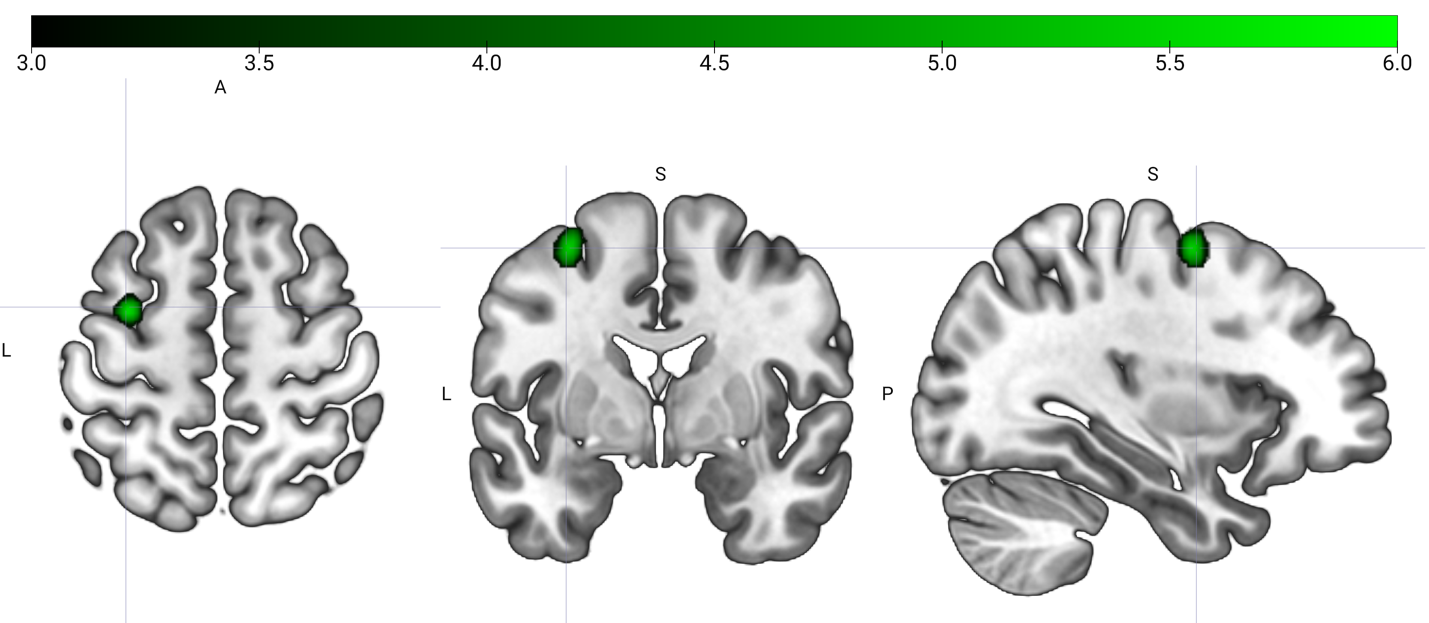
**

**Supplementary Figure 5.** Significant Clusters of Hypoactivation in Bipolar Disorder Across Emotional Tasks

**
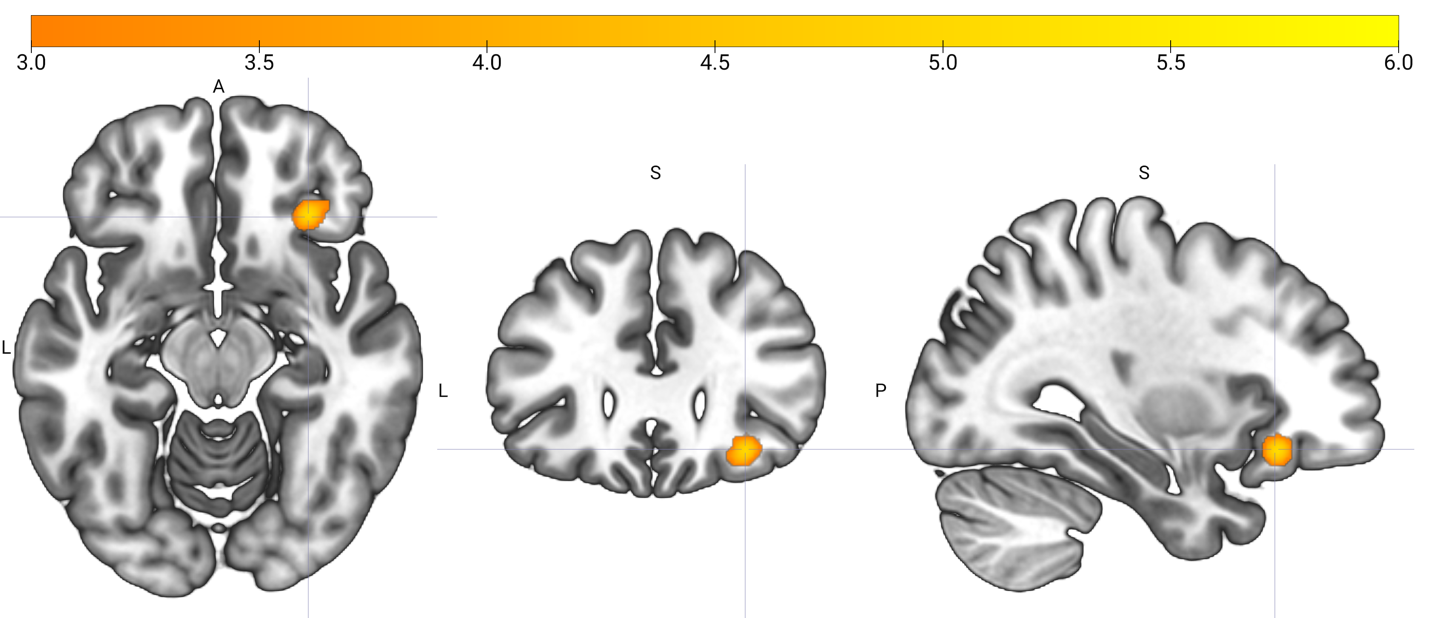
**

**Supplementary Figure 6.** Significant Clusters of Activation Differences in Euthymic Bipolar Disorder Participants Across Emotional Tasks

**
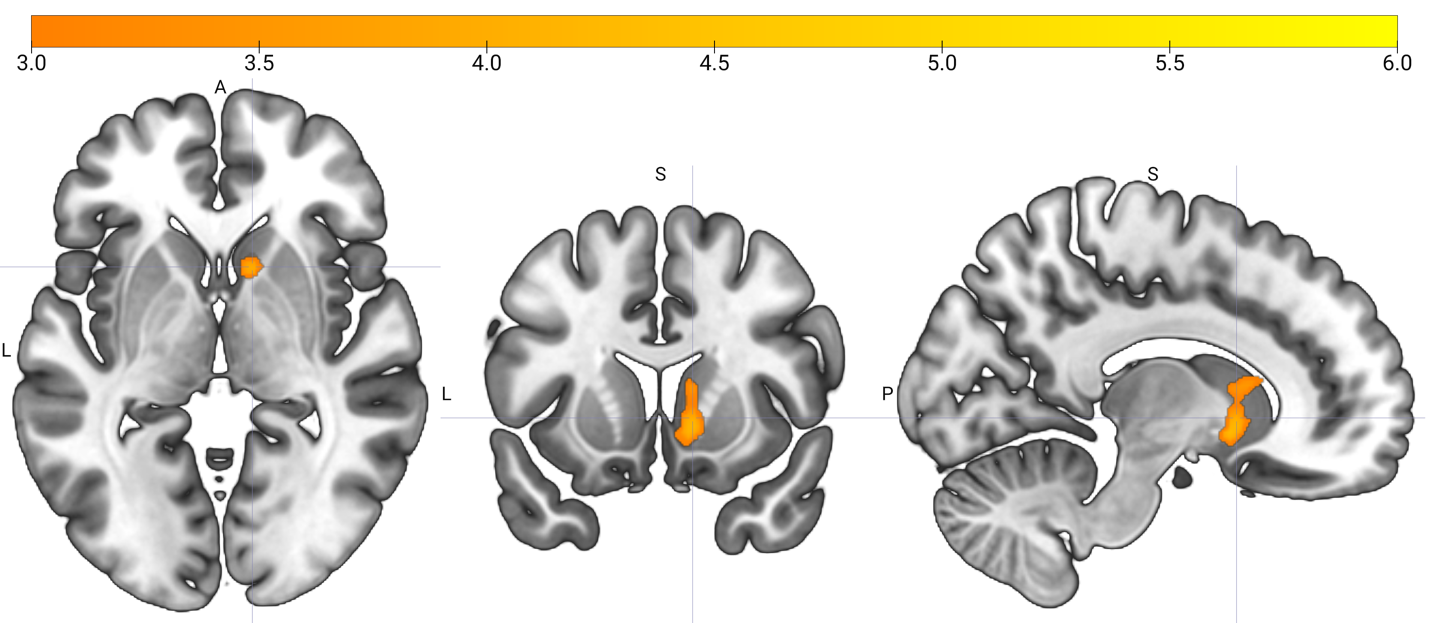
**

**Supplementary Figure 7.** Significant Clusters of Activation Differences in Bipolar Disorder Across Task-Based Experiments

**
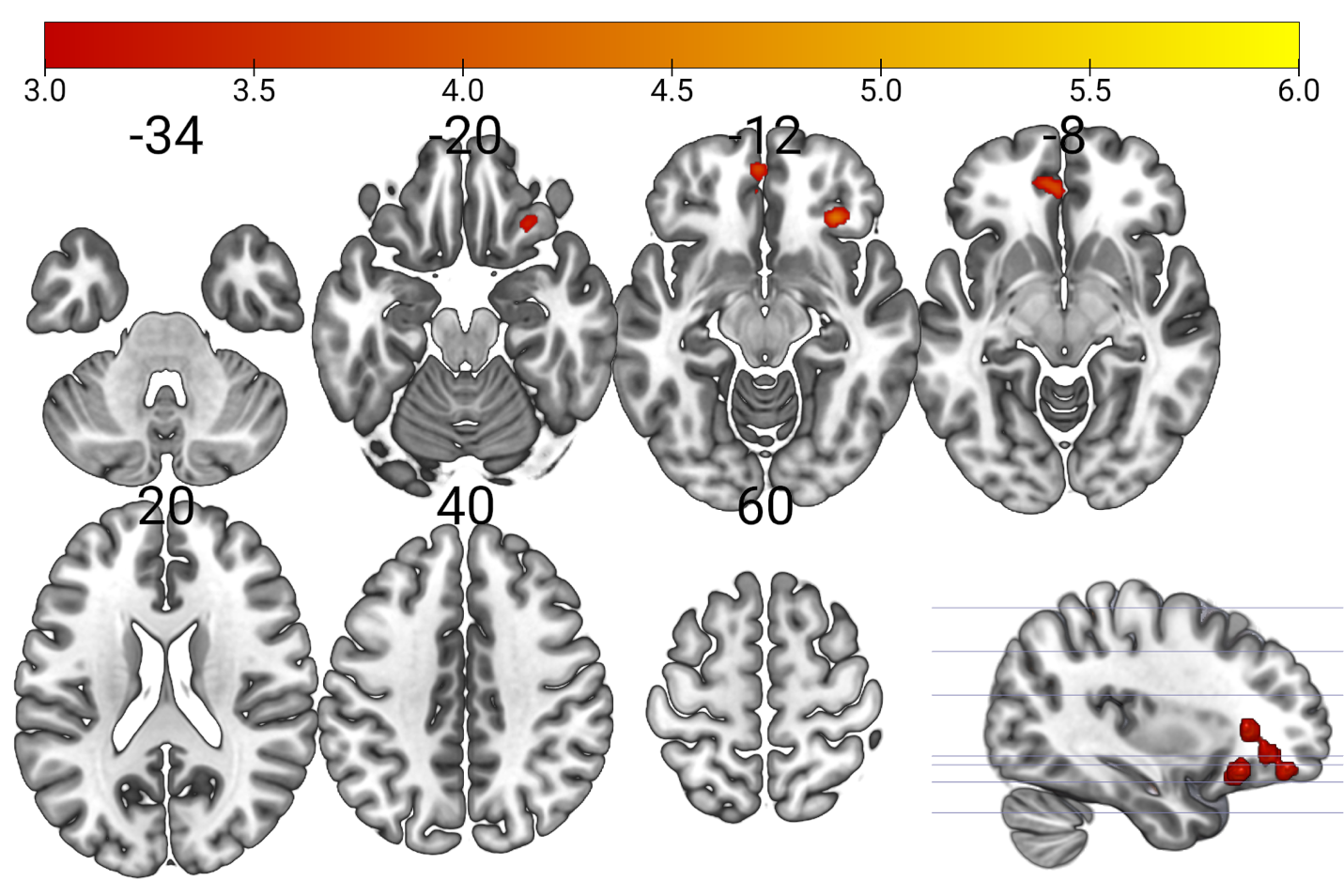
**

**Supplementary Figure 8.** Significant Clusters of Activation Differences in Bipolar Disorder Across Working Memory Paradigms

**
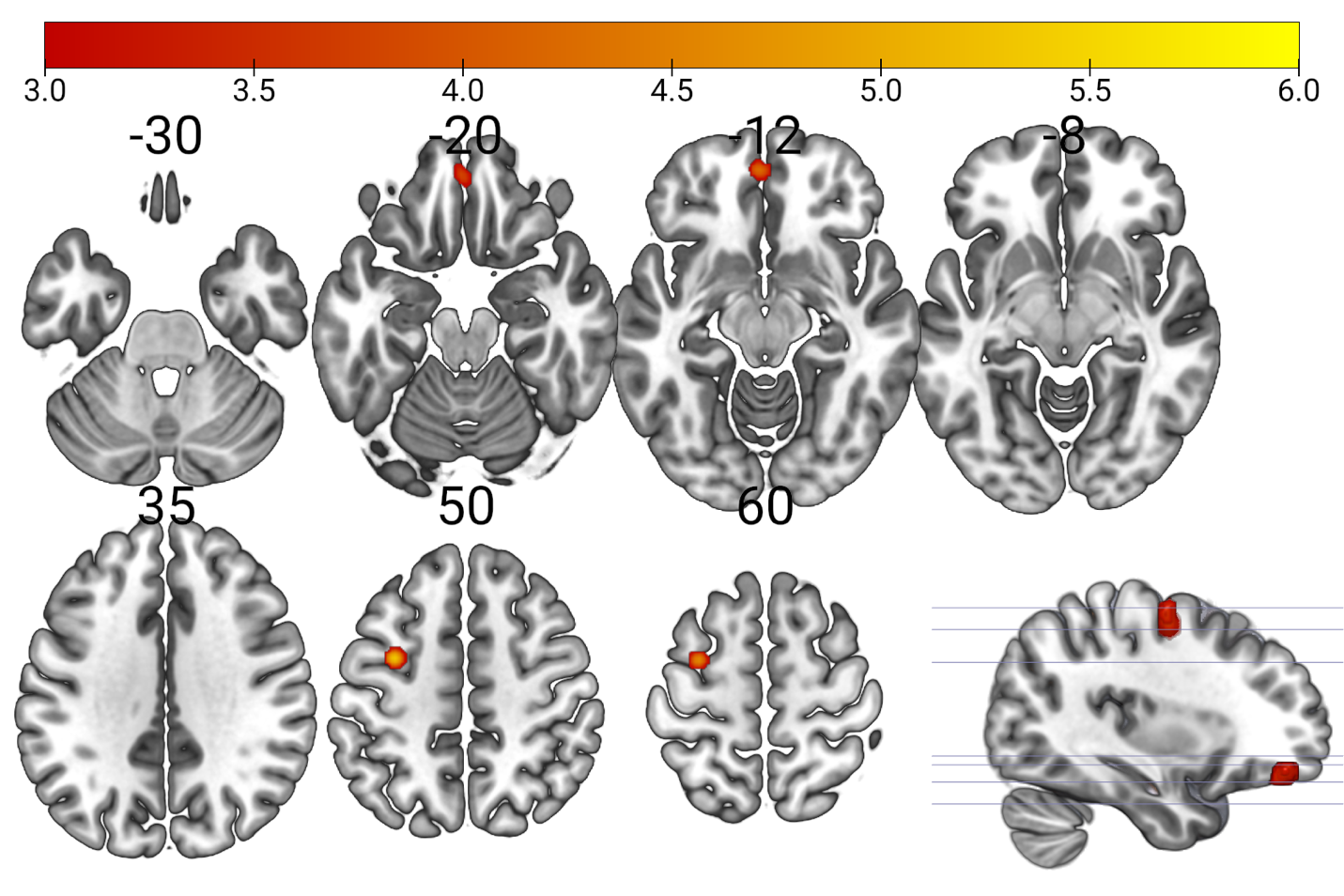
**

**Supplementary Table 6**. Significant Clusters from Exploratory Pooled ALE Meta-Analyses**.** BD, Bipolar Disorder; C, Controls; ext., Extended; MNI, Montreal Neuroimaging Institute. To avoid double counting, sample sizes (N) correspond to the total number of study participants, not the number of individual experiment participants. *No Convergence* indicates a null meta-analytic result i.e., no significant clusters were identified.

| **Method or Paradigm Type** | **N Foci** | **N BD** | **N Controls** | **Peak Coordinates (MNI)** | **Location** | **Cluster Size (*k* voxels)** | **Peak Intensity (*Z)*** |
| --- | --- | --- | --- | --- | --- | --- | --- |
| Task-Based Activation | 1975 | 3159 | 4455 | 32, 30, -14  4, 34, 4  *-2, 52, -14*  *-4, 44, -8*  *-8, 46, -8* | Ventrolateral Prefrontal Cortex  Dorsal Anterior Cingulate Cortex ext. to Medial Orbitofrontal Cortex | 134  216 | 5.02  4.78  *4.4*  *3.92*  *3.87* |
| Seed-Based Connectivity | 422 | 1083 | 1367 | – | *No Convergence* | – | – |
| Working Memory | 223 | 832 | 1066 | -32, -2, 52  -2, 52, -16 | Premotor/Supplementary Motor Cortex  Medial Orbitofrontal Cortex | 158  129 | 5.76  5.42 |
| Emotional Reactivity | 1280 | 1498 | 1779 | – | *No Convergence* | – | – |
| Emotion Regulation | 225 | 366 | 307 | – | *No Convergence* | – | – |

**Supplementary Table 7.** Studies Contributing to the Striatum Cluster from the Pooled Omnibus Meta-Analysis of 205 Studies

| **Anterior Caudate/Ventral Striatum: 10/10/-8, *k*=157, *z*=4.44** | |
| --- | --- |
| **Study ID** | **Contribution Score** |
| Altinay et al. 2016(34) | 2.39 |
| Argyelan et al. 2014(35) | 5.04 |
| Dutra et al. 2015(28) | 9 |
| Dutra et al. 2017(36) | 4.45 |
| Ellard et al. 2018(37) | 0.43 |
| Frangou 2012(38) | 6.63 |
| Hassel et al. 2008(39) | 1.86 |
| Lagopoulos & Malhi 2011(40) | 2.2 |
| Liu et al. 2012(41) | 4.15 |
| Lui et al. 2015(42) | 2.48 |
| Mah et al. 2007(43) | 2.53 |
| Malhi et al. 2005(44) | 2.67 |
| Malhi et al. 2007(45) | 4.56 |
| Man et al. 2019(46) | 1.41 |
| Manelis et al. 2016(47) | 5.12 |
| Marchand et al. 2007(48) | 3.13 |
| McIntosh et al. 2008(49) | 1.95 |
| Negoias et al. 2019(50) | 5.35 |
| Oertel-Knochel et al. 2013(51) | 1.56 |
| Pomarol-Clotet et al. 2015(52) | 6.55 |
| Pompei et al. 2011(53) | 4.8 |
| Redlich et al. 2015(54) | 3.87 |
| Shaffer et al. 2018(55) | 5.35 |
| Sharma et al. 2016(56) | 2.65 |
| Tecelão et al. 2019(57) | 4.31 |
| Townsend al. 2013(58) | 5.32 |
| Xu et al. 2014(59) | 0.48 |
| Zhang et al. 2016(60) | 0.41 |

**Supplementary Table 8.** Experiments Contributing to the Striatum Cluster from the Nested Omnibus Meta-Analysis of 506 Experiments

| **Anterior Caudate/Ventral Striatum: 12/10/-8, *k*=137, *z*=4.83** | | | | | | | |
| --- | --- | --- | --- | --- | --- | --- | --- |
| **Experiment ID** | **Contribution Score** | **Contrast/Direction** | **Mood State** | **Diagnostic Subtype** | **Medication Status** | **Psychosis** | **Control Group** |
| Altinay et al. 2016(34) | 2.66 | F_CBD R VS seed (BD>NC) | Not specified | BD I/II | Unmedicated | Partial with history | Non-Clinical |
| Argyelan et al. 2014(35) | 3.72 | C>BD | Not specified | Not reported | 100% medicated | Partial with history | Clinical: SZ |
| Chepenik et al. 2010(61) | 0.66 | BD>C | Not specified | Not reported | >75% medicated | Not reported | Non-Clinical |
| Dutra et al. 2015(28) | 8.92 | F_CBD reward receipt | Euthymic | BD I | 100% medicated | Not reported | Non-Clinical |
| Dutra et al. 2017(36) | 6.03 | F_CBD R VS seed | Euthymic | BD I | 100% medicated | Not reported | Non-Clinical |
| Dutra et al. 2017(36) | 6.03 | F_CBD L VS seed | Euthymic | BD I | 100% medicated | Not reported | Non-Clinical |
| Ellard et al. 2018(37) | 0.48 | C>BD R ventral anterior insula | Depressed | BD I/II | 100% medicated | No history | Non-Clinical |
| Foland-Ross et al. 2012(62) | 5.23 | C>BD | Euthymic | BD I | <75% medicated | Not reported | Non-Clinical |
| Frangou 2012(38) | 6.34 | F_CBD PPI | Euthymic | BD I | >75% medicated | Partial with history | Non-Clinical/ Clinical: NCR |
| Hassel et al. 2008(39) | 1.1 | F_CBD group x happy int. | Euthymic | Not reported | >75% medicated | No current psychosis | Non-Clinical |
| Lagopoulos & Malhi 2011(40) | 2.58 | BD>C Table 3 | Euthymic | BD I | <75% medicated | Not reported | Non-Clinical |
| Liu et al. 2012(41) | 3.05 | BD>C | Depressed | Not reported | 100% medicated | Not reported | Non-Clinical |
| Lui et al. 2015(42) | 1.22 | BD>C L pre/ postcentral gyrus | Not specified | BD I | 100% medicated | All with history | Non-Clinical |
| Mah et al. 2007(43) | 1.56 | BD>C | Depressed | BD II | 100% medicated | No current psychosis | Non-Clinical |
| Malhi et al. 2005(44) | 1.73 | F_CBD (NC>BD) | Euthymic | BD I | <75% medicated | Not reported | Non-Clinical |
| Malhi et al. 2007(45) | 3.07 | F_CBD positive affect (NC>BD) | Euthymic | BD I | <75% medicated | Not reported | Non-Clinical |
| **Anterior Caudate/Ventral Striatum: 12/10/-8, *k*=137, *z*=4.83** | | | | | | | |
| **Experiment ID** | **Contribution Score** | **Contrast/Direction** | **Mood State** | **Diagnostic Subtype** | **Medication Status** | **Psychosis** | **Control Group** |
| Man et al. 2019(46) | 1.83 | F_CBD L amygdala | Not specified | BD I | 100% medicated | Not reported | Clinical: MDD |
| Manelis et al. 2016(47) | 4.9 | C>BD guess | Depressed | BD I | 100% medicated | No current psychosis | Non-Clinical |
| Manelis et al. 2016(47) | 0.92 | BD>C wins | Depressed | BD I | 100% medicated | No current psychosis | Non-Clinical |
| Manelis et al. 2016(47) | 0.36 | BD>C losses | Depressed | BD I | 100% medicated | No current psychosis | Non-Clinical |
| Marchand et al. 2007(48) | 1.74 | BD>C | Depressed | BD I | >75% medicated | Partial with history | Non-Clinical |
| McIntosh et al. 2008(49) | 1.94 | F_CBD parametric (BD>NC) | Not specified | BD I | 100% medicated | All with current psychosis | Non-Clinical/ Clinical: SZ |
| Negoias et al. 2019(50) | 0.71 | BD>C Table 4 | Euthymic | BD I/II | Not reported | Not reported | Non-Clinical |
| Oertel-Knöchel et al. 2013(51) | 3.34 | C>BD retrieval | Euthymic | BD I | 100% medicated | Not reported | Non-Clinical |
| Pomarol-Clotet et al. 2015(52) | 1.12 | C>BD | Manic | BD I | 100% medicated | Partial with history | Non-Clinical |
| Pompei, Dima, et al. 2011(53) | 6.27 | F_CBD, PPI | Euthymic | BD I | >75% medicated | Partial with history | Non-Clinical/ Clinical: MDD, NCR |
| Redlich et al. 2015(54) | 3.62 | F_CBD (NC/MDD >BD) | Depressed | BD I | >75% medicated | Not reported | Non-Clinical/ Clinical: MDD |
| Rey et al. 2014(63) | 3.86 | C>BD fear>joy | Euthymic | NOS | 100% medicated | Not reported | Non-Clinical |
| Shaffer et al. 2018(55) | 2.38 | C>BD | Depressed | BD I | 100% medicated | Not reported | Non-Clinical |
| Sharma et al. 2016(56) | 3.37 | F_CBD (MDD>BD) | Depressed | BD I/II | 100% medicated | Not reported | Clinical: MDD |
| Townsend et al. 2013(58) | 3.53 | C>BD Table 2 | Euthymic | BD I | <75% medicated | Not reported | Non-Clinical |
| Xu et al. 2014(59) | 0.81 | BD>C | Not specified | Not reported | >75% medicated | Not reported | Non-Clinical |
| Zhang et al. 2019(64) | 0.47 | BD>C | Depressed | BD I | 100% medicated | Not reported | Non-Clinical |

Abbreviations: BD = Bipolar Disorder; C = Controls; DLPFC = Dorsolateral Prefrontal Cortex; F_CBD = Main effect of group or group x condition interaction; NC = Non-Clinical Controls; NCR = Non-Clinical Relatives; int. = interaction; L = Left; MDD = Major Depressive Disorder; NOS = Not Otherwise Specified; PPI = Psychophysiological Interaction; R = Right; SZ = Schizophrenia; VS = Ventral Striatum.

**Supplementary Table 9.** Studies Contributing to the Posterior Cingulate Cortex Cluster from the Pooled Resting-State Meta-Analysis of 63 Studies

| **Posterior Cingulate Cortex: 6/-52/32, *k*=144, *z=*4.47** | |
| --- | --- |
| **Study ID** | **Contribution Score** |
| Brady Jr. et al. 2017(65) | 15.28 |
| Ellard et al. 2018(37) | 6.17 |
| Gong et al. 2019(66) | 8.56 |
| Goya-Maldonado et al. 2016(67) | 12.16 |
| Liang et al. 2013(68) | 4.66 |
| Liu et al. 2013(69) | 6.47 |
| Wang et al. 2016(70) | 6.03 |
| Wang et al. 2017(71) | 1.36 |
| Wang et al. 2020(72) | 13.03 |
| Yang et al. 2019(73) | 10.28 |
| Zhong et al. 2019(74) | 14.91 |

**Supplementary Table 10.** Experiments Contributing to the Posterior Cingulate Cortex Cluster from the Nested Resting-State Meta-Analysis of 150 Experiments

| **Posterior Cingulate Cortex: 4/-52/34, *k*=183, *z*=4.78** | | | | | | | |
| --- | --- | --- | --- | --- | --- | --- | --- |
| **Experiment ID** | **Contribution Score** | **Contrast/Direction** | **Mood State** | **Diagnostic Subtype** | **Medication Status** | **Psychosis** | **Control Group** |
| Anticevic et al. 2014(75) | 6.39 | F_CBD MD midline thalamus (C>BD) | Euthymic | BD I | >75% medicated | Partial with history | Non-Clinical |
| Brady Jr. et al. 2017(65) | 11.85 | C>BD dorsal frontal ROI 38 | Euthymic | BD I | 100% medicated | All with history | Non-Clinical |
| Brady Jr. et al. 2017(65) | 9.32 | C>BD dorsal frontal ROI 61 | Euthymic | BD I | 100% medicated | All with history | Non-Clinical |
| Ellard et al. 2018(37) | 4.14 | BD>C L dorsal AI | Depressed | BD I/II | 100% medicated | No history | Non-Clinical |
| Ellard et al. 2018(37) | 5.96 | BD>C R dorsal AI | Depressed | BD I/II | 100% medicated | No history | Non-Clinical |
| Gong et al. 2019(66) | 5.98 | C>BD L PCC | Depressed | BD II | Unmedicated | Not reported | Non-Clinical |
| Goya-Maldonado et al. 2016(67) | 9.57 | F_CBD cingulo-opercular network (BD>MDD) | Depressed | BD I | >75% medicated | Not reported | Non-Clinical/ Clinical: MDD |
| Liang et al. 2013(68) | 3.55 | BD>C | Depressed | Not reported | Unmedicated | Not reported | Non-Clinical |
| Liu et al. 2013(69) | 4.23 | C>BD | Depressed | Not reported | >75% medicated | Not reported | Clinical: MDD |
| Qiu et al. 2018(76) | 0.49 | F_CBD (NC>BD) | Depressed | Not reported | >75% medicated | Not reported | Non-Clinical/ Clinical: MDD |
| Wang et al. 2016(70) | 5.13 | BD>C | Depressed | BD II | Unmedicated | Not reported | Non-Clinical |
| Wang et al. 2017(71) | 3.07 | F_CBD short-range FCS (NC>BD) | Depressed | BD II | Unmedicated | Not reported | Non-Clinical/ Clinical: MDD |
| Wang et al. 2020(72) | 11.04 | F_CBD posterior DMN (NC>BD) | Depressed | BD II | Unmedicated | Not reported | Non-Clinical/ Clinical: MDD |
| Yang et al. 2019(73) | 6.96 | C>BD | Not specified | Not reported | 100% medicated | Not reported | Non-Clinical |
| Zhong et al. 2019(74) | 11.05 | C>BD | Depressed | BD II | Unmedicated | Not reported | Non-Clinical |

Abbreviations: AI = Anterior Insula; BD = Bipolar Disorder; BPD = Borderline Personality Disorder; C = Controls; DMN = Default Mode Network; F_CBD = Main effect of group or group x condition interaction; FCS = Functional Connectivity Strength; NC = Non-Clinical Controls; L = Left; MD = Mediodorsal; MDD = Major Depressive Disorder; PCC = Posterior Cingulate Cortex; R = Right; ROI = Region of Interest; ROI = Region of Interest.

**Supplementary Table 11.** Experiments Contributing to the Posterior Cingulate Cortex Cluster from the Depressed Bipolar Disorder & Pooled Resting-State Meta-Analysis of 29 Studies

| **Posterior Cingulate Cortex: 6/-52/32, *k*=157, *z*=4.62** | |
| --- | --- |
| **Study ID** | **Contribution Score** |
| Ellard et al. 2018(37) | 13.43 |
| Gong et al. 2019(66) | 13.3 |
| Goya-Maldonado et al. 2016(67) | 20.47 |
| Liang et al. 2013(68) | 4.17 |
| Liu et al. 2013(69) | 5.15 |
| Wang et al. 2016(70) | 7.42 |
| Wang et al. 2017(71) | 1.14 |
| Wang et al. 2020(72) | 15.61 |
| Zhong et al. 2019(74) | 18.52 |

**Supplementary Table 12.** Experiments Contributing to the Posterior Cingulate Cortex Cluster from the Depressed Bipolar Disorder & Nested Resting-State Meta-Analysis of 62 Experiments

| **Posterior Cingulate Cortex: 8/-52/32, *k*=166, *z*=4.56** | | | | | | | |
| --- | --- | --- | --- | --- | --- | --- | --- |
| **Experiment ID** | **Contribution Score** | **Contrast/**  **Direction** | **Mood State** | **Diagnostic Subtype** | **Medication Status** | **Psychosis** | **Control Group** |
| Ellard et al. 2018(37) | 12.39 | BD>C L dorsal AI | Depressed | BD I/II | 100% medicated | No history | Non-Clinical |
| Ellard et al. 2018(37) | 12 | BD>C> R dorsal AI | Depressed | BD I/II | 100% medicated | No history | Non-Clinical |
| Gong et al. 2019(66) | 11.37 | C>BD L PCC | Depressed | BD II | Unmedicated | Not reported | Non-Clinical |
| Goya-Maldonado et al. 2016(67) | 19.65 | F_CBD cingulo-opercular network (BD>MDD) | Depressed | BD I | >75% medicated | Not reported | Non-Clinical/ Clinical: MDD |
| Liang et al. 2013(68) | 2.54 | BD>C | Depressed | Not reported | Unmedicated | Not reported | Non-Clinical |
| Liu et al. 2013(69) | 3.96 | C>BD | Depressed | Not reported | >75% medicated | Not reported | Clinical: MDD |
| Wang et al. 2016(70) | 6.3 | C>BD | Depressed | BD II | Unmedicated | Not reported | Non-Clinical |
| Wang et al. 2017(71) | 0.93 | F_CBD short-range FCS (NC>BD) | Depressed | BD II | Unmedicated | Not reported | Non-Clinical/ Clinical: MDD |
| Wang et al. 2020(72) | 14.09 | F_CBD posterior DMN (NC>BD) | Depressed | BD II | Unmedicated | Not reported | Non-Clinical/ Clinical: MDD |
| Zhong et al. 2019(74) | 15.97 | C>BD | Depressed | BD II | Unmedicated | Not reported | Non-Clinical |

Abbreviations: AI = Anterior Insula; BD = Bipolar Disorder; C = Controls; F_CBD = Main effect of group or group x condition interaction; NC = Non-Clinical Controls; L = Left; MDD = Major Depressive Disorder; PCC = Posterior Cingulate Cortex; R = Right.

**Supplementary Table 13.** Studies Contributing to the Inferior Parietal Lobule, Superior Parietal Lobule, Orbitofrontal Cortex, and Striatum Clusters from the Pooled Cognitive Tasks Meta-Analysis of 69 Studies

| **Orbitofrontal Cortex: -2/52/-14, *k*=115, *z*=5.27** | |
| --- | --- |
| **Study ID** | **Contribution Score** |
| Alonso-Lana et al. 2016(77) | 14.23 |
| Benson et al. 2014(78) | 8.2 |
| Goikolea et al. 2019(79) | 16.26 |
| Liberg et al. 2013(80) | 8.15 |
| Pomarol-Clotet et al. 2015(52) | 4.9 |
| Robinson et al. 2009(81) | 14.44 |
| Rodríguez-Cano et al. 2017(82) | 17.01 |
| Townsend et al. 2013(83) | 15.78 |
| **Inferior Parietal Lobule: 38/-44/46, *k*=154, *z*=5.21** | |
| **Study ID** | **Contribution Score** |
| Benson et al. 2008(84) | 2.3 |
| Brown et al. 2012(85) | 1.89 |
| Frangou 2012(38) | 24.43 |
| Jamadar et al. 2013(86) | 11.56 |
| Marchand et al. 2011(87) | 0.7 |
| Pompei et al. 2011(88) | 24.07 |
| Reavis et al. 2017(89) | 7.41 |
| Robinson et al. 2009(81) | 14.09 |
| Taylor Tavares et al. 2008(90) | 12.46 |
| **Superior Parietal Lobule: -20/-70/52, *k*=121, *z*=5.87** | |
| **Study ID** | **Contribution Score** |
| Brooks III et al. 2015(91) | 10.53 |
| Drapier et al. 2008(92) | 14.9 |
| Frangou 2012(38) | 18.85 |
| Glahn et al. 2010(93) | 12.62 |
| Liberg et al. 2013(94) | 8.25 |
| Monks et al. 2004(95) | 14.46 |
| Pompei et al. 2011(88) | 18.62 |
| Townsend et al. 2013(83) | 0.33 |
| **Striatum: -10/8/-2, *k*=144, *z*=4.53** | |
| **Study ID** | **Contribution Score** |
| Benson et al. 2008(84) | 2.37 |
| Fleck et al. 2011(96) | 0.33 |
| **Striatum: -10/8/-2, *k*=144, *z*=4.53** | |
| **Study ID** | **Contribution Score** |
| Frangou 2012(38) | 28.27 |
| Joshi et al. 2016(97) | 4.94 |
| Marchand et al. 2007(48) | 8.21 |
| Palaniyappan & Liddle 2014(98) | 6.63 |
| Pomarol-Clotet et al. 2015(52) | 2.2 |
| Pompei et al. 2011(54) | 21.29 |
| Pompei et al. 2011(88) | 8.58 |
| Shaffer et al. 2018(55) | 9.36 |
| Sugihara et al. 2017(99) | 5.16 |
| Townsend et al. 2013(83) | 1.43 |

**Supplementary Table 14.** Experiments Contributing to the Inferior Parietal Lobule, Superior Parietal Lobule, and Orbitofrontal Cortex Clusters from the Nested Cognitive Tasks Meta-Analysis of 134 Experiments

| **Orbitofrontal Cortex: -2/52/-14, *k*=120, *z*=5.10** | | | | | | | |
| --- | --- | --- | --- | --- | --- | --- | --- |
| **Experiment ID** | **Contribution Score** | **Contrast/**  **Direction** | **Mood State** | **Diagnostic Subtype** | **Medication Status** | **Psychosis** | **Control Group** |
| Alonso-Lana et al. 2016(77) | 12.78 | F_CBD (BD>NC/NCR) | Euthymic | BD I/II | 100% medicated | Not reported | Non-Clinical/Clinical: NCR |
| Benson et al. 2014(78) | 4.71 | F_CBD L amygdala (BD>NC) | Depressed | BD I/II | Unmedicated | Not reported | Non-Clinical |
| Benson et al. 2014(78) | 10.9 | F_CBD R amygdala (BD>NC) | Depressed | BD I/II | Unmedicated | Not reported | Non-Clinical |
| Goikolea et al. 2019(79) | 14.82 | BD>C 2-back>BL | Manic | Not reported | 100% medicated | Partial with current psychosis | Non-Clinical |
| Liberg et al. 2013(80) | 7.31 | BD>C | Depressed | BD I | 100% medicated | Not reported | Non-Clinical |
| Pomarol-Clotet et al. 2015(52) | 4.16 | BD>C | Depressed | BD I/II | 100% medicated | Partial with history | Non-Clinical |
| Robinson et al. 2009(81) | 13.2 | BD>C novelty | Euthymic | BD I | >75% medicated | Not reported | Non-Clinical |
| Rodríguez-Cano et al. 2017(82) | 15.21 | BD>C 2-back>BL | Depressed | BD I | 100% medicated | Partial with history | Non-Clinical |
| Townsend et al. 2013(83) | 15.71 | F_CBD | Euthymic | BD I | <75% medicated | Not reported | Non-Clinical/Clinical: ADHD |
| **Inferior Parietal Lobule: 38/-44/46, *k*=139, *z*=5.03** | | | | | | | |
| **Experiment ID** | **Contribution Score** | **Contrast/**  **Direction** | **Mood State** | **Diagnostic Subtype** | **Medication Status** | **Psychosis** | **Control Group** |
| Benson et al. 2008(84) | 2.1 | F_CBD L inferior parietal (BD>C) | Depressed | BD I/II | Unmedicated | Not reported | Non-Clinical |
| Brown et al. 2012(85) | 1.97 | C>BD | Not specified | BD I | <75% medicated | Not reported | Non-Clinical |
| Frangou 2012(38) | 24.8 | F_CBD main effect of group (NC/NCR>BD) | Euthymic | BD I | >75% medicated | Partial with history | Non-Clinical/ Clinical: NCR |
| **Inferior Parietal Lobule: 38/-44/46, *k*=139, *z*=5.03** | | | | | | | |
| **Experiment ID** | **Contribution Score** | **Contrast/**  **Direction** | **Mood State** | **Diagnostic Subtype** | **Medication Status** | **Psychosis** | **Control Group** |
| Jamadar et al. 2013(86) | 11.44 | F_CBD (NC>BD) | Not specified | NOS | <75% medicated | Partial with current psychosis | Non-Clinical/ Clinical: SZ |
| Marchand et al. 2011(87) | 0.66 | BD>C | Depressed | BD II | Unmedicated | Not reported | Non-Clinical |
| Pompei, Jogia, et al. 2011(88) | 24.42 | F_CBD (all C>BD) | Euthymic | BD I | >75% medicated | Partial with history | Non-Clinical/ Clinical: NCR, MDD Relatives |
| Reavis et al. 2017(89) | 7.22 | C>BD | Not specified | Not reported | 100% medicated | Not reported | Non-Clinical |
| Robinson et al. 2009(81) | 14 | C>BD novelty | Euthymic | BD I | >75% medicated | Not reported | Non-Clinical |
| Taylor Tavares et al. 2008(90) | 12.15 | BD>C error switches | Depressed | Not reported | Unmedicated | Not reported | Clinical: MDD |
| **Superior Parietal Lobule: -20/-70/52, *k*=139, *z*=6.27** | | | | | | | |
| **Experiment ID** | **Contribution Score** | **Contrast/**  **Direction** | **Mood State** | **Diagnostic Subtype** | **Medication Status** | **Psychosis** | **Control Group** |
| Brooks III et al. 2015(91) | 9.17 | C>BD | Depressed | BD II | Unmedicated | Not reported | Non-Clinical |
| Drapier et al. 2008(92) | 13.52 | BD>C 1-back | Euthymic | BD I | >75% medicated | All with history | Non-Clinical |
| Drapier et al. 2008(92) | 13.52 | BD>C 2-back | Euthymic | BD I | >75% medicated | All with history | Non-Clinical |
| Frangou 2012(38) | 15.96 | F_CBD main effect of group (NC/NCR>BD) | Euthymic | BD I | >75% medicated | Partial with history | Non-Clinical/ Clinical: NCR |
| Glahn et al. 2010(93) | 10.66 | C>BD encoding | Euthymic | BD I | >75% medicated | Not reported | Non-Clinical |
| Liberg et al. 2013(94) | 7.19 | C>BD | Depressed | BD I | 100% medicated | Not reported | Non-Clinical |
| Monks et al. 2004(95) | 12.15 | F_CBD (NC>BD) | Euthymic | BD I | 100% medicated | Not reported | Non-Clinical |
| Pompei, Jogia, et al. 2011(88) | 15.78 | F_CBD (all C>BD) | Euthymic | BD I | >75% medicated | Partial with history | Non-Clinical/ Clinical: NCR, MDD Relatives |
| Yoshimura et al. 2014(100) | 0.51 | BD>C | Depressed | BD I | 100% medicated | Not reported | Non-Clinical |

Abbreviations: ADHD = Attention Deficit Hyperactivity Disorder; ALE = Activation Likelihood Estimation; BD = Bipolar Disorder; BL = Baseline; C = Controls; F_CBD = Main effect of group or group x condition interaction; NC = Non-Clinical Controls; NCR = Non-Clinical Relatives; L = Left; MDD = Major Depressive Disorder; R = Right; SZ = Schizophrenia.

**Supplementary Table 15.** Studies Contributing to the Orbitofrontal Cortex and Ventral Anterior Cingulate Cortex Clusters from the Pooled Cognitive Tasks Meta-Analysis of Bipolar Disorder Hyperactivation Alone (27 Studies)

| **Orbitofrontal Cortex: -4/52/-16, *k*=156, *z*=5.56** | |
| --- | --- |
| **Study ID** | **Contribution Score** |
| Fernández-Corcuera et al. 2013(101) | 0.35 |
| Goikolea et al. 2019(79) | 23.56 |
| Liberg et al. 2013(80) | 10.18 |
| Pomarol-Clotet et al. 2015(52) | 16.9 |
| Robinson et al. 2009(81) | 20.81 |
| Rodríguez-Cano et al. 2017(82) | 27.57 |
| **Ventral Anterior Cingulate Cortex: -4/36/-14, *k*=220, *z*=4.6** | |
| **Study ID** | **Contribution Score** |
| Alonso-Lana et al. 2016(102) | 17.03 |
| Alonso-Lana et al. 2019(103) | 16.97 |
| Fernández-Corcuera et al. 2013(101) | 23.32 |
| Goikolea et al. 2019(79) | 5.61 |
| Pomarol-Clotet et al. 2012(104) | 14.65 |
| Pomarol-Clotet et al. 2015(52) | 0.31 |
| Reavis et al. 2017(89) | 0.48 |
| Robinson et al. 2009(81) | 21.18 |

**Supplementary Table 16.** Experiments Contributing to the Ventral Anterior Cingulate Cortex and Orbitofrontal Cortex Clusters from the Nested Cognitive Tasks Meta-Analysis of Bipolar Disorder Hyperactivation Alone (40 Experiments)

| **Orbitofrontal Cortex: -4/52/-16, *k*=174, *z*=5.42** | | | | | | | |
| --- | --- | --- | --- | --- | --- | --- | --- |
| **Experiment ID** | **Contribution Score** | **Contrast/**  **Direction** | **Mood State** | **Diagnostic Subtype** | **Medication Status** | **Psychosis** | **Control Group** |
| Fernández-Corcuera et al. 2013(101) | 0.3 | BD>C 2-back>BL | Depressed | Not reported | 100% medicated | All with current psychosis | Non-Clinical |
| Fernández-Corcuera et al. 2013(101) | 0.16 | BD>C increasing WM load | Depressed | Not reported | 100% medicated | All with current psychosis | Non-Clinical |
| Goikolea et al. 2019(79) | 20.74 | BD>C 2-back>BL | Manic | Not reported | 100% medicated | Partial with current psychosis | Non-Clinical |
| Liberg et al. 2013(80) | 8.07 | BD>C | Depressed | BD I | 100% medicated | Not reported | Non-Clinical |
| Pomarol-Clotet et al. 2015(52) | 8.3 | BD>C | Manic | BD I | 100% medicated | Partial with history | Non-Clinical |
| Pomarol-Clotet et al. 2015(52) | 19.46 | BD>C | Depressed | BD I/II | 100% medicated | Partial with history | Non-Clinical |
| Robinson et al. 2009(81) | 17.97 | BD>C novelty | Euthymic | BD I | >75% medicated | Not reported | Non-Clinical |
| Rodríguez-Cano et al. 2017(82) | 24.41 | BD>C 2-back>BL | Depressed | BD I | 100% medicated | Partial with history | Non-Clinical |
| **Ventral Anterior Cingulate Cortex: -4/36/-12, *k*=259, *z*=5.41** | | | | | | | |
| **Experiment ID** | **Contribution Score** | **Contrast/**  **Direction** | **Mood State** | **Diagnostic Subtype** | **Medication Status** | **Psychosis** | **Control Group** |
| Alonso-Lana et al. 2016(102) | 6.95 | BD>C 2-back>BL | Euthymic | BD I | 100% medicated | Not reported | Non-Clinical |
| Alonso-Lana et al. 2016(102) | 8.5 | BD>C 2-back>1-back | Euthymic | BD I | 100% medicated | Not reported | Non-Clinical |
| Alonso-Lana et al. 2019(103) | 14.29 | BD>C | Manic | BD I | 100% medicated | Not reported | Non-Clinical |
| Fernández-Corcuera et al. 2013(101) | 17.68 | BD>C 2-back>BL | Depressed | Not reported | 100% medicated | All with current psychosis | Non-Clinical |
| **Ventral Anterior Cingulate Cortex: -4/36/-12, *k*=259, *z*=5.41** | | | | | | | |
| **Experiment ID** | **Contribution Score** | **Contrast/**  **Direction** | **Mood State** | **Diagnostic Subtype** | **Medication Status** | **Psychosis** | **Control Group** |
| Fernández-Corcuera et al. 2013(101) | 17.44 | BD>C increasing WM load | Depressed | Not reported | 100% medicated | All with current psychosis | Non-Clinical |
| Goikolea et al. 2019(79) | 4.2 | BD>C 2-back>BL | Manic | Not reported | 100% medicated | Partial with current psychosis | Non-Clinical |
| Pomarol-Clotet et al. 2012(104) | 12.72 | BD>C | Manic | Not reported | Unmedicated | All with current psychosis | Non-Clinical |
| Pomarol-Clotet et al. 2015(52) | 0.47 | BD>C | Depressed | BD I/II | 100% medicated | Partial with history | Non-Clinical |
| Reavis et al. 2017(89) | 0.45 | BD>C | Not specified | Not reported | 100% medicated | Not reported | Clinical: SZ |
| Robinson et al. 2009(81) | 16.61 | BD>C novelty | Euthymic | BD I | >75% medicated | Not reported | Non-Clinical |
| Rodríguez-Cano et al. 2017(82) | 0.23 | BD>C 2-back>BL | Depressed | BD I | 100% medicated | Partial with history | Non-Clinical |

Abbreviations: BD = Bipolar Disorder; BL = Baseline; C = Controls; SZ = Schizophrenia; WM = Working Memory.

**Supplementary Table 17.** Studies Contributing to the Premotor Cortex Cluster from the Pooled Cognitive Tasks Meta-Analysis of Bipolar Disorder Hypoactivation Alone (29 Studies)

| **Premotor Cortex: -32/-2/52, *k*=121, *z*=5.47** | |
| --- | --- |
| **Study ID** | **Contribution Score** |
| Alonso-Lana et al. 2019(103) | 21.32 |
| Fernández-Corcuera et al. 2013(101) | 20.6 |
| Pomarol-Clotet et al. 2012(104) | 25.42 |
| Pomarol-Clotet et al. 2015(52) | 32.13 |

**Supplementary Table 18.** Experiments Contributing to the Premotor Cortex Cluster from the Nested Cognitive Tasks Meta-Analysis of Bipolar Disorder Hypoactivation Alone (43 Experiments)

| **Premotor Cortex: -32/-2/52, *k*=154, *z*=6.31** | | | | | | | |
| --- | --- | --- | --- | --- | --- | --- | --- |
| **Experiment ID** | **Contribution Score** | **Contrast/**  **Direction** | **Mood State** | **Diagnostic Subtype** | **Medication Status** | **Psychosis** | **Control Group** |
| Alonso-Lana et al. 2019(103) | 15.55 | C>BD | Manic | BD I | 100% medicated | Not reported | Non-Clinical |
| Fernández-Corcuera et al. 2013(101) | 18.35 | C>BD 2-back>BL | Depressed | Not reported | 100% medicated | All with current psychosis | Non-Clinical |
| Fernández-Corcuera et al. 2013(101) | 18.35 | C>BD increasing WM load | Depressed | Not reported | 100% medicated | All with current psychosis | Non-Clinical |
| Pomarol-Clotet et al. 2012(104) | 19.35 | C>BD | Manic | Not reported | Unmedicated | All with current psychosis | Non-Clinical |
| Pomarol-Clotet et al. 2015(52) | 9.54 | C>BD | Manic | BD I | 100% medicated | Partial with history | Non-Clinical |
| Pomarol-Clotet et al. 2015(52) | 18.17 | C>BD | Depressed | BD I/II | 100% medicated | Partial with history | Non-Clinical |

Abbreviations: BD = Bipolar Disorder; BL = Baseline; C = Controls; WM = Working Memory.

**Supplementary Table 19.** Studies Contributing to the Superior Parietal Lobule Cluster from the Euthymic Bipolar Disorder & Pooled Cognitive Tasks Meta-Analysis of 31 Studies

| **Superior Parietal Lobule: -20/-70/52, *k*=121, *z*=6.21** | |
| --- | --- |
| **Study ID** | **Contribution Score** |
| Drapier et al. 2008(92) | 19.43 |
| Frangou 2012(38) | 24 |
| Glahn et al. 2010(93) | 14.21 |
| Monks et al. 2004(95) | 17.56 |
| Pompei et al. 2011(88) | 23.72 |
| Townsend et al. 2013(83) | 0.37 |

**Supplementary Table 20.** Experiments Contributing to the Superior Parietal Lobule Cluster from the Euthymic Bipolar Disorder & Nested Cognitive Tasks Meta-Analysis of 55 Experiments

| **Superior Parietal Lobule: -20/-70/50, *k*=150, *z*=6.78** | | | | | | | |
| --- | --- | --- | --- | --- | --- | --- | --- |
| **Experiment ID** | **Contribution Score** | **Contrast/**  **Direction** | **Mood State** | **Diagnostic Subtype** | **Medication Status** | **Psychosis** | **Control Group** |
| Drapier et al. 2008(92) | 16.96 | BD>C 1-back | Euthymic | BD I | >75% medicated | All with history | Non-Clinical |
| Drapier et al. 2008(92) | 16.96 | BD>C 2-back | Euthymic | BD I | >75% medicated | All with history | Non-Clinical |
| Frangou 2012(38) | 18.95 | F_CBD main effect of group (NC/NCR>BD) | Euthymic | BD I | >75% medicated | Partial with history | Non-Clinical/Clinical: NCR |
| Glahn et al. 2010(93) | 12.83 | C>BD encoding | Euthymic | BD I | >75% medicated | Not reported | Non-Clinical |
| Monks et al. 2004(95) | 14.34 | F_CBD (C>BD) | Euthymic | BD I | 100% medicated | Not reported | Non-Clinical |
| Pompei et al. 2011(88) | 18.76 | F_CBD (all C>BD) | Euthymic | BD I | >75% medicated | Partial with history | Non-Clinical/Clinical: MDD, NCR |
| Townsend et al. 2013(83) | 0.36 | F_CBD | Euthymic | BD I | <75% medicated | Not reported | Non-Clinical/Clinical: ADHD |

Abbreviations: ADHD = Attention Deficit Hyperactivity Disorder; BD = Bipolar Disorder; C = Controls; F_CBD = Main effect of group or group x condition interaction; NC = Non-Clinical Controls; NCR = Non-Clinical Relatives; MDD = Major Depressive Disorder.

**Supplementary Table 21.** Studies Contributing to the Premotor Cortex Cluster from the Hypo/manic Bipolar Disorder & Pooled Cognitive Tasks Meta-Analysis of 7 Studies

| **Premotor Cortex: -32/-2/58, *k*=118, *z*=5.62** | |
| --- | --- |
| **Study ID** | **Contribution Score** |
| Alonso-Lana et al. 2019(103) | 37.01 |
| Pomarol-Clotet et al. 2012(104) | 31.48 |
| Pomarol-Clotet et al. 2015(52) | 31.21 |

**Supplementary Table 22.** Experiments Contributing to the Premotor Cortex Cluster from the Hypo/manic Bipolar Disorder & Nested Cognitive Tasks Meta-Analysis of 12 Experiments

| **Premotor/Supplementary Motor Cortex: -32/-2/58, *k*=118, *z*=5.54** | | | | | | | |
| --- | --- | --- | --- | --- | --- | --- | --- |
| **Experiment ID** | **Contribution Score** | **Contrast/**  **Direction** | **Mood State** | **Diagnostic Subtype** | **Medication Status** | **Psychosis** | **Control Group** |
| Alonso-Lana et al. 2019(103) | 37.01 | C>BD | Manic | BD I | 100% medicated | Not reported | Non-Clinical |
| Pomarol-Clotet et al. 2012(104) | 31.48 | C>BD | Manic | Not reported | Unmedicated | All with current psychosis | Non-Clinical |
| Pomarol-Clotet et al. 2015(52) | 31.21 | C>BD | Manic | BD I | 100% medicated | Partial with history | Non-Clinical |

Abbreviations: BD = Bipolar Disorder; C = Controls.

**Supplementary Table 23.** Studies Contributing to the Amygdala Cluster from the Pooled Emotional Task Meta-Analysis of 77 Studies

| **Amygdala: -26/-6/-20, *k*=128, *z*=4.25** | |
| --- | --- |
| **Study ID** | **Contribution Score** |
| Chen et al. 2006(105) | 6.78 |
| Dutra et al. 2017(36) | 5.45 |
| Foland et al. 2008(106) | 6.22 |
| Korgaonkar et al. 2019(107) | 10.38 |
| Lagopoulos & Malhi 2007(108) | 0.52 |
| Malhi et al. 2005(44) | 10.31 |
| Malhi et al. 2007(109) | 1.06 |
| Man et al. 2019(46) | 3.12 |
| Manelis et al. 2016(47) | 1.76 |
| Mitchell et al. 2004(110) | 0.75 |
| Negoias et al. 2019(50) | 3.72 |
| Perlman et al. 2012(111) | 7.64 |
| Rey et al. 2014(63) | 4.19 |
| Vai et al. 2015(112) | 3.8 |
| Vizueta et al. 2012(113) | 14.4 |
| Wang et al. 2009(114) | 6.76 |
| Yip et al. 2015(115) | 1.17 |
| Young et al. 2016(116) | 10 |

**Supplementary Table 24.** Experiments Contributing to the Amygdala Cluster from the Nested Emotional Task Meta-Analysis of 222 Experiments

| **Amygdala: -28/-4/-20, *k*=189, *z*=4.47** | | | | | | | |
| --- | --- | --- | --- | --- | --- | --- | --- |
| **Experiment ID** | **Contribution Score** | **Contrast/**  **Direction** | **Mood State** | **Diagnostic Subtype** | **Medication Status** | **Psychosis** | **Control Group** |
| Chen et al. 2006(105) | 4.75 | F_CBD sad face x group (BDm>C) | Not specified | BD I | 100% medicated | Partial with current psychosis | Non-Clinical |
| Dutra et al. 2017(36) | 5.75 | F_CBD R VS seed (BD>C) | Euthymic | BD I | 100% medicated | Not reported | Non-Clinical |
| Dutra et al. 2017(36) | 5.3 | F_CBD L VS seed (BD>C) | Euthymic | BD I | 100% medicated | Not reported | Non-Clinical |
| Foland et al. 2008(106) | 5.17 | BD>C perceive emotion > control | Manic | BD I | >75% medicated | Not reported | Non-Clinical |
| Foland et al. 2008(106) | 4.64 | BD>C label emotion > control | Manic | BD I | >75% medicated | Not reported | Non-Clinical |
| Korgaonkar et al. 2019(107) | 8.27 | F_CBD subliminal emotion processing (MDD>BD) | Euthymic | BD I | 100% medicated | Not reported | Clinical: MDD |
| Lagopoulos & Malhi 2007(108) | 0.33 | BD>C | Euthymic | BD I | <75% medicated | Not reported | Non-Clinical |
| Lennox et al. 2004(117) | 0.36 | C>BD | Manic | BD I | 100% medicated | Not reported | Non-Clinical |
| Malhi et al. 2005(44) | 7.28 | F_CBD (C>BD) | Euthymic | BD I | <75% medicated | Not reported | Non-Clinical |
| Malhi et al. 2007(109) | 0.86 | F_CBD (BD>C) | Euthymic | BD I | <75% medicated | Not reported | Non-Clinical |
| Man et al. 2019(46) | 2.54 | F_CBD L amygdala | Not specified | BD I | 100% medicated | Not reported | Clinical: MDD |
| Manelis et al. 2016(47) | 0.84 | C>BD feedback | Depressed | BD I | 100% medicated | No current psychosis | Non-Clinical |
| Manelis et al. 2016(47) | 0.33 | BD>C losses | Depressed | BD I | 100% medicated | No current psychosis | Clinical: MDD |
| Mitchell et al. 2004(110) | 0.97 | C>BD pure | Not specified | Not reported | 100% medicated | Not reported | Non-Clinical |
| **Amygdala: -28/-4/-20, *k*=189, *z*=4.47** | | | | | | | |
| **Experiment ID** | **Contribution Score** | **Contrast/**  **Direction** | **Mood State** | **Diagnostic Subtype** | **Medication Status** | **Psychosis** | **Control Group** |
| Mitchell et al. 2004(110) | 0.33 | BD>C unfiltered | Not specified | Not reported | 100% medicated | Not reported | Non-Clinical |
| Morris et al. 2012(118) | 0.38 | BD>C downregulation | Not specified | BD I | 100% medicated | All with current psychosis | Clinical: SZ |
| Morris et al. 2012(118) | 0.51 | BD>C downregulation | Not specified | BD I | 100% medicated | All with current psychosis | Clinical: SZ |
| Negoias et al. 2019(50) | 3.6 | BD>C (Table 3) | Euthymic | BD I/II | Not reported | Not reported | Non-Clinical |
| Negoias et al. 2019(50) | 0.88 | BD>C (Table 4) | Euthymic | BD I/II | Not reported | Not reported | Non-Clinical |
| Negoias et al. 2019(50) | 1.29 | BD>C (Table 5) | Euthymic | BD I/II | Not reported | Not reported | Non-Clinical |
| Perlman et al. 2012(111) | 5.16 | F_CBD (BD>C) | Euthymic | BD I | 100% medicated | No current psychosis | Non-Clinical |
| Rey et al. 2014(63) | 2.73 | C>BD group x load x congruence | Depressed | NOS | 100% medicated | Not reported | Non-Clinical |
| Vai et al. 2015(112) | 2.2 | C>BD | Depressed | BD I | 100% medicated | No current psychosis | Non-Clinical |
| Vizueta et al. 2012(113) | 9.28 | BD>C R amygdala PPI | Depressed | BD II | Unmedicated | Not reported | Non-Clinical |
| Wang et al. 2009(114) | 6.2 | C>BD fear | Not specified | Not reported | >75% medicated | Not reported | Non-Clinical |
| Wang et al. 2009(114) | 6.2 | C>BD happy | Not specified | Not reported | >75% medicated | Not reported | Non-Clinical |
| Yip et al. 2015(115) | 0.66 | C>BD 2^nd^ anticipation phase: losses> neutral | Euthymic | NOS | Unmedicated | Not reported | Non-Clinical |
| Young et al. 2016(116) | 9.21 | F_CBD positive memories (BD>NC/MDD) | Depressed | BD I | Unmedicated | Not reported | Non-Clinical/Clinical: MDD |
| Young et al. 2016(116) | 1.98 | F_CBD negative memories (BD>NC/ MDD>BD) | Depressed | BD I | Unmedicated | Not reported | Non-Clinical/Clinical: MDD |

Abbreviations: BD = Bipolar Disorder; BDm = Bipolar Disorder Mania; C = Controls; F_CBD = Main effect of group or group x condition interaction; NCR = Non-Clinical Relatives; L = Left; MDD = Major Depressive Disorder; NOS = Not Otherwise Specified; PPI = Psychophysiological Interaction; R = Right; SZ = Schizophrenia; VS = Ventral Striatum.

**Supplementary Table 25.** Studies Contributing to the Ventrolateral Prefrontal Cortex Cluster from the Pooled Emotional Tasks Meta-Analysis of Bipolar Disorder Hypoactivation Alone (36 Studies)

| **Ventrolateral Prefrontal Cortex: 32/30/-14, *k*=125, *z*=5.46** | |
| --- | --- |
| **Study ID** | **Contribution Score** |
| Favre et al. 2013(119) | 9.44 |
| Foland et al. 2008(106) | 10.31 |
| Foland-Ross et al. 2012(62) | 18.51 |
| Jogia et al. 2008(120) | 11.63 |
| Killgore et al. 2008(121) | 2.52 |
| Manelis et al. 2016(47) | 5.42 |
| Rey et al. 2014(63) | 0.72 |
| Townsend et al. 2013(58) | 4.13 |
| Trost et al. 2014(122) | 17.31 |
| Yip et al. 2015(115) | 18.84 |

**Supplementary Table 26.** Experiments Contributing to the Ventrolateral Prefrontal Cortex Cluster from the Nested Emotional Tasks Meta-Analysis of Bipolar Disorder Hypoactivation Alone (93 Experiments)

| **Ventrolateral Prefrontal Cortex: 32/30/-14, *k*=122, *z*=5.01** | | | | | | | |
| --- | --- | --- | --- | --- | --- | --- | --- |
| **Experiment ID** | **Contribution Score** | **Contrast/ Direction** | **Mood State** | **Diagnostic Subtype** | **Medication Status** | **Psychosis** | **Control Group** |
| Favre et al. 2013(119) | 6.63 | C>BD | Euthymic | BD I/II | >75% medicated | Not reported | Non-Clinical |
| Foland et al. 2008(106) | 4.35 | C>BD perceive emotion > control | Manic | BD I | >75% medicated | Not reported | Non-Clinical |
| Foland et al. 2008(106) | 9.39 | C>BD label emotion > control | Manic | BD I | >75% medicated | Not reported | Non-Clinical |
| Foland-Ross et al. 2012(62) | 16.55 | C>BD | Euthymic | BD I | <75% medicated | Not reported | Non-Clinical |
| Jogia et al. 2008(120) | 8.73 | C>BD | Euthymic | BD I | Unmedicated | Not reported | Non-Clinical |
| Killgore et al. 2008(121) | 2.24 | C>BD Block B | Not Specified | Not Reported | 100% medicated | Not reported | Non-Clinical |
| Manelis et al. 2016(47) | 3.06 | C>BD feedback | Depressed | BD I | 100% medicated | No current psychosis | Non-Clinical |
| Rey et al. 2014(63) | 1.71 | C>BD incongruent > congruent | Manic | NOS | 100% medicated | Not reported | Non-Clinical |
| Townsend et al. 2013(58) | 6.84 | C>BD (Table 2) | Euthymic | BD I | <75% medicated | Not reported | Non-Clinical |
| Trost et al. 2014(122) | 14.76 | C>BD (Table 2) | Not specified | BD I | >75% medicated | Not reported | Non-Clinical |
| Yip et al. 2015(115) | 15.32 | C>BD outcome phase: wins | Euthymic | NOS | Unmedicated | Not reported | Non-Clinical |
| Yip et al. 2015(115) | 3.96 | C>BD outcome phase: losses | Euthymic | NOS | Unmedicated | Not reported | Non-Clinical |
| Yip et al. 2015(115) | 5.24 | C>BD outcome phase: losses > neutral | Euthymic | NOS | Unmedicated | Not reported | Non-Clinical |

Abbreviations: BD = Bipolar Disorder; C = Controls; NCR = Non-Clinical Relatives; MDD = Major Depressive Disorder; NOS = Not Otherwise Specified.

**Supplementary Table 27.** Studies Contributing to the Amygdala Cluster from the Pooled Mixed Valence Meta-Analysis of 32 Studies

| **Amygdala: -28/-8/-24, *k*=196, *z*=3.77** | |
| --- | --- |
| **Study ID** | **Contribution Score** |
| Chase et al. 2013(123) | 8.55 |
| Dutra et al. 2017(36) | 5.22 |
| Korgaonkar et al. 2019(107) | 6.48 |
| Malhi et al. 2005(44) | 23.81 |
| Man et al. 2019(46) | 2.46 |
| Manelis et al. 2016(47) | 15.82 |
| Mitchell et al. 2004(110) | 10.42 |
| Negoias et al. 2019(50) | 5.65 |
| Perlman et al. 2012(111) | 10.8 |
| Rey et al. 2014(63) | 3.19 |
| Willert et al. 2015(124) | 6.96 |

**Supplementary Table 28.** Experiments Contributing to the Amygdala Cluster from the Nested Mixed Valence Meta-Analysis of 77 Experiments

| **Amygdala: -20/-12/-12, *k*=232, *z*=4.06** | | | | | | | |
| --- | --- | --- | --- | --- | --- | --- | --- |
| **Experiment ID** | **Contribution Score** | **Contrast/ Direction** | **Mood State** | **Diagnostic Subtype** | **Medication Status** | **Psychosis** | **Control Group** |
| Chase et al. 2013(123) | 7.33 | BD>C anticipation | Depressed | BD I | 100% medicated | No current psychosis | Non-Clinical |
| Dutra et al. 2017(36) | 5.92 | F_CBD R VS seed (BD>C) | Euthymic | BD I | 100% medicated | Not reported | Non-Clinical |
| Dutra et al. 2017(36) | 5.46 | F_CBD L VS seed (BD>C) | Euthymic | BD I | 100% medicated | Not reported | Non-Clinical |
| Korgaonkar et al. 2019(107) | 4.33 | F_CBD subliminal emotion processing (MDD>BD) | Euthymic | BD I | 100% medicated | Not reported | Clinical: MDD |
| Malhi et al. 2005(44) | 18.06 | F_CBD (C>BD) | Euthymic | BD I | <75% medicated | Not reported | Non-Clinical |
| Man et al. 2019(46) | 2.93 | F_CBD L amygdala | Not specified | BD I | 100% medicated | Not reported | Clinical: MDD |
| Manelis et al. 2016(47) | 10.32 | BD>C feedback | Depressed | BD I | 100% medicated | No current psychosis | Non-Clinical |
| Manelis et al. 2016(47) | 2.01 | C>BD feedback | Depressed | BD I | 100% medicated | No current psychosis | Non-Clinical |
| Mitchell et al. 2004(110) | 4.96 | C>BD pure | Not specified | Not reported | 100% medicated | Not reported | Non-Clinical |
| Mitchell et al. 2004(110) | 7.05 | BD>C unfiltered | Not specified | Not reported | 100% medicated | Not reported | Non-Clinical |
| Mitchell et al. 2004(110) | 3.68 | C>BD unfiltered | Not specified | Not reported | 100% medicated | Not reported | Clinical: SZ |
| Negoias et al. 2019(50) | 3.63 | BD>C (Table 3) | Euthymic | BD I/II | Not reported | Not reported | Non-Clinical |
| Perlman et al. 2012(111) | 6.08 | F_CBD (BD>C) | Euthymic | BD I | 100% medicated | No current psychosis | Non-Clinical |
| Rey et al. 2014(63) | 2.16 | C>BD group x load x congruence | Depressed | NOS | 100% medicated | Not reported | Non-Clinical |
| **Amygdala: -20/-12/-12, *k*=232, *z*=4.06** | | | | | | | |
| **Experiment ID** | **Contribution Score** | **Contrast/ Direction** | **Mood State** | **Diagnostic Subtype** | **Medication Status** | **Psychosis** | **Control Group** |
| Willert et al. 2015(124) | 7.17 | C>BD | Euthymic | BD I | 100% medicated | Not reported | Non-Clinical |
| Willert et al. 2015(124) | 8.08 | C>BD | Euthymic | BD I | 100% medicated | Not reported | Clinical: NCR |

Abbreviations: BD = Bipolar Disorder; C = Controls; F_CBD = Main effect of group or group x condition interaction; NCR = Non-Clinical Relatives; L = Left; MDD = Major Depressive Disorder; NOS = Not Otherwise Specified; R = Right; SZ = Schizophrenia.

**Supplementary Table 29.** Studies Contributing to the Striatum Cluster from the Euthymic Bipolar Disorder & Pooled Emotional Tasks Meta-Analysis of 39 Studies

| **Striatum: 10/10/-6, *k*=131, *z*=4.5** | |
| --- | --- |
| **Study ID** | **Contribution Score** |
| Dutra et al. 2015(28) | 23.13 |
| Dutra et al. 2017(36) | 14.29 |
| Foland-Ross et al. 2012(62) | 12.57 |
| Hassel et al. 2008(125) | 5.61 |
| Lagopoulos & Malhi 2011(40) | 8.72 |
| Malhi et al. 2005(44) | 5.04 |
| Malhi et al. 2007(45) | 6.59 |
| Negoias et al. 2019(50) | 4.67 |
| Rey et al. 2014(63) | 8.23 |
| Townsend et al. 2013(58) | 10.4 |

**Supplementary Table 30.** Experiments Contributing to the Striatum Cluster from the Euthymic Bipolar Disorder & Nested Emotional Tasks Meta-Analysis of 101 Experiments

| **Striatum: 10/10/-6, *k*=112, *z*=5.07** | | | | | | | |
| --- | --- | --- | --- | --- | --- | --- | --- |
| **Experiment ID** | **Contribution Score** | **Contrast/**  **Direction** | **Mood State** | **Diagnostic Subtype** | **Medication Status** | **Psychosis** | **Control Group** |
| Dutra et al. 2015(28) | 21.82 | F_CBD reward receipt | Euthymic | BD I | 100% medicated | Not reported | Non-Clinical |
| Dutra et al. 2017(36) | 17.25 | F_CBD R VS seed (BD>C) | Euthymic | BD I | 100% medicated | Not reported | Non-Clinical |
| Dutra et al. 2017(36) | 17.25 | F_CBD L VS seed (BD>C) | Euthymic | BD I | 100% medicated | Not reported | Non-Clinical |
| Foland-Ross et al. 2012(62) | 14.32 | C>BD | Euthymic | BD I | <75% medicated | Not reported | Non-Clinical |
| Lagopoulos & Malhi 2011(40) | 9.83 | BD>C Table 3 | Euthymic | BD I | <75% medicated | Not reported | Non-Clinical |
| Malhi et al. 2005(44) | 2.4 | F_CBD (C>BD) | Euthymic | BD I | <75% medicated | Not reported | Non-Clinical |
| Negoias et al. 2019(50) | 0.75 | BD>C (Table 3) | Euthymic | BD I/II | Not Reported | Not reported | Non-Clinical |
| Negoias et al. 2019(50) | 3.68 | BD>C (Table 4) | Euthymic | BD I/II | Not Reported | Not reported | Non-Clinical |
| Rey et al. 2014(63) | 10.14 | C>BD fear>joy | Euthymic | NOS | 100% medicated | Not reported | Non-Clinical |
| Townsend et al. 2013(58) | 1.34 | C>BD Table 2 | Euthymic | BD I | <75% medicated | Not reported | Non-Clinical |

Abbreviations: BD = Bipolar Disorder; C = Controls; F_CBD = Main effect of group or group x condition interaction; NC = Non-Clinical Controls; NCR = Non-Clinical Relatives; NOS = Not Otherwise Specified; PPI = Psychophysiological Interaction.

**Supplementary Table 31.** Studies Contributing to the Ventrolateral Prefrontal Cortex and Dorsal Anterior Cingulate Clusters from the Pooled Exploratory Task-Based Activation Meta-Analysis of 132 Studies

| **Ventrolateral Prefrontal Cortex: 32/30/-14, *k*=134, *z*=5.02** | |
| --- | --- |
| **Study ID** | **Contribution Score** |
| Almeida et al. 2010(126) | 4.38 |
| Favre et al. 2013(119) | 5.72 |
| Foland et al. 2008(106) | 5.17 |
| Foland-Ross et al. 2012(62) | 10.88 |
| Frangou 2012(38) | 7.45 |
| Jogia et al. 2008(120) | 8.68 |
| Killgore et al. 2008(121) | 0.85 |
| Manelis et al. 2016(47) | 4.71 |
| Mechelli et al. 2008(127) | 4.76 |
| Monks et al. 2004(95) | 0.87 |
| Morris et al. 2012(118) | 3.6 |
| Negoias et al. 2019(50) | 8.52 |
| Oertel-Knöchel et al. 2013(51) | 2.1 |
| Pompei et al. 2011(88) | 7.37 |
| Shaffer et al. 2018(55) | 0.47 |
| Townsend et al. 2013(58) | 0.68 |
| Trost et al. 2014(128) | 10.66 |
| Yip et al. 2015(115) | 11.02 |
| **Dorsal Anterior Cingulate Cortex: 4/34/4, *k*=216, *z*=4.78** | |
| **Study ID** | **Contribution Score** |
| Almeida et al. 2010(126) | 2.47 |
| Alonso-Lana et al. 2016(77) | 2.66 |
| Alonso-Lana et al. 2016(102) | 5.28 |
| Blumberg et al. 2005(129) | 1.66 |
| Dima et al. 2016(130) | 4.99 |
| Dima et al. 2016(131) | 3.93 |
| Elliott et al. 2004(132) | 3.33 |
| Fernández-Corcuera et al. 2013(101) | 2.21 |
| Foland et al. 2008(106) | 1.36 |
| Goikolea et al. 2019(79) | 8.03 |
| Jogia et al. 2012(133) | 1.23 |
| Killgore et al. 2008(121) | 2.7 |
| Lagopoulos & Malhi 2007(108) | 3.02 |
| **Dorsal Anterior Cingulate Cortex: 4/34/4, *k*=216, *z*=4.78** | |
| **Study ID** | **Contribution Score** |
| Liberg et al. 2012(80) | 1.31 |
| Malhi et al. 2007(45) | 0.65 |
| Manelis et al. 2016(47) | 0.85 |
| Mason et al. 2014(134) | 4.35 |
| Negoias et al. 2019(50) | 11.68 |
| Poletti et al. 2017(135) | 2.79 |
| Pomarol-Clotet et al. 2012(104) | 3.82 |
| Pomarol-Clotet et al. 2015(52) | 1.01 |
| Robinson et al. 2009(81) | 7.11 |
| Rodríguez-Cano et al. 2017(82) | 4.47 |
| Sepede et al. 2015(136) | 3.64 |
| Sugihara et al. 2017(99) | 0.21 |
| Townsend et al. 2013(83) | 3.44 |
| Vizueta et al. 2012(113) | 5.45 |
| Yip et al. 2015(115) | 4.54 |

**Supplementary Table 32.** Experiments Contributing to the Premotor Cortex and Orbitofrontal Cortex Clusters from the Pooled Exploratory Working Memory Paradigms Meta-Analysis of 28 Studies

| **Premotor Cortex: -32/-2/52, *k*=158, *z*=5.76** | |
| --- | --- |
| **Study ID** | **Contribution Score** |
| Alonso-Lana et al. 2019(103) | 19.01 |
| Fernández-Corcuera et al. 2013(101) | 16.93 |
| Monks et al. 2004(95) | 12.46 |
| Pomarol-Clotet et al. 2012(104) | 21.25 |
| Pomarol-Clotet et al. 2015(52) | 29.76 |
| **Orbitofrontal Cortex: -2/52/-16, *k*=129, *z*=5.42** | |
| **Study ID** | **Contribution Score** |
| Pomarol-Clotet et al. 2015(52) | 13.88 |
| Goikolea et al. 2019(79) | 22.29 |
| Rodríguez-Cano et al. 2017(82) | 26.3 |
| Alonso-Lana et al. 2016(77) | 18.46 |
| Robinson et al. 2009(81) | 18.21 |

**Supplementary Table 33.** Descriptions of the 205 Studies Included in the Meta-Analyses

| **Publication** | **Analyzed Participant Groups, N** | **Mood State(s)** | **BD Mean Age (SD)** | **Medication Status**  **No. (%)** | **Psychosis**  **No. (%)** | **Imaging Modality** | **Design/Paradigm** | **Contrast(s) Used** |
| --- | --- | --- | --- | --- | --- | --- | --- | --- |
| Adler et al. 2004(27) | 15 BD, 15 NC | Euthymic (n=15) | 29 (9) | 10 (67) | Not reported | fMRI | N-back (C) | BD>NC (Table 2) |
| Allin et al. 2010(137) | 18 BD I, 19 NC, 19 NCR | Euthymic (n=18) | 39.2 (11.5) | 13 (72) | 18 (100) with history | fMRI | Verbal fluency (C) | Main effect of group (Fig. 1) |
| Almeida et al. 2009(138) | 21 BD I, 25 NC | Euthymic (n=21) | 31.95 (8.47) | 19 (90) | No current features | fMRI | Labeling happy and neutral faces (E) | Main effect of group; group x condition int. (Table S2) |
| Almeida et al. 2010(126) | 30 BD I, 15 MDD, 15 NC | Euthymic (n=15), Depressed (n=15) | 34.92 (9.86)^[[3]](#footnote-3)^ | 27 (90) | No current features | fMRI | Labeling happy, sad, fearful, and neutral faces (E) | Group x happy int.; group x sad int.; main effect of group during sad; main effect of group during fear; group x fear int. (Table S6) |
| Alonso-Lana et al. 2016(102) | 27 BD I, 28 NC | Euthymic (27) | 44.49 (6.99) | 27 (100) | Not reported | fMRI | N-back (C) | BD>C 2-back>BL; BD>C 2-back>1-back |
| Alonso-Lana et al. 2016(77) | 20 BD I/II, 40 NC, 20 NCR | Euthymic (n=20) | 41.02 (10.83) | 20 (100) | Not reported | fMRI | N-back (C) | Main effect of group 2-back>BL |
| Alonso-Lana et al. 2019(103) | 26 BD I, 26 NC | Hypo/manic at BL (n=26) | 39.19 (12.33) | 26 (100) | Not reported | fMRI | N-back (C) | NC>BD, BD>NC (Session 1) |
| Altinay et al. 2016(34) | 60 BD I/II, 30 NC | Depressed (n=30), Hypo/manic (n=30) | 33.5 (11)^[[4]](#footnote-4)^ | 60 (0) | 19 (32) with history | fMRI | Resting state (seed-to-voxel: 7 striatum seeds) | 7 main effects of group (Table 3) |
| Ambrosi et al. 2017(139) | 36 BD I/II, 40 MDD | Depressed (n=36) | 31 (11.3) | 36 (100) | 5 (14) with current features | fMRI | Resting state (seed-to-voxel: bilateral insula and amygdala) | MDD>BD L insula; MDD>BD R insula; BD>MDD R amygdala |
| **Publication** | **Analyzed Participant Groups, N** | **Mood State(s)** | **BD Mean Age (SD)** | **Medication Status**  **No. (%)** | **Psychosis**  **No. (%)** | **Imaging Modality** | **Design/Paradigm** | **Contrast(s) Used** |
| Anticevic et al. 2014(75) | 73 BD I (33 with psychosis history, 40 without), 56 NC, 90 SZ | Euthymic (n=73) | 32 (11.23)^[[5]](#footnote-5)^ | 61 (84) | 33 (45) with history | fMRI | Resting state (seed-to-voxel: 2 thalamic seeds – lateral geniculate and mediodorsal L/R/midline) | 5 main effects of group BD vs NC (Table 3); 7 BD>SZ and 2 SZ>BD (Table 5) |
| Argyelan et al. 2014(35) | 19 BD, 18 SZ, 32 NC | Not specified (n=19) | 40.6 (13.3) | 19 (100) | 16 (84) with history | fMRI | Resting state (connectivity strength) | BD>NC, NC>BD, BD>SZ; SZ>BD (Table S1) |
| Avery et al. 2014(140) | 17 BD I, 22 NC | Depressed (n=5), Euthymic (n=12) | 37.41 (10.37) | 16 (94) | 17 (100) with history | fMRI | Transitive inference to assess relational memory (EC) | NC>BD (Table 3) |
| Benson et al. 2008(84) | 30 BD I/II, 66 NC | Depressed (n=30) | 36.4 (10.6) | 30 (0) | Not reported | FDG PET | Auditory CPT (C) (seed-to-voxel: 5 bilateral seeds: insula, inferior parietal cortex, DLPFC, thalamus, cerebellum) | 10 main effects of group (Table 2) |
| Benson et al. 2014(78) | 30 BD I/II, 66 NC | Depressed (n=30) | 36.4 (10.6) | 30 (0) | Not reported | FDG PET | Auditory CPT (C) (seed-to-voxel: bilateral amygdala, hippocampus) | 4 main effects of group (Table 1) |
| Bermpohl et al. 2010(141) | 15 BD I, 26 NC | Hypo/manic (n=15) | 38.6 (13.7) | 15 (100) | Not reported | fMRI | Monetary incentive delay (E) | NC>BD cued incentive valence, BD>NC expected value |
| Berns et al. 2002(142) | 13 BD II, 14 NC | Euthymic (n=13) | 31.2 (8.3) | 9 (69) | Not reported | [O]H_2_O PET | Serial reaction time (C) (regional CBF change associated with motor task) | Group x sequence change interaction (Table 1) |
| Blumberg et al. 1999(143) | 5 BD I, 5 NC | Hypo/manic (n=5) | 34.2 (12.2) | 5 (100) | Not reported | [O]H_2_O PET | Word generation (C) | NC>BD during rest condition and word generation |
| **Publication** | **Analyzed Participant Groups, N** | **Mood State(s)** | **BD Mean Age (SD)** | **Medication Status**  **No. (%)** | **Psychosis**  **No. (%)** | **Imaging Modality** | **Design/Paradigm** | **Contrast(s) Used** |
| Blumberg et al. 2003(144) | 36 BD I, 20 NC | Hypo/manic (n=10), Mixed (n=1), Depressed (n=10), Euthymic (n=15) | 39.1 (10) | 23 (64) | 8 (22) with history, 1 (3) with current features | fMRI | Stroop (C) | Main effect of group (trait analysis: group differences in signal change) |
| Blumberg et al. 2005(129) | 17 BD I, 17 NC | Mixed (n=2), Euthymic (n=9), Depressed (n=3), Hypo/ manic (n=3) | 44.09 (10.25)^[[6]](#footnote-6)^ | 12 (70) | Not reported | fMRI | Viewing happy, sad, and fearful faces (E) | Main effect of group (Fig. 3) |
| Bøen et al. 2019(145) | 22 BD II, 22 BPD, 21 NC | Not specified (n=22) | 32.6 (6) | 19 (86) | No history | FDG PET | Resting state | NC>BD, BD>NC, BD>BPD (Table 2) |
| Brady Jr. et al. 2016(146) | 52 BD I, 23 NC | Hypo/manic (n=28), Euthymic (n=24) | 29.07 (11.25)^[[7]](#footnote-7)^ | 52 (100) | 52 (100) with history | fMRI | Resting state (seed-to-voxel: bilateral amygdala) | Main effect of group (Table 2 bilateral amygdala) |
| Brady Jr. et al. 2017(65)* | 24 BD I, 23 NC | Euthymic (n=24) | 30.9 (11.9) | 24 (100) | 24 (100) with history | fMRI | Resting state (seed-to-voxel: parietal-occipital and dorsal frontal lobes 38/61) | NC>BD for ROIs 38, 61, and 97 |
| Brandt et al. 2014(147) | 100 BD I/II, 100 SZ | Remitted (n=74), Depressed (n=21), Hypo/manic (n=4), Psychotic (n=1) | 35.4 (11.4) | 100 (100) | 52 (52) with history, 17 (17) with current features | fMRI | N-back (C) (ICA) | SZ>BD component 8 |
| **Publication** | **Analyzed Participant Groups, N** | **Mood State(s)** | **BD Mean Age (SD)** | **Medication Status**  **No. (%)** | **Psychosis**  **No. (%)** | **Imaging Modality** | **Design/Paradigm** | **Contrast(s) Used** |
| Brooks III et al. 2009(148) | 16 BD I/II, 11 NC | Euthymic (n=16) | 58.7 (7.5) | 15 (94) | Not reported | FDG PET | Resting state | NC>BD, BD>NC (Table 3) |
| Brooks III et al. 2009(149) | 16 BD I/II, 11 NC | Euthymic (n=16) | 58.7 (7.5) | 16 (100) | Not reported | FDG PET | Resting state (cerebral metabolism correlated with verbal recall score) | NC>BD, BD>NC (Table 2) |
| Brooks III et al. 2009(150) | 15 BD I/II, 19 NC | Depressed (n=15) | 36.1 (10.4) | 15 (0) | Not reported | FDG PET | Resting state | NC>BD (Table 2) |
| Brooks III et al. 2010(151) | 16 BD I/II, 11 NC | Euthymic (n=16) | 58.7 (7.5) | 15 (94) | Not reported | FDG PET | Resting state (cerebral metabolism correlated with CPT score) | 3 BD>NC, 3 NC>BD (Table 2) |
| Brooks III et al. 2010(152) | 8 BD I, 8 NC | Hypo/manic (n=8) | 52.75 (5.75) | 7 (87) | 8 (100) with current features | FDG PET | Resting state | NC>BD, BD>NC (Table 2) |
| Brooks III et al. 2015(91) | 19 BD II, 19 NC | Depressed (n=19) | 36.7 (11.4) | 19 (0) | Not reported | fMRI | N-back (C) | NC>BD (Table 3) |
| Brown et al. 2012(85) | 18 BD I, 18 NC | Not specified (n=18) | 33.8 (12.3) | 10 (55) | Not reported | fMRI | N-back (C) | NC>BD |
| Bürger et al. 2017(153) | 36 BD, 36 UD | Depressed (n=36) | 38.56 (12.3) | 33 (92)^[[8]](#footnote-8)^ | Not reported | fMRI | Face-matching with fearful faces (E) | NC>BD |
| Cantisani et al. 2016(154) | 22 BD, 19 NC, 20 UD | Depressed (n=22) | 46.18 (11.21) | 22 (100) | Not reported | ASL | Resting state | Main effect of group (Table S1) |
| Caseras et al. 2013(155) | 32 BD I/II, 20 NC | Euthymic (n=32) | 41.75 (7.68)^[[9]](#footnote-9)^ | 26 (81) | No current features | fMRI | Card-guessing with monetary rewards (E) | Main effect of group during punishment anticipation (Supplement) |
| Cerullo et al. 2012(156) | 15 BD I, 15 NC | Hypo/manic/mixed at BL (n=15) | 30 (9.8) | 15 (100) | No history | fMRI | CPT with emotional and neutral distractors (CPT-END) (EC) (seed-to-voxel: amygdala) | Main effect of group in R amygdala (Table 4) |
| **Publication** | **Analyzed Participant Groups, N** | **Mood State(s)** | **BD Mean Age (SD)** | **Medication Status**  **No. (%)** | **Psychosis**  **No. (%)** | **Imaging Modality** | **Design/Paradigm** | **Contrast(s) Used** |
| Chai et al. 2011(157) | 14 BD I, 16 SZ, 15 NC | Hypo/manic (n=14) | 32.7 (3) | 14 (100) | 14 (100) with current features | fMRI | Resting state (seed-to-voxel: MPFC) | Main effect of group (Table 2) |
| Chakirova et al. 2011(158) | 36 BD I, 20 SZ, 33 NC | Not specified (n=36) | 39.3 (10.8) | 36 (100) | 36 (100) with current features | fMRI | Sentence completion (C) | Main effect of group (Table S2) |
| Chase et al. 2013(123) | 23 BD I, 40 MDD, 37 NC | Depressed (n=23) | 33.94 (8.51) | 23 (100) | No current features | fMRI | Card-guessing with monetary rewards (E) | Reward expectancy: BD>MDD, MDD>BD, BD>NC (Table S4.1.2); Anticipation: BD>NC, NC>BD, BD>MDD, MDD>BD, (Table S4.2.5); Prediction error: BD>NC, NC>BD, BD>MDD (Table S4.3.2) |
| Chen et al. 2006(105) | 16 BD I, 8 NC | Hypo/manic (n=8), Depressed (n=8) | 40.44 (12.76)^[[10]](#footnote-10)^ | 16 (100) | 8 (50) with current features (manic patients) | fMRI | Explicit and implicit facial affect recognition with happy, fearful, and sad faces (E) | Sad: main effect of group, group x sad int.; Fear: main effect of group; Happy: main effect of group (Table 2) |
| Chen et al. 2019(159) | 90 BD II, 100 NC | Depressed (n=90) | 26.74 (8.73) | 90 (0) | Not reported | fMRI | Resting state (seed-to-voxel: 2 cerebellum seeds) | BD>NC, NC>BD (Table 2) |
| Chepenik et al. 2010(61) | 15 BD, 10 NC | Euthymic (n=8), Depressed (n=2), Hypo/ manic/mixed (n=5) | 43 (9.9) | 12 (80) | Not reported | fMRI | Resting state (seed-to-voxel: ventral PFC) | BD>NC (Fig. 2) |
| Corbalán et al. 2015(24) | 19 BD I, 17 NC | Euthymic (n=19) | 41 (12.5) | 15 (19)^[[11]](#footnote-11)^ | Not reported | fMRI | Emotion regulation of negative and neutral images (E) | Instruction x valence x group int. (negative and neutral decrease minus look); group x instruction int. (negative decrease vs. negative look) |
| **Publication** | **Analyzed Participant Groups, N** | **Mood State(s)** | **BD Mean Age (SD)** | **Medication Status**  **No. (%)** | **Psychosis**  **No. (%)** | **Imaging Modality** | **Design/Paradigm** | **Contrast(s) Used** |
| Curtis et al. 2007(160) | 12 BD I, 12 NC | Euthymic (n=12) | 45.8 (9.6) | 12 (100) | Not reported | fMRI | Verbal fluency (C) | Group x task effect; group x task demand x reaction time (Table 3) |
| Deckersbach et al. 2006(23) | 8 BD I, 8 NC | Euthymic (n=8) | 27.25 (5.6) | 1 (12)^[[12]](#footnote-12)^ | Not reported | ^15^[O]CO_2_ PET | Verbal learning (C) | Encoding: NC>BD, BD>NC (Table 4); spontaneous> unrelated/directed encoding: NC>BD, BD>NC (Table 5); spontaneous/ directed>unrelated encoding: NC>BD, BD>NC (Table 5) |
| Deckersbach et al. 2008(161) | 9 BD I, 17 NC | Depressed (n=9) | 27.6 (2.8) | 9 (100) | Not reported | fMRI | N-back with sad mood induction (EC) | BD>NC N-back>fixation; BD>NC mood induction>no induction (Table 5) |
| Dell’Osso et al. 2015(162) | 28 BD I/II, 27 NC | Euthymic (n=28) | 35.7 (9.2) | 28 (100) | Not reported | fMRI | N-back (C) | Main effect of group (Table S2) |
| Delvecchio et al. 2015(163) | 41 BD I, 46 NC, 25 NCR | Euthymic (n=41) | 44.3 (11.9) | 30 (73) | Some with history (% not reported – taken from the VIBES cohort(164) | fMRI | N-back (C) | Main effect of group in 3-back>0-back (Table S3) |
| Deng et al. 2019(165) | 23 BD I, 46 NC | Not specified (n=23) | 23.61 (4.93) | 23 (100) | 16 (70) with current features | fMRI | Resting state (degree centrality) | NC>BD, BD>NC (Table 2) |
| Dima et al. 2016(130) | 41 BD I, 46 NC, 25 NCR | Euthymic (n=41) | 44.3 (11.9) | 41 (100) | Some with history (% not reported – taken from the VIBES cohort(164) | fMRI | Facial affect recognition with fearful, angry, and sad faces (E) | Main effect of group for affect>neutral faces (Table S2) |
| **Publication** | **Analyzed Participant Groups, N** | **Mood State(s)** | **BD Mean Age (SD)** | **Medication Status**  **No. (%)** | **Psychosis**  **No. (%)** | **Imaging Modality** | **Design/Paradigm** | **Contrast(s) Used** |
| Dima et al. 2016(131) | 41 BD I, 46 NC, 25 NCR | Euthymic (n=41) | 44.3 (11.9) | 41 (100) | Some with history (% not reported – taken from the VIBES cohort(164) | fMRI | 2: N-back (C); Facial affect recognition with fearful, angry, and sad faces (E) | Main effect of group for affect>neutral faces and 3-back>0-back (Table S2) |
| Drapier et al. 2008(92) | 20 BD I, 20 NC | Euthymic (n=20) | 42.7 (10.4) | 16 (80) | 20 (100) with history | fMRI | N-back (C) | 1-back: BD>NC direct comparison; 2-back: BD>NC direct comparison |
| Dutra et al. 2015(28) | 24 BD I, 25 NC | Euthymic (n=24) | 31.38 (11.86) | 24 (100) | Not reported | fMRI | 2: Monetary incentive delay (E) and Social incentive delay (E) | 2 main effects of group (Table 2 reward anticipation; Table 3 reward receipt) |
| Dutra et al. 2017(36) | 24 BD I, 25 NC | Euthymic (n=24) | 31.38 (11.86) | 24 (100) | Not reported | fMRI | 2: Monetary incentive delay (E) and Social incentive delay (E) (seed-to-voxel: R/L ventral striatum) | R and L ventral striatum beta estimates x task outcome x group int. (Table 2) |
| Ellard et al. 2018(37)* | 24 BD I/II, 39 NC | Depressed (n=24) | 45.58 (14.97) | 24 (100) | No history | fMRI | Resting state (seed-to-voxel: R/L dorsal and ventral anterior insula) | BD>NC/NC>BD L dorsal insula, BD>NC/NC>BD L ventral insula, BD>NC/ NC>BD R dorsal insula, BD>NC/NC>BD R ventral insula |
| Elliott et al. 2004(132) | 8 BD, 11 NC | Hypo/manic (n=8) | 33.5 (SD not reported) | 7 (87) | 5 (62) with history; 2 (25) with current features | fMRI | Go/NoGo with happy, sad, and neutral words (EC) | NC>BD/BD>NC (Table 2, Table 3, Table 4), BD>NC sad distractors (Table 5), BD>NC happy distractors (Table 5) |
| Fateh et al. 2019(166) | 30 BD, 30 NC, 29 MDD | Depressed (n=30) | 34.26 (9.79) | 30 (100) | Not reported | fMRI | Resting state (seed-to-voxel: R/L anterior and posterior hippocampus) | 4 main effects of group (for each of the 4 seeds; Table 3) |
| **Publication** | **Analyzed Participant Groups, N** | **Mood State(s)** | **BD Mean Age (SD)** | **Medication Status**  **No. (%)** | **Psychosis**  **No. (%)** | **Imaging Modality** | **Design/Paradigm** | **Contrast(s) Used** |
| Favre et al. 2013(119) | 16 BD I/II, 16 NC | Euthymic at BL (n=16) | 40.4 (11.8) | 15 (94) | Not reported | fMRI | Word-face emotional Stroop with happy, fearful, and neutral faces (EC) | NC>BD x Stroop int., BD>NC x valence int. (Table 3, excluding ROIs) |
| Favre et al. 2014(167) | 20 BD I/II/NOS, 20 NC | Euthymic (n=20) | 42 (10.7) | 20 (100) | Not reported | fMRI | Resting state (seed-to-voxel: VMPFC) | BD>NC (Results 3.1/Fig. 1) |
| Favre et al. 2015(168) | 14 BD I/II/NOS, 13 NC | Euthymic (n=14) | 44.1 (9.6) | 12 (86) | 6.5 (46) with history | fMRI | Word-face emotional Stroop with happy and fearful faces (EC) (PPI: DLPFC) | NC>BD (Table 3, monitoring); BD>NC (Table 4, PPI positive regression) |
| Fernández-Corcuera et al. 2013(101) | 41 BD, 41 NC | Depressed (n=41) | 40.39 (10.2) | 41 (100) | 41 (100) with current features | fMRI | N-back (C) | NC>BD/BD>NC 2-back>BL (Table 2), NC>BD/BD>NC increasing working memory load (Results 3.5) |
| Fleck et al. 2011(96) | 8 BD I, 10 NC | Mixed (n=8) | 30 (8) | 7 (87) | Not reported | fMRI | Go/NoGo (C) | BD>NC (Results 3.3) |
| Foland et al. 2008(106) | 9 BD I, 9 NC | Hypo/manic (n=9) | 34.6 (8) | 7 (78) | Not reported | fMRI | Labeling and matching fearful and angry faces (E) | BD>NC/NC>BD perceive emotion > control and label emotion > control (Table 2) |
| Foland-Ross et al. 2012(62) | 24 BD I, 26 NC | Euthymic (n=24) | 38.8 (12.8) | 16 (67) | Not reported | fMRI | Labeling and matching fearful and angry faces (E) | NC>BD label emotion > match forms (Table 2) |
| Fournier et al. 2013(169) | 22 BD I, 30 MDD | Depressed (n=22) | 34 (8.2) | 22 (100) | No current features | fMRI | Implicit viewing of angry, fearful, sad and happy faces (E) | Group x emotion int. (Table 2) |
| Fournier et al. 2016(170) | 16 BD I, 19 NC, 19 UD | Depressed (n=16) | 35.22 (8.91) | 16 (100) | No current features | fMRI | Implicit viewing of angry, fearful, sad and happy faces (E) (within-session linear attenuation of BOLD signal) | Main effect of group (linear BOLD attenuation, Table 3) |
| Frangou et al. 2008(171) | 7 BD I, 7 NC | Euthymic (n=7) | 37 (5.88) | 7 (100) | Not reported | fMRI | N-back (C) | NC>BD, BD>NC (parametric analysis, Table 2) |
| **Publication** | **Analyzed Participant Groups, N** | **Mood State(s)** | **BD Mean Age (SD)** | **Medication Status**  **No. (%)** | **Psychosis**  **No. (%)** | **Imaging Modality** | **Design/Paradigm** | **Contrast(s) Used** |
| Frangou 2012(38) | 47 BD I, 71 NC, 48 NCR | Euthymic (n=47) | 46.2 (11.5) | 39 (83) | Some with history (% not reported – taken from the VIBES cohort(164) | fMRI | Stroop (C) (PPI: VLPFC) | Main effect of group (results of fMRI); Main effect of group (Table 2 PPI) |
| Frangou et al. 2017(172) | 30 BD I, 30 NC, 30 NCR, 30 MDD Relatives | Euthymic (n=30) | 34.7 (7.7) | 30 (100) | Some with history (% not reported – taken from the VIBES cohort(164) | fMRI | N-back (C) | Main effect of group (conventional fMRI analysis) |
| Glahn et al. 2010(93) | 15 BD I, 24 NC | Euthymic (n=15) | 38 (13.1) | 14 (93) | Not reported | fMRI | Face-name paired-associative memory (C) | NC>BD and BD>NC encoding; NC>BD recognition |
| Goikolea et al. 2019(79) | 31 BD, 31 NC | Hypo/manic/mixed (n=31) | 30.52 (9.05) | 31 (100) | 31 (100) with history; 23 (74) with current features | fMRI | N-back (C) (PPI: MPFC) | BD>NC 2-back>BL, BD>NC PPI |
| Gong et al. 2019(66) | 96 BD II, 100 NC | Depressed (n=96) | 27.33 (9.2) | 96 (0) | Not reported | fMRI | Resting state (seed-to-voxel: PCC, subgenual ACC) | NC>BD L PCC and L subgenual ACC (Table 2) |
| Goya-Maldonado et al. 2016(67) | 20 BD I, 20 NC, 20 UD | Depressed (n=20) | 35.8 (10.2) | 18 (90) | Not reported | fMRI | Resting state (ICA: cingulo-opercular, default mode, and frontoparietal) | 3 main effects of group (for the 3 networks, Table 2) |
| Grant et al. 2018(173) | 25 BD I, 25 NC | Euthymic and Depressed (n=25) | 35.4 (10.4) | Not reported | Not reported | fMRI | Theory of mind with negative and positive social scenarios (E) | BD>NC self-tNCReat>self-boost, self-tNCReat>other-tNCReat, self-boost>other-boost (Table 3) |
| Hall et al. 2010(174) | 14 BD I, 14 NC, 15 SZ | Not specified (n=14) | 40.8 (10.4) | 14 (100) | 12 (85) with history | fMRI | Face-name paired-associative memory (C) | Main effect of group for late retrieval (Table 2) |
| **Publication** | **Analyzed Participant Groups, N** | **Mood State(s)** | **BD Mean Age (SD)** | **Medication Status**  **No. (%)** | **Psychosis**  **No. (%)** | **Imaging Modality** | **Design/Paradigm** | **Contrast(s) Used** |
| Hamilton et al. 2009(175) | 21 BD I, 38 NC, 20 SZ | Euthymic (n=21) | 36.38 (10.7) | 17 (81) | Not reported | fMRI | Working memory (C) | NC>BD (Table 3), SZ>BD (Table 3) |
| Han et al. 2018(176) | 10 BD I, 10 NC | Euthymic (n=10) | 38.6 (9.4) | 10 (100) | Not reported | fMRI | 2: Viewing neutral, positive, and negative pictures (E); Rating tonic pain after exposure (E) | BD>NC positive>BL and BD>NC negative>BL (Table 3); NC>BD pain session (Table 4) |
| Han et al. 2019(177) | 40 BD, 44 NC, 54 MDD | Depressed (n=40) | 33.48 (9.71) | 40 (100) | Not reported | fMRI | Resting state (seed-to-voxel: dorsal/median raphe nucleus) | 2 main effects of group for dorsal and median raphe nuclei (Table 2) |
| Hassel et al. 2008(39) | 19 BD, 24 NC | Euthymic (n=19) | 32.47 (8.8) | 18 (95) | No current features | fMRI | Viewing happy, fearful, and neutral faces (E) | Group x happy int., main effect of group for happy; main effect of group for fear (Table 3) |
| He et al. 2019(178) | 25 BD, 34 NC, 25 MDD | Depressed (n=25) | 34.28 (8.65) | 21 (84) | Not reported | fMRI | Resting state (seed-to-voxel: caudate, putamen) | 2 main effects of group for R caudate and R putamen (Table 2) |
| Horacek et al. 2015(179) | 20 BD I, 20 NC | Euthymic (n=20) | 41.9 (12.9) | 20 (100) | Not reported | fMRI | Empathizing with sad and neutral faces and rating sadness after sad mood induction (E) (seed-to-voxel: amygdala) | Group x emotional challenge x laterality x facial expression int. |
| Hulvershorn et al. 2012(180) | 75 BD I/II, 30 NC | Depressed (n=30), Euthymic (n=15), Hypo/manic (n=30) | 33.8 (11)^[[13]](#footnote-13)^ | 75 (0) | 22 (29) with history | fMRI | Face-emotion matching with angry and fearful faces (E) | Main effect of group for faces>shapes (Fig. 4) |
| Hummer et al. 2013(181) | 74 BD I/II, 30 NC | Depressed (n=30), Euthymic (n=14), Hypo/manic (n=30) | 33.8 (11)^[[14]](#footnote-14)^ | 74 (0) | 22 (30) with history | fMRI | Go/NoGo with happy and sad faces (EC) | Main effect of group for happy inhibition and sad>happy inhibition (Table 1, *k*>108) |
| **Publication** | **Analyzed Participant Groups, N** | **Mood State(s)** | **BD Mean Age (SD)** | **Medication Status**  **No. (%)** | **Psychosis**  **No. (%)** | **Imaging Modality** | **Design/Paradigm** | **Contrast(s) Used** |
| Ives-Deliperi et al. 2013(182) | 23 BD I/II, 10 NC | Not specified at BL/Time 1 (n=23) | 37.6 (9.3) | Not reported | Not reported | fMRI | Meditation (E) | NC>BD for meditation>control at Time 1 |
| Jamadar et al. 2013(86) | 32 BD I/NOS, 133 NC, 74 SZ | Not specified (n=32) | 36.66 (2.1) | 21 (66) | 32 (100) with history; 20 (63) with current features | fMRI | Semantic Object Retrieval Task (C) | Main effect of group (Table 3) |
| Jogia et al. 2008(120) | 12 BD I, 12 NC | Euthymic at BL (n=12) | 42.1 (11.8) | 12 (0) | Not reported | fMRI | Facial affect recognition with sad and neutral faces (E) | NC>BD and BD>NC (Table 2) |
| Jogia et al. 2012(133) | 36 BD I, 37 NC | Euthymic (n=36) | 42.5 (10.6) | 22 (61) | 19 (53) with history | fMRI | 2: N-back (C), Iowa Gambling Task (E) | BD>NC (Table 2), BD>NC (Table 3), NC>BD (Table 3) |
| Jogia et al. 2012(183) | 39 BD I, 48 NC | Euthymic (n=39) | 39 (12) | 30 (77) | Some with history (% not reported – taken from the VIBES cohort(164) | fMRI | 2: Iowa Gambling Task (E), Fearful Facial Affect Categorization Task (E) | Sex-by-diagnosis interaction for Iowa Gambling; main effect of diagnosis and sex-by-diagnosis interaction for Fearful Faces |
| Joshi et al. 2016(97) | 45 BD I, 45 NC | Euthymic (n=45) | 39.9 (12.1) | 34 (76) | Not reported | fMRI | Go/NoGo (C) | NC>BD NoGo-Go (Table 2) |
| Kaladijan et al. 2009(184) | 10 BD I, 10 NC | Hypo/manic at BL (n=10) | 40.1 (13.7) | 10 (100) | Not reported | fMRI | Go/NoGo (C) | Main effect of group (Table 4) |
| Keener et al. 2012(185) | 27 BD I, 27 NC | Euthymic (n=27) | 31.67 (8.47) | 26 (96) | No current features | fMRI | Implicit viewing of angry, fearful, sad and happy faces (E) | BD>NC face emotion minus shapes, BD>NC face identity minus shapes, BD>NC fearful vs shapes, BD<NC happy vs shapes (between-group *t* contrasts at whole-brain level, Table 2) |
| **Publication** | **Analyzed Participant Groups, N** | **Mood State(s)** | **BD Mean Age (SD)** | **Medication Status**  **No. (%)** | **Psychosis**  **No. (%)** | **Imaging Modality** | **Design/Paradigm** | **Contrast(s) Used** |
| Killgore et al. 2008(121) | 14 BD, 13 NC | Not specified (n=14) | 28.1 (11.2) | 14 (100) | Not reported | fMRI | Viewing fearful faces (E) | NC>BD Block A, BD>NC Block B, NC>BD Block B (Table 1) |
| Kim et al. 2009(186) | 14 BD I, 14 NC | Euthymic (n=14) | 30.4 (5.9) | 14 (100) | 9 (64) with history; no current features | fMRI | Virtual reality social cognition task with happy, angry, and neutral faces (E) | BD>NC angry + happy, NC>BD angry, BD>NC happy, NC>BD happy, NC>BD neutral (Table 3) |
| Kirschner et al. 2019(187) | 25 BD I, 25 NC | Euthymic (n=25) | 37.3 (9.1) | 25 (100) | 25 (100) with current features | fMRI | Monetary Incentive Delay (E) | NC>BD reward anticipation (Table S7) |
| Korgaonkar et al. 2019(107) | 23 BD I, 25 MDD | Euthymic (n=23) | 33.48 (13.43) | 23 (100) | Not reported | fMRI | Supraliminal and subliminal emotion processing of angry, fearful, disgusted, sad, happy, and neutral faces (E) | Main effect of group for supraliminal processing (Table S3), main effect of group for subliminal processing and group x condition interaction (Table S4) |
| Lagopoulos et al. 2007(188) | 10 BD I, 10 NC | Euthymic (n=10) | 32.4 (10.8) | 7 (70) | Not reported | fMRI | Sternberg memory task (C) | 3 main effects of group (encode, delay, response: Table 5) |
| Lagopoulos & Malhi 2007(108) | 10 BD I, 10 NC | Euthymic (n=10) | 31.29 (8.12) | 7 (70) | Not reported | fMRI | Emotional Stroop with negative and neutral words (EC) | NC>BD, BD>NC (Table 2) |
| Lagopoulos & Malhi 2011(40) | 11 BD I, 11 NC | Euthymic (n=11) | 33.33 (9.23) | 8 (73) | Not reported | fMRI | Facial affect recognition with neutral and disgusted faces (E) | NC>BD, BD>NC (Table 2), NC>BD, BD>NC (Table 3) |
| Lee et al. 2019(189) | 55 BD I/II, 53 NC, 52 SZ | Euthymic (n=48), not specified (n=7) | 45.3 (12.1) | 55 (100) | 15 (27) with history | fMRI | Object discrimination (C) | NC>BD, SZ>BD (eTable 2), BD>NC, BD>SZ (eTable 3) |
| Lennox et al. 2004(117) | 10 BD I, 12 NC | Hypo/manic (n=10) | 37.3 (12.8) | 10 (100) | Not reported | fMRI | Explicit facial affect recognition with sad faces (E) | NC>BD and BD>SZ during sad faces (Table 2) |
| Li et al. 2018(190) | 19 BD I/II, 25 NC | Euthymic (n=19) | 38.79 (12.03) | 19 (100) | Not reported | fMRI | Resting state (seed-to-voxel: amygdala) | NC>BD (Table 3) |
| **Publication** | **Analyzed Participant Groups, N** | **Mood State(s)** | **BD Mean Age (SD)** | **Medication Status**  **No. (%)** | **Psychosis**  **No. (%)** | **Imaging Modality** | **Design/Paradigm** | **Contrast(s) Used** |
| Liang et al. 2013(68) | 17 BD, 16 NC, 16 UD | Depressed (n=17) | 34.47 (9.77) | 17 (0) | Not reported | fMRI | Resting state (ReHo) | NC>BD, BD>NC (Table 3), BD>UD (Table 4) |
| Liberg et al. 2013(80) | 9 BD I, 12 NC | Depressed (n=9) | 42.1 (SD not reported) | 9 (100) | Not reported | fMRI | Self-paced finger tapping (C) | NC>BD, BD>NC (Fig. 3) |
| Liberg et al. 2013(94) | 9 BD I, 12 NC | Depressed (n=9) | 42.1 (SD not reported) | 9 (100) | Not reported | fMRI | Self-paced finger tapping with motor imagery (C) | BD>NC motor imagery>rest, NC>BD motor execution>rest, BD>NC motor imagery>motor execution (Table 2) |
| Liu et al. 2012(41) | 26 BD, 26 NC | Depressed (n=26) | 32.35 (11.31) | 26 (100) | Not reported | fMRI | Resting state (ALFF) | NC>BD, BD>NC (Table 2) |
| Liu et al. 2013(69) | 21 BD, 21 UD | Depressed (n=21) | 31.05 (8.46) | 19 (90) | Not reported | fMRI | Resting state (ReHo) | BD>UD, UD>BD (Table 2) |
| Liu et al. 2016(191) | 28 BD I, 69 NC | Depressed (n=28) | 32.87 (10.45)^[[15]](#footnote-15)^ | 28 (100) | Not reported | fMRI | Resting state (ALFF, fALFF) | Main effect of group for ALFF, main effect of group for fALFF (Table 3) |
| Lui et al. 2015(42) | 57 BD I, 59 NC | Not specified (n=57) | 39 (68.4) | 57 (100) | 57 (100) with history | fMRI | Resting state (seed-to-voxel: L/R ACC, pre/ postcentral gyrus, thalamus, parahippocampus) | BD>NC L pre/postcentral, R thalamus, L ACC; NC>BD R parahippocampus (Table 3) |
| Luo et al. 2018(192) | 94 BD II, 100 NC | Depressed (n=94) | 27.18 (9.15) | 94 (0) | Not reported | fMRI | Resting state (seed-to-voxel: cerebellum) | NC>BD (Table 2) |
| Magioncalda et al. 2015(193) | 40 BD I, 40 NC | Hypo/manic (n=11); Depressed (n=11); Mixed (n=7); Euthymic (n=11) | 44.6 (11.8) | 39 (97) | Not reported | fMRI | Resting state (seed-to-voxel: perigenual ACC, subgenual ACC, PCC) | NC>BD (Table 2), NC>BD (Table 3), NC>BD (Table 4) |
| Mah et al. 2007(43) | 13 BD II, 18 NC | Depressed (n=13) | 43 (8.4) | 13 (100) | No current features | FDG PET | Resting state | BD>NC (Table 3) |
| **Publication** | **Analyzed Participant Groups, N** | **Mood State(s)** | **BD Mean Age (SD)** | **Medication Status**  **No. (%)** | **Psychosis**  **No. (%)** | **Imaging Modality** | **Design/Paradigm** | **Contrast(s) Used** |
| Maïza et al. 2010(194) | 8 BD, 16 NC, 16 SZ | Euthymic (n=8) | 41.5 (8.3) | 8 (100) | Not reported | fMRI | Listening to a story requiring social cognition (C) | Main effect of group (Fig. 1) |
| Malhi et al. 2005(44) | 12 BD I, 12 NC | Euthymic (n=12) | 34.9 (9.1) | 8 (67) | Not reported | fMRI | Emotional Stroop with negative, positive, and neutral words (EC) | Main effect of group for affect (Table 5) |
| Malhi et al. 2007(109) | 10 BD I, 10 NC | Euthymic (n=10) | 33.5 (8.7) | 7 (70) | Not reported | fMRI | Explicit facial affect recognition with fearful, disgusted, and neutral faces (E) | Main effect of group for disgust, main effect of group for fear (Table 4) |
| Malhi et al. 2007(45) | 10 BD I, 10 NC | Euthymic (n=10) | 32.4 (10.8) | 7 (70) | Not reported | fMRI | Implicit affect induction via Sternberg memory task (EC) | Main effect of group for negative affect (Table 5), main effect of group for positive affect (Table 6) |
| Malhi et al. 2008(195) | 20 BD I, 20 NC | Euthymic (n=20) | 35.3 (9.4) | 13 (65) | Not reported | fMRI | Theory of Mind (C) | Main effect of group (Table 3) |
| Man et al. 2019(46) | 18 BD I, 13 MDD | Hypo/manic (n=7); Depressed (n=11) | 40.28 (12.45)^[[16]](#footnote-16)^ | 18 (100) | Not reported | fMRI | Viewing negative, positive, and neutral images (E) (seed-based task-related beta-series correlation using L/R amygdala) | Group x condition x amygdala beta value int. (2: L and R amygdala, Table 2) |
| Manelis et al. 2016(47)* | 31 BD I, 36 NC, 39 MDD | Depressed (n=31) | 33.38 (8.44) | 31 (100) | No current features | fMRI | Card-guessing with monetary rewards (E) | BD>NC/NC>BD/BD>MDD/ MDD>BD during guess, receipt of feedback, anticipation win vs. rest, anticipation loss vs. rest |
| Marchand et al. 2007(48) | 14 BD I, 15 NC | Depressed (n=14) | 45.6 (11.7) | 13 (93) | 6 (43) with history | fMRI | Paced motor activation task (C) | BD>NC (Table 3) |
| Marchand et al. 2011(87) | 16 BD II, 19 NC | Depressed (n=16) | 32.9 (7.5) | 16 (0) | Not reported | fMRI | Paced motor activation task (C) (seed-to-voxel: putamen) | NC>BD, BD>NC (Table 3) |
| **Publication** | **Analyzed Participant Groups, N** | **Mood State(s)** | **BD Mean Age (SD)** | **Medication Status**  **No. (%)** | **Psychosis**  **No. (%)** | **Imaging Modality** | **Design/Paradigm** | **Contrast(s) Used** |
| Marchand et al. 2011(196) | 16 BD II, 19 NC | Depressed (n=16) | 32.9 (7.5) | 16 (0) | Not reported | fMRI | Facial affect recognition with happy faces (E) | NC>BD (Table 3, happy>neutral) |
| Marotta et al. 2019(197) | 90 BD, 27 NC | Euthymic (n=90) | 40.29 (11.4)^[[17]](#footnote-17)^ | 90 (100) | 50 (55) with history | FDG PET | Resting state | Main effect of group (Table 2) |
| Martino et al. 2016(198) | 61 BD I, 42 NC | Hypo/manic (n=21); Depressed (n=20); Euthymic (n=20) | 44.6 (11.1) | 59 (97) | Not reported | fMRI | Resting state (seed-to-voxel: perigenual ACC) | Main effect of group (Table S3a BD vs NC) |
| Mason et al. 2014(199)* | 20 BD I/II, 20 NC | Euthymic (n=20) | 35.95 (8.34) | 16 (80) | Not reported | fMRI | Risky decision-making roulette (E) | BD>NC, NC>BD anticipation + outcome |
| McIntosh et al. 2008(49) | 42 BD I, 37 NC, 27 SZ | Not specified (n=42) | 39.3 (10.8) | 42 (100) | 42 (100) with current features | fMRI | Hayling Sentence Completion Test (C) | Main effect of group (parametric analyses) |
| McKenna et al. 2014(200) | 23 BD I, 23 NC | Euthymic (n=23) | 45.31 (9.45) | 23 (100) | No current features | fMRI | Delayed match-to-sample (C) | Main effect of group for encode, main effect of group for maintenance (Table 2) |
| Mechelli et al. 2008(127) | 29 BD I, 45 NC, 41 SZ | Not specified (n=29) | 37.79 (11.34)^[[18]](#footnote-18)^ | 22 (76) | 25 (86) with history | fMRI | Verbal fluency (C) | Genotype x group interaction (high> low-risk, Table 3 and Fig. 3) |
| Mechelli et al. 2012(201) | 33 BD I, 47 NC | Not specified (n=33) | 38.79 (12.24) | 20 (61) | Not reported | fMRI | Verbal fluency (C) | G72 x DAOO x diagnostic group interaction (BD vs NC) |
| Mitchell et al. 2004(110) | 11 BD, 13 NC, 12 SZ | Not specified (n=11) | 42.8 (1.8) | 11 (100) | Not reported | fMRI | Emotional prosody (E) | SZ>BD/BD>SZ pure, SZ>BD/ BD>SZ unfiltered (Table 5); NC>BD pure, NC>BD/ BD>NC unfiltered (Table 6) |
| Monks et al. 2004(95) | 12 BD I, 12 NC | Euthymic (n=12) | 45.83 (9.56) | 12 (100) | Not reported | fMRI | N-back (C) | Main effect of group (Table 2) |
| **Publication** | **Analyzed Participant Groups, N** | **Mood State(s)** | **BD Mean Age (SD)** | **Medication Status**  **No. (%)** | **Psychosis**  **No. (%)** | **Imaging Modality** | **Design/Paradigm** | **Contrast(s) Used** |
| Morris et al. 2012(118)* | 13 BD I, 15 NC, 12 SZ | Euthymic (n=6); Hypo/manic (n=5); Not specified (n=2) | 41 (3) | 13 (100) | 13 (100) with current features | fMRI | Emotion regulation of negative affect (E) | NC>BD/BD>NC downregulation, NC>BD/BD>NC upregulation, SZ>BD/BD>SZ downregulation, SZ>BD/BD<SZ upregulation |
| Mullin et al. 2012(202) | 22 BD I, 19 NC | Euthymic (n=22) | 31.68 (8.96) | 22 (100) | 9 (41) with history | fMRI | Emotional Faces N-back with fearful, neutral, and happy face distractors (E) (granger causality mapping using amygdala and DLPFC) | Main effect of group for 2-back fearful faces and main effect of group for 2-back happy faces (amygdala, Fig. 4); main effect of group for 2-back fearful faces and main effect of group for 2-back happy faces (DLPFC, Fig. S1) |
| Negoias et al. 2019(50) | 11 BD I/II, 11 NC | Euthymic (n=11) | 36 (7.39) | Not reported | Not reported | fMRI | Rating intensity and hedonic value of olfactory stimuli (E) | BD>NC, NC>BD (Table 3); BD>NC, NC>BD (Table 4); BD>NC, NC>BD (Table 5) |
| Nusslock et al. 2012(203) | 21 BD I, 20 NC | Euthymic (n=21) | 31.53 (8.66) | 20 (95) | No current features | fMRI | Card guessing with monetary rewards (E) | Group x anticipation int. (Table 3) |
| Oertel-Knöchel et al. 2013(51) | 21 BD I, 20 NC | Euthymic (n=21) | 35.67 (10.68) | 21 (100) | Not reported | fMRI | Verbal learning and recognition (C) | NC>BD encoding, NC>BD retrieval (Table 3) |
| Oertel-Knöchel et al. 2014(204) | 21 BD I, 20 NC | Euthymic (n=21) | 35.67 (10.68) | 21 (100) | Not reported | fMRI | Non-verbal learning and recognition (C) | NC>BD encoding, BD>NC retrieval (Table 2) |
| Oertel-Knöchel et al. 2015(205) | 21 BD I, 20 NC | Euthymic (n=21) | 35.67 (10.68) | 21 (100) | Not reported | fMRI | Resting state (seed-to-voxel: inferior frontal gyrus, middle/ superior frontal gyrus) | Main effect of group for L middle/superior frontal gyrus, main effect of group for L inferior frontal gyrus (Table 2) |
| Palaniyappan & Liddle 2014(98) | 20 BD, 34 NC, 39 SZ | Not specified (n=20) | 35.25 (10.8) | 19 (95) | 20 (100) with current features | fMRI | 2: N-back (C); Resting state (degree centrality) | NC>BD, BD>NC, BD>SZ, SZ>BD (Table 2, N-back); NC>BD, BD>NC, BD>SZ, SZ>BD (Table S2, rest) |
| **Publication** | **Analyzed Participant Groups, N** | **Mood State(s)** | **BD Mean Age (SD)** | **Medication Status**  **No. (%)** | **Psychosis**  **No. (%)** | **Imaging Modality** | **Design/Paradigm** | **Contrast(s) Used** |
| Palaniyappan et al. 2019(206) | 22 BD, 34 SZ | Not specified (n=22) | 34.6 (10.4) | 21 (95) | 22 (100) with current features | fMRI | Resting state (degree centrality) | BD>SZ, SZ>BD (Table S3) |
| Pang et al. 2018(25) | 30 BD, 30 NC, 30 MDD | Depressed (n=30) | 35.13 (9.25) | 23 (77)^[[19]](#footnote-19)^ | Not reported | fMRI | Resting state (static and dynamic FC: R ventral and posterior dorsal anterior insula) | Main effect of group for static FC (1 seed, table 2); 2 main effects of group for dynamic FC (2 seeds, table 3) |
| Penfold et al. 2015(207) | 19 BD II, 20 NC | Depressed (n=19) | 36.3 (12.2) | 19 (0) | Not reported | fMRI | Go/NoGo (C) | NC>BD (Table 3) |
| Perlman et al. 2012(111) | 31 BD I, 25 NC | Euthymic (n=31) | 32.63 (8.21) | 31 (100) | No current features | fMRI | Implicit viewing of angry, fearful, sad and happy faces (E) | Group x task int. (all faces/shapes – exploratory whole-brain analysis) |
| Poletti et al. 2017(135)* | 25 BD I, 21 NC | Depressed (n=25) | 47.44 (12.99) | 8 (32) | No current features | fMRI | Emotional Go/NoGo with positive and negative words (EC) | NC>BD negative (NoGo-Go) minus positive (NoGo-Go) |
| Pomarol-Clotet et al. 2012(104) | 29 BD, 46 NC | Hypo/manic (n=29) | 40.79 (12.08) | 29 (0) | 29 (100) with current features | fMRI | N-back (C) | NC>BD, BD>NC (Table 2) |
| Pomarol-Clotet et al. 2015(52) | 114 BD I/II, 38 NC | Hypo/manic (n=38); Depressed (n=38); Euthymic (n=38) | 39.88 (10.18)^[[20]](#footnote-20)^ | 114 (100) | 84 (73.68) with history | fMRI | N-back (C) | NC>BD (manic), BD>NC (manic), NC>BD (depressed), BD>NC (depressed), BD>NC (euthymic) |
| Pompei et al. 2011(53) | 39 BD I, 48 NC, 25 NCR, 14 MDD Relatives | Euthymic (n=39) | 39.43 (11.5) | 30 (77) | Some with history (% not reported – taken from the VIBES cohort(164) | fMRI | Stroop (C) (PPI: R VLPFC) | Main effect of group (Table 2) |
| **Publication** | **Analyzed Participant Groups, N** | **Mood State(s)** | **BD Mean Age (SD)** | **Medication Status**  **No. (%)** | **Psychosis**  **No. (%)** | **Imaging Modality** | **Design/Paradigm** | **Contrast(s) Used** |
| Pompei et al. 2011(88) | 39 BD I, 48 NC, 25 NCR, 14 MDD Relatives | Euthymic (n=39) | 39.43 (11.5) | 30 (77) | Some with history (% not reported – taken from the VIBES cohort(164) | fMRI | Stroop (C) | Main effect of group (Results/Figure 1) |
| Qiu et al. 2018(76) | 28 BD, 27 NC, 47 UD | Depressed (n=28) | 31.79 (12.83) | 23 (82) | Not reported | fMRI | Resting state (fALFF) | Main effect of group (Table 2) |
| Qiu et al. 2019(208) | 100 BD II, 100 NC | Depressed (n=100) | 26.37 (8.89) | 100 (0) | Not reported | fMRI | Resting state (ReHo) | NC>BD, BD>NC (Table 2) |
| Reavis et al. 2017(89)* | 51 BD, 47 NC, 50 SZ | Not specified (n=51) | 44.78 (12.41) | 51 (100) | Not reported | fMRI | Object perception (C) (MVPA searchlight) | NC>BD, BD>SZ |
| Redlich et al. 2015(54) | 33 BD I, 34 NC, 33 UD | Depressed (n=33) | 38.1 (12.6) | 32 (97) | Not reported | fMRI | Card guessing with monetary rewards (E) | Group x condition (reward>control) int. (Table 3 ANOVA) |
| Reinke et al. 2013(209) | 21 BD, 20 NC | Euthymic (n=21) | 35.67 (10.68) | 21 (100) | Not reported | fMRI | Resting state (seed-to-voxel: Heschl’s Gyrus, planum temporale) | NC>BD Heschl’s Gyrus, NC>BD and BD>NC planum temporale (Table 2) |
| Rey et al. 2014(63)^,^^[[21]](#footnote-21)^ | 12 BD NOS/I/II, 12 NC | Hypo/manic (n=9); Depressed (n=9); Euthymic (n=11) | 42.6 (11.4) | 12 (100) | Not reported | fMRI | Emotional face-word interference with joyful and fearful faces (EC) | Table 2 all contrasts (n=10), Table S7 group x emotion (fear>joy) interactions (n=2) |
| Rive et al. 2015(210) | 26 BD I/II, 21 MDD | Euthymic (n=26) | 42.7 (10.7) | 26 (0) | Not reported | fMRI | Emotion regulation of sad, happy, fearful, and neutral pictures (E) | Remitted BD > remitted MDD, across emotions (Table 2) |
| **Publication** | **Analyzed Participant Groups, N** | **Mood State(s)** | **BD Mean Age (SD)** | **Medication Status**  **No. (%)** | **Psychosis**  **No. (%)** | **Imaging Modality** | **Design/Paradigm** | **Contrast(s) Used** |
| Rive et al. 2016(211) | 32 BD I/II, 35 NC, 40 MDD | Depressed (n=9); Euthymic (n=23) | 40.58 (10.77)^[[22]](#footnote-22)^ | 32 (0) | Not reported | fMRI | Tower of London (C) | Main effect of group (Table 2, */**regions) |
| Robinson et al. 2009(81) | 15 BD I, 15 NC | Euthymic (n=15) | 39 (12.61) | 14 (93) | Not reported | fMRI | Delayed non-match-to-sample (C) | NC>BD and BD>NC familiarity, NC>BD and BD>NC novelty (Table 2) |
| Rodríguez-Cano et al. 2017(82) | 26 BD I, 26 NC | Depressed (n=26) | 45.58 (9.23) | 26 (100) | 17 (65) with history | fMRI | N-back (C) | BD>NC 1-back vs. BL, BD>NC 2-back vs. BL, NC>BD 2-back vs. BL (Table 2) |
| Rosenfeld et al. 2014(212)* | 17 BD I, 19 NC | Euthymic (n=17) | 33.9 (11)^[[23]](#footnote-23)^ | 16 (94)^[[24]](#footnote-24)^ | No history | fMRI | Gender discrimination with happy, angry, and neutral faces (E) | BD>NC/ NC>BD happy, BD>NC/ NC>BD angry, BD>NC/NC>BD neutral |
| Roth et al. 2006(213) | 11 BD I, 11 NC | Mixed (n=4); Hypo/manic (n=1); Depressed (n=1); Euthymic (n=5) | 37.7 (14.4) | 11 (100) | 5 (45) with current features | fMRI | Stroop (C) | NC>BD (Table 2) |
| Ryan et al. 2015(214) | 16 BD I/II/NOS, 17 NC, 19 MDD | Not specified (n=16) | 44.1 (11.5) | Not reported | Not reported | fMRI | Go/NoGo (C) | Main effect of group (Table 2) |
| Sepede et al. 2015(136) | 23 BD I, 24 NC, 22 NCR | Euthymic (n=23) | 35.2 (7.9) | 19 (83) | 22 (96) with history | fMRI | Emotion processing of negative and neutral pictures (E) | Main effect of group (Table 3), between group x negative valence condition int. (Table 3) |
| **Publication** | **Analyzed Participant Groups, N** | **Mood State(s)** | **BD Mean Age (SD)** | **Medication Status**  **No. (%)** | **Psychosis**  **No. (%)** | **Imaging Modality** | **Design/Paradigm** | **Contrast(s) Used** |
| Shaffer et al. 2018(55) | 31 BD I, 32 NC | Depressed (n=15); Hypo/ manic (n=16) | 42.25 (13.45)^[[25]](#footnote-25)^ | 31 (100) | Not reported | fMRI | Flashing checkerboard (C) | NC>depressed BD (Table 2); NC>manic BD (Table 2) |
| Sharma et al. 2016(56) | 24 BD I/II, 24 MDD | Depressed (n=24) | 38 (11.7) | 24 (100) | Not reported | fMRI | Social reward task with happy and angry facial feedback (E) | Group x depression severity int. for happy>angry (Table S4) |
| Skåtun et al. 2016(215) | 43 BD I/II/NOS, 196 NC, 71 SZ | Not specified (n=43) | 31.3 (11.3) | 43 (100)^[[26]](#footnote-26)^ | 43 (100) with current features | fMRI | Resting state (global FC eigenvector centrality mapping) | Main effect of group on centrality (Table 2) |
| Strakowski et al. 2005(216) | 16 BD I, 16 NC | Euthymic (n=16) | 28 (7) | 8 (50) | Not reported | fMRI | Stroop (C) | NC>BD, BD>NC (Table 2) |
| Strakowski et al. 2011(217) | 40 BD I, 36 NC | Hypo/manic (n=25); Mixed (n=15) | 30 (10) | 31 (78) | Not reported | fMRI | CPT with emotional and neutral distracters (CPT-END) (EC) | Group x cue int. (Table 3) |
| Sugihara et al. 2017(99) | 45 BD I/II, 48 NC, 31 NCR | Not specified (n=45) | 40.95 (12.5)^[[27]](#footnote-27)^ | 45 (100) | 28 (62) with history | fMRI | N-back (C) | Main effect of group (Table 2, all participants) |
| Surguladze et al. 2010(218) | 20 BD I, 20 NC, 20 NCR | Euthymic (n=20) | 42.7 (10.4) | 16 (80) | 20 (100) with history | fMRI | Gender discrimination with fearful, neutral, and happy faces (E) | Group x emotion (fear and happy) x intensity int. (Results, Figs. 1-2) |
| Syan et al. 2018(219) | 40 BD I/II (19 with PMDD), 25 NC, 20 PMDD | Euthymic (n=40) | 32.7 (7.98)^[[28]](#footnote-28)^ | 40 (100) | 10 (25) with history | fMRI | Resting state (seed-to-voxel: hippocampus) | Main effect of group (Table 4, R hippocampus) |
| **Publication** | **Analyzed Participant Groups, N** | **Mood State(s)** | **BD Mean Age (SD)** | **Medication Status**  **No. (%)** | **Psychosis**  **No. (%)** | **Imaging Modality** | **Design/Paradigm** | **Contrast(s) Used** |
| Taylor Tavares et al. 2008(90) | 12 BD, 13 MDD | Depressed (n=12) | 33.4 (3.16) | 12 (0) | Not reported | fMRI | Probabilistic reversal learning with negative feedback (C) | MDD>BD error switches, BD>MDD error switches, BD>MDD error switches–error non-switches, BD>MDD reversal (Table 4) |
| Tecelão et al. 2018(220) | 40 BD, 80 NC, 54 SZ | Not specified (n=40) | 40 (12) | 40 (100) | 30 (75) with history | fMRI | Verbal fluency (C) | ZNF804A genotype x diagnosis int. (Table 1) |
| Tecelão et al. 2019(57) | 40 BD, 80 NC, 54 SZ | Not specified (n=40) | 40 (12) | 40 (100) | 30 (75) with history | fMRI | Verbal fluency (C) | CACNA1C genotype x diagnosis int. (Table 1, 1.1 BD>NC), CACNA1C x ZNF804A genotype int. (Table 1, 1.2 BD>NC, BD>SZ) |
| Tesli et al. 2015(221) | 85 BD I/II/NOS, 121 NC | Euthymic (n=26); Depressed (n=34); Not specified (n=25) | 34.8 (11.2) | 85 (100) | 38 (45) with current features^[[29]](#footnote-29)^ | fMRI | Face-matching with angry, fearful, and happy faces (E) | Table 2 all case-control analyses |
| Torrisi et al. 2013(222) | 20 BD I, 20 NC | Euthymic (n=20) | 42.1 (11.4) | 17 (85) | Not reported | fMRI | Resting state (seed-to-voxel: R amygdala) | BD>NC R amygdala connectivity (Table 3) |
| Townsend et al. 2013(83) | 32 BD I (16 with ADHD), 30 NC, 16 ADHD | Euthymic (n=32) | 37.25 (13.15)^[[30]](#footnote-30)^ | 23 (72) | Not reported | fMRI | Go/NoGo (C) | Table 2 ANOVA |
| Townsend et al. 2013(58) | 30 BD I, 26 NC | Euthymic (n=30) | 37.9 (12.6) | 21 (70) | Not reported | fMRI | Emotion regulation with negative and neutral pictures (E) (PPI: amygdala) | NC>BD (Table 2), NC>BD (Table 3, PPI) |
| **Publication** | **Analyzed Participant Groups, N** | **Mood State(s)** | **BD Mean Age (SD)** | **Medication Status**  **No. (%)** | **Psychosis**  **No. (%)** | **Imaging Modality** | **Design/Paradigm** | **Contrast(s) Used** |
| Trost et al. 2014(122) | 16 BD I, 16 NC | Euthymic (n=7); Depressed (n=9) | 35.6 (9.2) | 14 (87) | Not reported | fMRI | Desire-reason dilemma reward paradigm (E) | NC>BD (Table 2), NC>BD (Table 3) |
| Tseng et al. 2016(223) | 14 BD I/II, 14 NC | Euthymic (n=8); Depressed (n=5) | 37.94 (9.98) | 12 (86) | Not reported | fMRI | Affective priming with angry, happy, and neutral faces (E) (PPI: amygdala) | Diagnosis x all emotion types int. (Results 3.3.2), diagnosis x awareness int. (Table 2), main effect of diagnosis (Table 2) |
| Usnich et al. 2015(224) | 14 BD I, 14 NC | Hypo/manic (n=14) | 33.4 (10.4) | 14 (100) | Not reported | fMRI | Viewing social and non-social IAPS pictures followed by memory retrieval task (EC) | NC>BD, BD>NC (Table S1) |
| Vai et al. 2015(112)* | 37 BD I, 35 NC | Depressed (n=37) | 48.05 (10.69) | 37 (100) | No current features | fMRI | Face-matching with fearful and angry faces (E) | NC>BD fearful + angry |
| Vargas et al. 2014(225) | 21 BD I, 12 NC | Euthymic (n=21) | 39.29 (5.52)^[[31]](#footnote-31)^ | 10 (48) | Not reported | fMRI | Resting state (ICA) | BD>NC (Table 2) |
| Vizueta et al. 2012(113) | 21 BD II, 21 NC | Depressed (n=21) | 38.4 (12.2) | 21 (0) | Not reported | fMRI | Face-matching with angry and fearful faces (E) (seed-to-voxel: amygdala) | NC>BD (Table 2), NC>BD (Table 3), BD>NC (Table 3) |
| Wang et al. 2009(114) | 33 BD, 31 NC | Hypo/manic /mixed (n=10); Depressed (n=7); Euthymic (n=16) | 31.8 (9.6) | 27 (82) | Not reported | fMRI | Gender discrimination with fearful, happy, and neutral faces (E) (seed-to-voxel: perigenual ACC) | NC>BD fear (Fig. 1), NC>BD happy (Fig. 1) |
| Wang et al. 2015(226) | 26 BD II, 40 NC | Depressed (n=26) | 26.12 (10.3) | 26 (0) | Not reported | fMRI | Resting state (VMHC) | NC>BD (Table 2) |
| Wang et al. 2016(70) | 37 BD II, 37 NC | Depressed (n=37) | 26.37 (8.51) | 37 (0) | Not reported | fMRI | Resting state (FCS) | NC>BD, BD>NC (Table 2) |
| **Publication** | **Analyzed Participant Groups, N** | **Mood State(s)** | **BD Mean Age (SD)** | **Medication Status**  **No. (%)** | **Psychosis**  **No. (%)** | **Imaging Modality** | **Design/Paradigm** | **Contrast(s) Used** |
| Wang et al. 2017(71) | 48 BD II, 51 NC, 48 MDD | Depressed (n=48) | 27.33 (8.41) | 48 (0) | Not reported | fMRI | Resting state (FCS) | Main effect of group for long-range FCS (Table 2); main effect of group for short-range FCS (Table 2) |
| Wang et al. 2018(227) | 25 BD II, 25 NC | Euthymic (n=25) | 28.55 (9.76) | 20 (80) | Not reported | fMRI | Resting state (FCS and seed-to-voxel: R/L cerebellum) | NC>BD FCS, NC>BD R cerebellum FC, NC>BD L cerebellum FC (Table 2) |
| Wang et al. 2019(228) | 30 BD, 31 NC | Depressed (n=30) | 36.3 (11.01) | 30 (0) | Not reported | fMRI | Resting state (fALFF) | NC>BD, BD>NC (Table 3) |
| Wang et al. 2020(72) | 38 BD II, 47 NC, 35 MDD | Depressed (n=38) | 25.84 (9.01) | 38 (0) | Not reported | fMRI | Resting state (ICA: anterior/posterior DMN, central executive networks) | 3 main effects of group (for each of the 3 networks, Table 3) |
| Wessa et al. 2007(229) | 17 BD I/II, 17 NC | Euthymic (n=17) | 44.94 (12.7) | 15 (88) | Not reported | fMRI | Emotional Go/ NoGo with fearful, happy, and neutral face distractors (EC) | BD>NC emotional go/nogo vs. control go/nogo; BD>NC emotional distractors vs. neutral distractors (Table 2) |
| Whittaker et al. 2018(230) | 35 BD I/II, 23 NC | Euthymic (n=35) | 44.71 (5.51) | 31 (89) | No history | fMRI | Resting state (seed-to-voxel: nucleus accumbens) | BD>NC (Fig. 2A) |
| Willert et al. 2015(124)* | 24 BD I, 24 NC, 21 NCR | Euthymic (n=24) | 44.75 (12.21) | 24 (100) | Not reported | fMRI | Affective Theory of Mind (E) | NC>BD, BD>NCR, NCR>BD during mentalizing>non-mentalizing control |
| Xu et al. 2014(59) | 29 BD, 29 NC | Depressed (n=5); Hypo/ manic/mixed (n=6); Euthymic (n=18) | 30.52 (8.8) | 24 (83) | Not reported | fMRI | Resting state (ALFF) | BD>NC, NC>BD (Table 2) |
| Yang et al. 2019(73) | 41 BD, 93 NC | Not specified (n=41) | 32.98 (9.41) | 41 (100) | Not reported | fMRI | Resting state (fALFF) | NC>BD, BD>NC (Table S1) |
| Yin et al. 2018(231) | 21 BD, 70 NC, 40 MDD | Depressed (n=21) | 29.29 (8.35) | 9 (43) | Not reported | fMRI | Resting state (seed-to-voxel: ventral and dorsal anterior and posterior insula) | 3 main effects of group (for each of the 3 insula seeds; Table 2) |
| **Publication** | **Analyzed Participant Groups, N** | **Mood State(s)** | **BD Mean Age (SD)** | **Medication Status**  **No. (%)** | **Psychosis**  **No. (%)** | **Imaging Modality** | **Design/Paradigm** | **Contrast(s) Used** |
| Yip et al. 2014(232) | 15 BD II, 20 NC | Euthymic (n=15) | 23.07 (3.73) | 15 (0) | Not reported | fMRI | Resting state (ICA) | BD>NC (Table 2) |
| Yip et al. 2015(115)* | 20 BD II/NOS, 20 NC | Euthymic (n=20) | 22.59 (0.9) | 20 (0) | Not reported | fMRI | Monetary incentive delay (E) | NC>BD 1^st^ anticipation phase: wins, NC>BD 1^st^ anticipation phase: losses, NC>BD 2^nd^ anticipation phase: wins, NC>BD 2^nd^ anticipation phase: losses, NC>BD win outcome, NC> BD loss outcome, NC>BD 1^st^ anticipation phase: wins>neutral, NC>BD 2^nd^ anticipation phase: wins>neutral, NC>BD 2^nd^ anticipation phase: losses>neutral, NC>BD outcome phase: wins>neutral, NC>BD outcome phase: losses> neutral, BD>NC 1^st^ anticipation phase: losses> neutral |
| Yoshimura et al. 2014(100) | 10 BD I, 10 NC | Euthymic (n=10) | 48.4 (8.8) | 10 (100) | Not reported | fMRI | Verbal fluency (C) | BD>NC (Results) |
| Young et al. 2016(116) | 16 BD I, 16 NC, 16 MDD | Depressed (n=16) | 37.6 (9.32) | 16 (0) | Not reported | fMRI | Autobiographical memory recall with positive and negative cue words (EC) | Main effect of group for positive memories, main effect of group for negative memories (Table 3) |
| Yu et al. 2017(233) | 13 BD I, 15 NC, 15 UD | Depressed (n=13) | 31.2 (10.5) | Not reported | Not reported | fMRI | Resting state (fALFF) | Main effect of group (Table 2) |
| Zhang et al. 2016(60) | 18 BD I/II, 21 NC | Euthymic (n=14); Depressed (n=4) | 40.22 (12.34) | 17 (94) | 18 (100) with history | fMRI | Guided self-reflection (E) (PPI: ACC, insula, VMPFC) | NC>BD ACC, NC>BD insula, NC>BD VMPFC (Table 3) |
| Zhang et al. 2017(234) | 14 BD, 14 NC, 14 MDD | Depressed (n=14) | 33.79 (11.08) | 14 (0) | Not reported | fMRI | Resting state (fALFF) | Main effect of group (Table 2) |
| **Publication** | **Analyzed Participant Groups, N** | **Mood State(s)** | **BD Mean Age (SD)** | **Medication Status**  **No. (%)** | **Psychosis**  **No. (%)** | **Imaging Modality** | **Design/Paradigm** | **Contrast(s) Used** |
| Zhang et al. 2019(64)* | 99 BD I, 64 NC | Hypo/manic (n=30); Depressed (n=35); Euthymic (n=34) | 45.7 (10) | 94 (95) | Not reported | fMRI | Resting state (ALFF) | Manic BD>NC, depressed BD>NC, euthymic BD>NC |
| Zhang et al. 2020(235) | 15 BD I/II, 15 NC | Euthymic (n=13); Depressed (n=2) | 39.87 (12.5) | 14 (93) | 15 (100) with history | fMRI | Cognitive reappraisal of negative and neutral pictures (E) (finite impulse response modeling) | BD vs. NC main effect of group (Table 2) |
| Zhao et al. 2017(236) | 20 BD I/II, 38 NC | Euthymic (n=20) | 28.7 (10.18) | 16 (80) | Not reported | fMRI | Resting state (VMHC) | NC>BD (Table 2) |
| Zhong et al. 2019(74) | 90 BD II, 100 NC | Depressed (n=90) | 26.74 (8.73) | 90 (0) | Not reported | fMRI | Resting state (ALFF) | NC>BD (Table 2) |
| Zhou et al. 2017(237) | 52 BD I, 70 NC | Euthymic (n=24); Depressed (n=16);  Hypo/manic  (n=12) | 29.84 (8.97) | 42 (81) | 3 (6) with history | fMRI | Resting state (degree centrality) | NC>BD, BD>NC (Table 2) |

Abbreviations: ACC = Anterior Cingulate Cortex; ADHD = Attention Deficit Hyperactivity Disorder; ALFF = Amplitude Low-Frequency Fluctuation; ASL = Arterial Spin Labeling; BD = Bipolar Disorder; BL = Baseline; BPD = Borderline Personality Disorder; C=Cognitive; CBF = Cerebral Blood Flow; CPT = Continuous Performance Task; DLPFC = Dorsolateral Prefrontal Cortex; DMN = Default Mode Network; E=Emotional; EC=Cognitive Task With Emotional Manipulation; fALFF = Fractional Amplitude Low-Frequency Fluctuation; FC = Functional Connectivity; FCS = Functional Connectivity Strength; FDG = Fluorodeoxyglucose; Fig. = Figure; fMRI = Functional Magnetic Resonance Imaging; IAPS = International Affective Picture System; ICA = Independent Component Analysis; int. = interaction; L = Left; MDD = Major Depressive Disorder; MPFC = Medial Prefrontal Cortex; N = Sample Size; NC = Non-Clinical Controls; NCR = Non-Clinical Relatives; No. = Number; [O]H_2_O = Oxygen-Water; PCC = Posterior Cingulate Cortex; PET = Positron Emission Tomography; PMDD = Premenstrual Dysphoric Disorder; PPI = Psychophysiological Interaction; R = Right; ReHo = Regional Homogeneity; ROI = Region of Interest; SD = Standard Deviation; SZ = Schizophrenia; UD = Unipolar Depression; VIBES = Vulnerability to Bipolar Disorders Study; VMHC = Voxel-Mirrored Homotopic Connectivity; VMPFC = Ventromedial Prefrontal Cortex; *Unpublished data sent by study authors.

**Supplementary References**

1. Moher D, Liberati A, Tetzlaff J, Altman DG, Group TP. Preferred Reporting Items for Systematic Reviews and Meta-Analyses: The PRISMA Statement. PLOS Medicine. 2009 Jul 21;6(7):e1000097.

2. White J. PubMed 2.0. Medical Reference Services Quarterly. 2020 Oct 1;39(4):382–7.

3. Ladouceur CD, Peper JS, Crone EA, Dahl RE. White matter development in adolescence: The influence of puberty and implications for affective disorders. Developmental Cognitive Neuroscience. 2012 Jan 1;2(1):36–54.

4. Dai J, Scherf KS. Puberty and functional brain development in humans: Convergence in findings? Developmental Cognitive Neuroscience. 2019 Oct 1;39:100690.

5. Goddings AL, Beltz A, Peper JS, Crone EA, Braams BR. Understanding the Role of Puberty in Structural and Functional Development of the Adolescent Brain. Journal of Research on Adolescence. 2019;29(1):32–53.

6. Sisk CL, Zehr JL. Pubertal hormones organize the adolescent brain and behavior. Frontiers in Neuroendocrinology. 2005 Oct 1;26(3):163–74.

7. Peper JS, Dahl RE. The Teenage Brain: Surging Hormones—Brain-Behavior Interactions During Puberty. Curr Dir Psychol Sci. 2013 Apr 1;22(2):134–9.

8. Savio A, Fünger S, Tahmasian M, Rachakonda S, Manoliu A, Sorg C, et al. Resting-State Networks as Simultaneously Measured with Functional MRI and PET. Journal of Nuclear Medicine. 2017 Aug 1;58(8):1314–7.

9. Ramsey NF, Kirkby BS, Van Gelderen P, Berman KF, Duyn JH, Frank JA, et al. Functional Mapping of Human Sensorimotor Cortex with 3D BOLD fMRI Correlates Highly with H215O PET rCBF. J Cereb Blood Flow Metab. 1996 Sep 1;16(5):755–64.

10. Chen Y, Wolk DA, Reddin JS, Korczykowski M, Martinez PM, Musiek ES, et al. Voxel-level comparison of arterial spin-labeled perfusion MRI and FDG-PET in Alzheimer disease. Neurology. 2011 Nov 29;77(22):1977–85.

11. Liebenthal E, Desai RH, Humphries C, Sabri M, Desai A. The functional organization of the left STS: a large scale meta-analysis of PET and fMRI studies of healthy adults. Front Neurosci [Internet]. 2014 [cited 2021 Jan 3];8. Available from: https://www.frontiersin.org/articles/10.3389/fnins.2014.00289/full

12. Phan KL, Wager T, Taylor SF, Liberzon I. Functional Neuroanatomy of Emotion: A Meta-Analysis of Emotion Activation Studies in PET and fMRI. NeuroImage. 2002 Jun 1;16(2):331–48.

13. Müller VI, Cieslik EC, Serbanescu I, Laird AR, Fox PT, Eickhoff SB. Altered Brain Activity in Unipolar Depression Revisited: Meta-analyses of Neuroimaging Studies. JAMA Psychiatry. 2017 Jan 1;74(1):47–55.

14. Engelmann JM, Versace F, Robinson JD, Minnix JA, Lam CY, Cui Y, et al. Neural substrates of smoking cue reactivity: A meta-analysis of fMRI studies. Neuroimage. 2012 Mar;60(1):252–62.

15. Diener C, Kuehner C, Brusniak W, Ubl B, Wessa M, Flor H. A meta-analysis of neurofunctional imaging studies of emotion and cognition in major depression. NeuroImage. 2012 Jul 2;61(3):677–85.

16. Eickhoff SB, Laird AR, Grefkes C, Wang LE, Zilles K, Fox PT. Coordinate‐based activation likelihood estimation meta‐analysis of neuroimaging data: A random‐effects approach based on empirical estimates of spatial uncertainty. Hum Brain Mapp. 2009 Jan 26;30(9):2907–26.

17. Reddan MC, Lindquist MA, Wager TD. Effect Size Estimation in Neuroimaging. JAMA Psychiatry. 2017 Mar 1;74(3):207–8.

18. Lancaster JL, Tordesillas‐Gutiérrez D, Martinez M, Salinas F, Evans A, Zilles K, et al. Bias between MNI and Talairach coordinates analyzed using the ICBM-152 brain template. Human Brain Mapping. 2007;28(11):1194–205.

19. Laird AR, Robinson JL, McMillan KM, Tordesillas-Gutiérrez D, Moran ST, Gonzales SM, et al. Comparison of the disparity between Talairach and MNI coordinates in functional neuroimaging data: Validation of the Lancaster transform. NeuroImage. 2010 Jun 1;51(2):677–83.

20. Goebel R, Esposito F, Formisano E. Analysis of functional image analysis contest (FIAC) data with brainvoyager QX: From single-subject to cortically aligned group general linear model analysis and self-organizing group independent component analysis. Human Brain Mapping. 2006;27(5):392–401.

21. Pauli R, Bowring A, Reynolds R, Chen G, Nichols TE, Maumet C. Exploring fMRI Results Space: 31 Variants of an fMRI Analysis in AFNI, FSL, and SPM. Front Neuroinform [Internet]. 2016 [cited 2021 Jan 4];10. Available from: https://www.frontiersin.org/articles/10.3389/fninf.2016.00024/full#B6

22. McIntyre RS, Soczynska JK, Konarski J. Bipolar Disorder: Defining Remission and Selecting Treatment. Psychiatric Times. 2006 Oct 1;23(11):46–46.

23. Deckersbach T, Dougherty DD, Savage C, McMurrich S, Fischman AJ, Nierenberg A, et al. Impaired Recruitment of the Dorsolateral Prefrontal Cortex and Hippocampus During Encoding in Bipolar Disorder. Biological Psychiatry. 2006 Jan 15;59(2):138–46.

24. Corbalán F, Beaulieu S, Armony JL. Emotion regulation in bipolar disorder type I: an fMRI study. Psychological Medicine. 2015 Sep;45(12):2521–31.

25. Pang Y, Chen H, Wang Y, Long Z, He Z, Zhang H, et al. Transdiagnostic and diagnosis-specific dynamic functional connectivity anchored in the right anterior insula in major depressive disorder and bipolar depression. Progress in Neuro-Psychopharmacology and Biological Psychiatry. 2018 Jul 13;85:7–15.

26. Kay SR, Fiszbein A, Opler LA. The Positive and Negative Syndrome Scale (PANSS) for Schizophrenia. Schizophrenia Bulletin. 1987 Jan 1;13(2):261–76.

27. Adler CM, Holland SK, Schmithorst V, Tuchfarber MJ, Strakowski SM. Changes in neuronal activation in patients with bipolar disorder during performance of a working memory task. Bipolar Disorders. 2004;6(6):540–9.

28. Dutra SJ, Cunningham WA, Kober H, Gruber J. Elevated striatal reactivity across monetary and social rewards in bipolar I disorder. Journal of Abnormal Psychology. 2015;124(4):890–904.

29. MacKinnon DP, Krull JL, Lockwood CM. Equivalence of the Mediation, Confounding and Suppression Effect. Prev Sci. 2000 Dec;1(4):173.

30. Hafeman DM, Chang KD, Garrett AS, Sanders EM, Phillips ML. Effects of medication on neuroimaging findings in bipolar disorder: an updated review. Bipolar Disorders. 2012;14(4):375–410.

31. Eickhoff SB, Stephan KE, Mohlberg H, Grefkes C, Fink GR, Amunts K, et al. A new SPM toolbox for combining probabilistic cytoarchitectonic maps and functional imaging data. NeuroImage. 2005 May 1;25(4):1325–35.

32. Eickhoff SB, Bzdok D, Laird AR, Kurth F, Fox PT. Activation likelihood estimation meta-analysis revisited. NeuroImage. 2012 Feb 1;59(3):2349–61.

33. Hamilton JP, Etkin A, Furman DJ, Lemus MG, Johnson RF, Gotlib IH. Functional Neuroimaging of Major Depressive Disorder: A Meta-Analysis and New Integration of Baseline Activation and Neural Response Data. AJP. 2012 Jul 1;169(7):693–703.

34. Altinay MI, Hulvershorn LA, Karne H, Beall EB, Anand A. Differential Resting-State Functional Connectivity of Striatal Subregions in Bipolar Depression and Hypomania. Brain Connect. 2016 Apr 1;6(3):255–65.

35. Argyelan M, Ikuta T, DeRosse P, Braga RJ, Burdick KE, John M, et al. Resting-State fMRI Connectivity Impairment in Schizophrenia and Bipolar Disorder. Schizophr Bull. 2014 Jan 1;40(1):100–10.

36. Dutra SJ, Man V, Kober H, Cunningham WA, Gruber J. Disrupted cortico-limbic connectivity during reward processing in remitted bipolar I disorder. Bipolar Disorders. 2017;19(8):661–75.

37. Ellard KK, Zimmerman JP, Kaur N, Van Dijk KRA, Roffman JL, Nierenberg AA, et al. Functional Connectivity Between Anterior Insula and Key Nodes of Frontoparietal Executive Control and Salience Networks Distinguish Bipolar Depression From Unipolar Depression and Healthy Control Subjects. Biological Psychiatry: Cognitive Neuroscience and Neuroimaging. 2018 May 1;3(5):473–84.

38. Frangou S. Brain structural and functional correlates of resilience to Bipolar Disorder. Front Hum Neurosci [Internet]. 2012 [cited 2020 Dec 6];5. Available from: https://www.frontiersin.org/articles/10.3389/fnhum.2011.00184/full

39. Hassel S, Almeida JR, Kerr N, Nau S, Ladouceur CD, Fissell K, et al. Elevated striatal and decreased dorsolateral prefrontal cortical activity in response to emotional stimuli in euthymic bipolar disorder: no associations with psychotropic medication load. Bipolar Disorders. 2008;10(8):916–27.

40. Lagopoulos J, Malhi G. Impairments in “top-down” processing in bipolar disorder: A simultaneous fMRI–GSR study. Psychiatry Research: Neuroimaging. 2011 May 31;192(2):100–8.

41. Liu CH, Li F, Li SF, Wang YJ, Tie CL, Wu HY, et al. Abnormal baseline brain activity in bipolar depression: A resting state functional magnetic resonance imaging study. Psychiatry Research: Neuroimaging. 2012 Aug 1;203(2):175–9.

42. Lui S, Yao L, Xiao Y, Keedy SK, Reilly JL, Keefe RS, et al. Resting-state brain function in schizophrenia and psychotic bipolar probands and their first-degree relatives. Psychol Med. 2015 Jan;45(1):97–108.

43. Mah L, Zarate CA, Singh J, Duan YF, Luckenbaugh DA, Manji HK, et al. Regional Cerebral Glucose Metabolic Abnormalities in Bipolar II Depression. Biological Psychiatry. 2007 Mar 15;61(6):765–75.

44. Malhi GS, Lagopoulos J, Sachdev PS, Ivanovski B, Shnier R. An emotional Stroop functional MRI study of euthymic bipolar disorder. Bipolar Disorders. 2005;7(s5):58–69.

45. Malhi GS, Lagopoulos J, Owen AM, Ivanovski B, Shnier R, Sachdev P. Reduced activation to implicit affect induction in euthymic bipolar patients: An fMRI study. Journal of Affective Disorders. 2007 Jan 1;97(1):109–22.

46. Man V, Gruber J, Glahn DC, Cunningham WA. Altered amygdala circuits underlying valence processing among manic and depressed phases in bipolar adults. Journal of Affective Disorders. 2019 Feb 15;245:394–402.

47. Manelis A, Almeida JRC, Stiffler R, Lockovich JC, Aslam HA, Phillips ML. Anticipation-related brain connectivity in bipolar and unipolar depression: a graph theory approach. Brain. 2016 Sep 1;139(9):2554–66.

48. Marchand WR, Lee JN, Thatcher GW, Jensen C, Stewart D, Dilda V, et al. A functional MRI study of a paced motor activation task to evaluate frontal-subcortical circuit function in bipolar depression. Psychiatry Research: Neuroimaging. 2007 Aug 15;155(3):221–30.

49. McIntosh AM, Whalley HC, McKirdy J, Hall J, Sussmann JED, Shankar P, et al. Prefrontal Function and Activation in Bipolar Disorder and Schizophrenia. AJP. 2008 Mar 1;165(3):378–84.

50. Negoias S, Chen B, Iannilli E, Ning Y, Kitzler HH, Hummel T, et al. Odor-related brain hyper-reactivity in euthymic bipolar disorder: An fMRI and ERP study. Psychiatry Research. 2019 Aug 1;278:218–27.

51. Oertel-Knöchel V, Reinke B, Feddern R, Knake A, Knöchel C, Prvulovic D, et al. Verbal episodic memory deficits in remitted bipolar patients: A combined behavioural and fMRI study. Journal of Affective Disorders. 2013 Sep 5;150(2):430–40.

52. Pomarol-Clotet E, Alonso-Lana S, Moro N, Sarró S, Bonnin MC, Goikolea JM, et al. Brain functional changes across the different phases of bipolar disorder. The British Journal of Psychiatry. 2015 Feb;206(2):136–44.

53. Pompei F, Dima D, Rubia K, Kumari V, Frangou S. Dissociable functional connectivity changes during the Stroop task relating to risk, resilience and disease expression in bipolar disorder. NeuroImage. 2011 Jul 15;57(2):576–82.

54. Redlich R, Dohm K, Grotegerd D, Opel N, Zwitserlood P, Heindel W, et al. Reward Processing in Unipolar and Bipolar Depression: A Functional MRI Study. Neuropsychopharmacology. 2015 Oct;40(11):2623–31.

55. Shaffer JJ, Johnson CP, Fiedorowicz JG, Christensen GE, Wemmie JA, Magnotta VA. Impaired sensory processing measured by functional MRI in Bipolar disorder manic and depressed mood states. Brain Imaging and Behavior. 2018 Jun 1;12(3):837–47.

56. Sharma A, Satterthwaite TD, Vandekar L, Katchmar N, Daldal A, Ruparel K, et al. Divergent relationship of depression severity to social reward responses among patients with bipolar versus unipolar depression. Psychiatry Research: Neuroimaging. 2016 Aug 30;254:18–25.

57. Tecelão D, Mendes A, Martins D, Fu C, Chaddock CA, Picchioni MM, et al. The effect of psychosis associated CACNA1C, and its epistasis with ZNF804A, on brain function. Genes, Brain and Behavior. 2019;18(4):e12510.

58. Townsend JD, Torrisi SJ, Lieberman MD, Sugar CA, Bookheimer SY, Altshuler LL. Frontal-Amygdala Connectivity Alterations During Emotion Downregulation in Bipolar I Disorder. Biological Psychiatry. 2013 Jan 15;73(2):127–35.

59. Xu K, Liu H, Li H, Tang Y, Womer F, Jiang X, et al. Amplitude of low-frequency fluctuations in bipolar disorder: A resting state fMRI study. Journal of Affective Disorders. 2014 Jan 1;152–154:237–42.

60. Zhang L, vander Meer L, Opmeer EM, Marsman JBC, Ruhé HG, Aleman A. Altered functional connectivity during self- and close other-reflection in patients with bipolar disorder with past psychosis and patients with schizophrenia. Neuropsychologia. 2016 Dec 1;93:97–105.

61. Chepenik LG, Raffo M, Hampson M, Lacadie C, Wang F, Jones MM, et al. Functional connectivity between ventral prefrontal cortex and amygdala at low frequency in the resting state in bipolar disorder. Psychiatry Research: Neuroimaging. 2010 Jun 30;182(3):207–10.

62. Foland-Ross LC, Bookheimer SY, Lieberman MD, Sugar CA, Townsend JD, Fischer J, et al. Normal amygdala activation but deficient ventrolateral prefrontal activation in adults with bipolar disorder during euthymia. NeuroImage. 2012 Jan 2;59(1):738–44.

63. Rey G, Desseilles M, Favre S, Dayer A, Piguet C, Aubry JM, et al. Modulation of brain response to emotional conflict as a function of current mood in bipolar disorder: Preliminary findings from a follow-up state-based fMRI study. Psychiatry Research: Neuroimaging. 2014 Aug 30;223(2):84–93.

64. Zhang J, Magioncalda P, Huang Z, Tan Z, Hu X, Hu Z, et al. Altered Global Signal Topography and Its Different Regional Localization in Motor Cortex and Hippocampus in Mania and Depression. Schizophr Bull. 2019 Jun 18;45(4):902–10.

65. Brady Jr. RO, Tandon N, Masters GA, Margolis A, Cohen BM, Keshavan M, et al. Differential brain network activity across mood states in bipolar disorder. Journal of Affective Disorders. 2017 Jan 1;207:367–76.

66. Gong J, Chen G, Jia Y, Zhong S, Zhao L, Luo X, et al. Disrupted functional connectivity within the default mode network and salience network in unmedicated bipolar II disorder. Progress in Neuro-Psychopharmacology and Biological Psychiatry. 2019 Jan 10;88:11–8.

67. Goya‐Maldonado R, Brodmann K, Keil M, Trost S, Dechent P, Gruber O. Differentiating unipolar and bipolar depression by alterations in large-scale brain networks. Human Brain Mapping. 2016;37(2):808–18.

68. Liang MJ, Zhou Q, Yang KR, Yang XL, Fang J, Chen WL, et al. Identify Changes of Brain Regional Homogeneity in Bipolar Disorder and Unipolar Depression Using Resting-State fMRI. PLOS ONE. 2013 Dec 4;8(12):e79999.

69. Liu CH, Ma X, Wu X, Zhang Y, Zhou FC, Li F, et al. Regional homogeneity of resting-state brain abnormalities in bipolar and unipolar depression. Progress in Neuro-Psychopharmacology and Biological Psychiatry. 2013 Mar 5;41:52–9.

70. Wang Y, Zhong S, Jia Y, Sun Y, Wang B, Liu T, et al. Disrupted Resting-State Functional Connectivity in Nonmedicated Bipolar Disorder. Radiology. 2016 Feb 24;280(2):529–36.

71. Wang Y, Wang J, Jia Y, Zhong S, Niu M, Sun Y, et al. Shared and Specific Intrinsic Functional Connectivity Patterns in Unmedicated Bipolar Disorder and Major Depressive Disorder. Scientific Reports. 2017 Jun 15;7(1):3570.

72. Wang J, Wang Y, Wu X, Huang H, Jia Y, Zhong S, et al. Shared and specific functional connectivity alterations in unmedicated bipolar and major depressive disorders based on the triple-network model. Brain Imaging and Behavior. 2020 Feb 1;14(1):186–99.

73. Yang Y, Liu S, Jiang X, Yu H, Ding S, Lu Y, et al. Common and Specific Functional Activity Features in Schizophrenia, Major Depressive Disorder, and Bipolar Disorder. Front Psychiatry [Internet]. 2019 [cited 2020 Dec 6];10. Available from: https://www.frontiersin.org/articles/10.3389/fpsyt.2019.00052/full

74. Zhong S, Chen G, Zhao L, Jia Y, Chen F, Qi Z, et al. Correlation between Intrinsic Brain Activity and Thyroid-Stimulating Hormone Level in Unmedicated Bipolar II Depression. NEN. 2019;108(3):232–43.

75. Anticevic A, Yang G, Savic A, Murray JD, Cole MW, Repovs G, et al. Mediodorsal and Visual Thalamic Connectivity Differ in Schizophrenia and Bipolar Disorder With and Without Psychosis History. Schizophr Bull. 2014 Nov 1;40(6):1227–43.

76. Qiu M, Zhang H, Mellor D, Shi J, Wu C, Huang Y, et al. Aberrant Neural Activity in Patients With Bipolar Depressive Disorder Distinguishing to the Unipolar Depressive Disorder: A Resting-State Functional Magnetic Resonance Imaging Study. Front Psychiatry [Internet]. 2018 [cited 2020 Dec 6];9. Available from: https://www.frontiersin.org/articles/10.3389/fpsyt.2018.00238/full

77. Alonso-Lana S, Valentí M, Romaguera A, Sarri C, Sarró S, Rodríguez-Martínez A, et al. Brain functional changes in first-degree relatives of patients with bipolar disorder: evidence for default mode network dysfunction. Psychol Med. 2016;46(12):2513–21.

78. Benson BE, Willis MW, Ketter TA, Speer A, Kimbrell TA, Herscovitch P, et al. Differential abnormalities of functional connectivity of the amygdala and hippocampus in unipolar and bipolar affective disorders. Journal of Affective Disorders. 2014 Oct 15;168:243–53.

79. Goikolea JM, Dima D, Landín-Romero R, Torres I, DelVecchio G, Valentí M, et al. Multimodal Brain Changes in First-Episode Mania: A Voxel-Based Morphometry, Functional Magnetic Resonance Imaging, and Connectivity Study. Schizophr Bull. 2019 Mar 7;45(2):464–73.

80. Liberg B, Adler M, Jonsson T, Landén M, Rahm C, Wahlund LO, et al. The neural correlates of self-paced finger tapping in bipolar depression with motor retardation. Acta Neuropsychiatrica. 2013 Feb;25(1):43–51.

81. Robinson JL, Bearden CE, Monkul ES, Tordesillas‐Gutiérrez D, Velligan DI, Frangou S, et al. Fronto-temporal dysregulation in remitted bipolar patients: an fMRI delayed-non-match-to-sample (DNMS) study. Bipolar Disorders. 2009;11(4):351–60.

82. Rodríguez-Cano E, Alonso-Lana S, Sarró S, Fernández-Corcuera P, Goikolea JM, Vieta E, et al. Differential failure to deactivate the default mode network in unipolar and bipolar depression. Bipolar Disord. 2017;19(5):386–95.

83. Townsend JD, Sugar CA, Walshaw PD, Vasquez RE, Foland-Ross LC, Moody TD, et al. Frontostriatal neuroimaging findings differ in patients with bipolar disorder who have or do not have ADHD comorbidity. Journal of Affective Disorders. 2013 May 1;147(1):389–96.

84. Benson BE, Willis MW, Ketter TA, Speer A, Kimbrell TA, George MS, et al. Interregional cerebral metabolic associativity during a continuous performance task (Part II) : Differential alterations in bipolar and unipolar disorders. Psychiatry Research: Neuroimaging. 2008 Oct 30;164(1):30–47.

85. Brown A, Biederman J, Valera E, Lomedico A, Aleardi M, Makris N, et al. Working memory network alterations and associated symptoms in adults with ADHD and Bipolar Disorder. Journal of Psychiatric Research. 2012 Apr 1;46(4):476–83.

86. Jamadar S, O’Neil KM, Pearlson GD, Ansari M, Gill A, Jagannathan K, et al. Impairment in Semantic Retrieval is Associated with Symptoms in Schizophrenia but not Bipolar Disorder. Biological Psychiatry. 2013 Mar 15;73(6):555–64.

87. Marchand WR, Lee JN, Garn C, Thatcher J, Gale P, Kreitschitz S, et al. Striatal and cortical midline activation and connectivity associated with suicidal ideation and depression in bipolar II disorder. Journal of Affective Disorders. 2011 Oct 1;133(3):638–45.

88. Pompei F, Jogia J, Tatarelli R, Girardi P, Rubia K, Kumari V, et al. Familial and disease specific abnormalities in the neural correlates of the Stroop Task in Bipolar Disorder. NeuroImage. 2011 Jun 1;56(3):1677–84.

89. Reavis EA, Lee J, Wynn JK, Engel SA, Cohen MS, Nuechterlein KH, et al. Assessing neural tuning for object perception in schizophrenia and bipolar disorder with multivariate pattern analysis of fMRI data. NeuroImage: Clinical. 2017 Jan 1;16:491–7.

90. Taylor Tavares JV, Clark L, Furey ML, Williams GB, Sahakian BJ, Drevets WC. Neural basis of abnormal response to negative feedback in unmedicated mood disorders. NeuroImage. 2008 Sep 1;42(3):1118–26.

91. Brooks JO, Vizueta N, Penfold C, Townsend JD, Bookheimer SY, Altshuler LL. Prefrontal hypoactivation during working memory in bipolar II depression. Psychological Medicine. 2015 Jun;45(8):1731–40.

92. Drapier D, Surguladze S, Marshall N, Schulze K, Fern A, Hall MH, et al. Genetic Liability for Bipolar Disorder Is Characterized by Excess Frontal Activation in Response to a Working Memory Task. Biological Psychiatry. 2008 Sep 15;64(6):513–20.

93. Glahn DC, Robinson JL, Tordesillas‐Gutierrez D, Monkul ES, Holmes MK, Green MJ, et al. Fronto-temporal dysregulation in asymptomatic bipolar I patients: A paired associate functional MRI study. Human Brain Mapping. 2010;31(7):1041–51.

94. Liberg B, Adler M, Jonsson T, Landén M, Rahm C, Wahlund LO, et al. Motor Imagery in Bipolar Depression With Slowed Movement. The Journal of Nervous and Mental Disease. 2013 Oct;201(10):885–93.

95. Monks PJ, Thompson JM, Bullmore ET, Suckling J, Brammer MJ, Williams SC, et al. A functional MRI study of working memory task in euthymic bipolar disorder: evidence for task-specific dysfunction. Bipolar Disorders. 2004;6(6):550–64.

96. Fleck DE, Kotwal R, Eliassen JC, Lamy M, Delbello MP, Adler CM, et al. Preliminary evidence for increased frontosubcortical activation on a motor impulsivity task in mixed episode bipolar disorder. Journal of Affective Disorders. 2011 Sep 1;133(1):333–9.

97. Joshi SH, Vizueta N, Foland-Ross L, Townsend JD, Bookheimer SY, Thompson PM, et al. Relationships Between Altered Functional Magnetic Resonance Imaging Activation and Cortical Thickness in Patients With Euthymic Bipolar I Disorder. Biological Psychiatry: Cognitive Neuroscience and Neuroimaging. 2016 Nov 1;1(6):507–17.

98. Palaniyappan L, Liddle PF. Diagnostic Discontinuity in Psychosis: A Combined Study of Cortical Gyrification and Functional Connectivity. Schizophr Bull. 2014 May 1;40(3):675–84.

99. Sugihara G, Kane F, Picchioni MM, Chaddock CA, Kravariti E, Kalidindi S, et al. Effects of risk for bipolar disorder on brain function: A twin and family study. European Neuropsychopharmacology. 2017 May 1;27(5):494–503.

100. Yoshimura Y, Okamoto Y, Onoda K, Okada G, Toki S, Yoshino A, et al. Psychosocial functioning is correlated with activation in the anterior cingulate cortex and left lateral prefrontal cortex during a verbal fluency task in euthymic bipolar disorder: a preliminary fMRI study. Psychiatry Clin Neurosci. 2014 Mar;68(3):188–96.

101. Fernández-Corcuera P, Salvador R, Monté GC, Salvador Sarró S, Goikolea JM, Amann B, et al. Bipolar depressed patients show both failure to activate and failure to de-activate during performance of a working memory task. Journal of Affective Disorders. 2013 Jun 1;148(2):170–8.

102. Alonso-Lana S, Goikolea JM, Bonnin CM, Sarró S, Segura B, Amann BL, et al. Structural and Functional Brain Correlates of Cognitive Impairment in Euthymic Patients with Bipolar Disorder. PLOS ONE. 2016 Jul 22;11(7):e0158867.

103. Alonso‐Lana S, Moro N, McKenna PJ, Sarró S, Romaguera A, Monté GC, et al. Longitudinal brain functional changes between mania and euthymia in bipolar disorder. Bipolar Disorders. 2019;21(5):449–57.

104. Pomarol-Clotet E, Moro N, Sarró S, Goikolea JM, Vieta E, Amann B, et al. Failure of de-activation in the medial frontal cortex in mania: evidence for default mode network dysfunction in the disorder. The World Journal of Biological Psychiatry. 2012 Dec 1;13(8):616–26.

105. Chen CH, Lennox B, Jacob R, Calder A, Lupson V, Bisbrown-Chippendale R, et al. Explicit and Implicit Facial Affect Recognition in Manic and Depressed States of Bipolar Disorder: A Functional Magnetic Resonance Imaging Study. Biological Psychiatry. 2006 Jan 1;59(1):31–9.

106. Foland LC, Altshuler LL, Bookheimer SY, Eisenberger N, Townsend J, Thompson PM. Evidence for deficient modulation of amygdala response by prefrontal cortex in bipolar mania. Psychiatry Research: Neuroimaging. 2008 Jan 15;162(1):27–37.

107. Korgaonkar MS, Erlinger M, Breukelaar IA, Boyce P, Hazell P, Antees C, et al. Amygdala Activation and Connectivity to Emotional Processing Distinguishes Asymptomatic Patients With Bipolar Disorders and Unipolar Depression. Biological Psychiatry: Cognitive Neuroscience and Neuroimaging. 2019 Apr 1;4(4):361–70.

108. Lagopoulos J, Malhi GS. A functional magnetic resonance imaging study of emotional Stroop in euthymic bipolar disorder. NeuroReport. 2007 Oct 8;18(15):1583–7.

109. Malhi GS, Lagopoulos J, Sachdev PS, Ivanovski B, Shnier R, Ketter T. Is a lack of disgust something to fear? A functional magnetic resonance imaging facial emotion recognition study in euthymic bipolar disorder patients. Bipolar Disord. 2007 Jun;9(4):345–57.

110. Mitchell RLC, Elliott R, Barry M, Cruttenden A, Woodruff PWR. Neural response to emotional prosody in schizophrenia and in bipolar affective disorder. The British Journal of Psychiatry. 2004 Mar;184(3):223–30.

111. Perlman SB, Almeida JR, Kronhaus DM, Versace A, LaBarbara EJ, Klein CR, et al. Amygdala activity and prefrontal cortex–amygdala effective connectivity to emerging emotional faces distinguish remitted and depressed mood states in bipolar disorder. Bipolar Disorders. 2012;14(2):162–74.

112. Vai B, Poletti S, Radaelli D, Dallaspezia S, Bulgarelli C, Locatelli C, et al. Successful antidepressant chronotherapeutics enhance fronto-limbic neural responses and connectivity in bipolar depression. Psychiatry Research: Neuroimaging. 2015 Aug 30;233(2):243–53.

113. Vizueta N, Rudie JD, Townsend JD, Torrisi S, Moody TD, Bookheimer SY, et al. Regional fMRI Hypoactivation and Altered Functional Connectivity During Emotion Processing in Nonmedicated Depressed Patients With Bipolar II Disorder. AJP. 2012 Aug 1;169(8):831–40.

114. Wang F, Kalmar JH, He Y, Jackowski M, Chepenik LG, Edmiston EE, et al. Functional and Structural Connectivity Between the Perigenual Anterior Cingulate and Amygdala in Bipolar Disorder. Biological Psychiatry. 2009 Sep 1;66(5):516–21.

115. Yip SW, Worhunsky PD, Rogers RD, Goodwin GM. Hypoactivation of the Ventral and Dorsal Striatum During Reward and Loss Anticipation in Antipsychotic and Mood Stabilizer-Naive Bipolar Disorder. Neuropsychopharmacology. 2015 Feb;40(3):658–66.

116. Young KD, Bodurka J, Drevets WC. Differential neural correlates of autobiographical memory recall in bipolar and unipolar depression. Bipolar Disord. 2016;18(7):571–82.

117. Lennox BR, Jacob R, Calder AJ, Lupson V, Bullmore ET. Behavioural and neurocognitive responses to sad facial affect are attenuated in patients with mania. Psychological Medicine. 2004 Jul;34(5):795–802.

118. Morris RW, Sparks A, Mitchell PB, Weickert CS, Green MJ. Lack of cortico-limbic coupling in bipolar disorder and schizophrenia during emotion regulation. Translational Psychiatry. 2012 Mar;2(3):e90–e90.

119. Favre P, Baciu M, Pichat C, De Pourtalès MA, Fredembach B, Garçon S, et al. Modulation of fronto-limbic activity by the psychoeducation in euthymic bipolar patients. A functional MRI study. Psychiatry Research: Neuroimaging. 2013 Dec 30;214(3):285–95.

120. Jogia J, Haldane M, Cobb A, Kumari V, Frangou S. Pilot investigation of the changes in cortical activation during facial affect recognition with lamotrigine monotherapy in bipolar disorder. The British Journal of Psychiatry. 2008 Mar;192(3):197–201.

121. Killgore WDS, Gruber SA, Yurgelun-Todd DA. Abnormal Cortico-Striatal Activity During Fear Perception in Bipolar Disorder. Neuroreport. 2008 Oct 8;19(15):1523–7.

122. Trost S, Diekhof EK, Zvonik K, Lewandowski M, Usher J, Keil M, et al. Disturbed Anterior Prefrontal Control of the Mesolimbic Reward System and Increased Impulsivity in Bipolar Disorder. Neuropsychopharmacol. 2014 Jul;39(8):1914–23.

123. Chase HW, Nusslock R, Almeida JR, Forbes EE, LaBarbara EJ, Phillips ML. Dissociable patterns of abnormal frontal cortical activation during anticipation of an uncertain reward or loss in bipolar versus major depression. Bipolar Disorders. 2013;15(8):839–54.

124. Willert A, Mohnke S, Erk S, Schnell K, Romanczuk‐Seiferth N, Quinlivan E, et al. Alterations in neural Theory of Mind processing in euthymic patients with bipolar disorder and unaffected relatives. Bipolar Disorders. 2015;17(8):880–91.

125. Hassel S, Almeida JR, Kerr N, Nau S, Ladouceur CD, Fissell K, et al. Elevated striatal and decreased dorsolateral prefrontal cortical activity in response to emotional stimuli in euthymic bipolar disorder: no associations with psychotropic medication load. Bipolar Disorders. 2008;10(8):916–27.

126. Almeida JRC, Versace A, Hassel S, Kupfer DJ, Phillips ML. Elevated Amygdala Activity to Sad Facial Expressions: A State Marker of Bipolar but Not Unipolar Depression. Biological Psychiatry. 2010 Mar 1;67(5):414–21.

127. Mechelli A, Prata DP, Fu CHY, Picchioni M, Kane F, Kalidindi S, et al. The effects of neuregulin1 on brain function in controls and patients with schizophrenia and bipolar disorder. NeuroImage. 2008 Aug 15;42(2):817–26.

128. Trost S, Diekhof EK, Zvonik K, Lewandowski M, Usher J, Keil M, et al. Disturbed Anterior Prefrontal Control of the Mesolimbic Reward System and Increased Impulsivity in Bipolar Disorder. Neuropsychopharmacology. 2014 Jul;39(8):1914–23.

129. Blumberg HP, Donegan NH, Sanislow CA, Collins S, Lacadie C, Skudlarski P, et al. Preliminary evidence for medication effects on functional abnormalities in the amygdala and anterior cingulate in bipolar disorder. Psychopharmacology. 2005 Dec 1;183(3):308–13.

130. Dima D, de Jong S, Breen G, Frangou S. The polygenic risk for bipolar disorder influences brain regional function relating to visual and default state processing of emotional information. NeuroImage: Clinical. 2016 Feb 1;12:838–44.

131. Dima D, Roberts RE, Frangou S. Connectomic markers of disease expression, genetic risk and resilience in bipolar disorder. Translational Psychiatry. 2016 Jan;6(1):e706–e706.

132. Elliott R, Ogilvie A, Rubinsztein JS, Calderon G, Dolan RJ, Sahakian BJ. Abnormal ventral frontal response during performance of an affective go/no go task in patients with mania. Biological Psychiatry. 2004 Jun 15;55(12):1163–70.

133. Jogia J, Dima D, Kumari V, Frangou S. Frontopolar cortical inefficiency may underpin reward and working memory dysfunction in bipolar disorder. The World Journal of Biological Psychiatry. 2012 Dec 1;13(8):605–15.

134. Mason L, O’Sullivan N, Montaldi D, Bentall RP, El-Deredy W. Decision-making and trait impulsivity in bipolar disorder are associated with reduced prefrontal regulation of striatal reward valuation. Brain. 2014 Aug 1;137(8):2346–55.

135. Poletti S, de Wit H, Mazza E, Wijkhuijs AJM, Locatelli C, Aggio V, et al. Th17 cells correlate positively to the structural and functional integrity of the brain in bipolar depression and healthy controls. Brain, Behavior, and Immunity. 2017 Mar 1;61:317–25.

136. Sepede G, De Berardis D, Campanella D, Perrucci MG, Ferretti A, Salerno RM, et al. Neural correlates of negative emotion processing in bipolar disorder. Progress in Neuro-Psychopharmacology and Biological Psychiatry. 2015 Jul 3;60:1–10.

137. Allin MPG, Marshall N, Schulze K, Walshe M, Hall MH, Picchioni M, et al. A functional MRI study of verbal fluency in adults with bipolar disorder and their unaffected relatives. Psychol Med. 2010 Dec;40(12):2025–35.

138. Almeida JRC, Mechelli A, Hassel S, Versace A, Kupfer DJ, Phillips ML. Abnormally increased effective connectivity between parahippocampal gyrus and ventromedial prefrontal regions during emotion labeling in bipolar disorder. Psychiatry Research: Neuroimaging. 2009 Dec 30;174(3):195–201.

139. Ambrosi E, Arciniegas DB, Madan A, Curtis KN, Patriquin MA, Jorge RE, et al. Insula and amygdala resting-state functional connectivity differentiate bipolar from unipolar depression. Acta Psychiatrica Scandinavica. 2017;136(1):129–39.

140. Avery SN, Williams LE, Woolard AA, Heckers S. Relational memory and hippocampal function in psychotic bipolar disorder. Eur Arch Psychiatry Clin Neurosci. 2014 Apr 1;264(3):199–211.

141. Bermpohl F, Kahnt T, Dalanay U, Hägele C, Sajonz B, Wegner T, et al. Altered representation of expected value in the orbitofrontal cortex in mania. Human Brain Mapping. 2010;31(7):958–69.

142. Berns GS, Martin M, Proper SM. Limbic Hyperreactivity in Bipolar II Disorder. AJP. 2002 Feb 1;159(2):304–6.

143. Blumberg HP, Stern E, Ricketts S, Martinez D, de Asis J, White T, et al. Rostral and Orbital Prefrontal Cortex Dysfunction in the Manic State of Bipolar Disorder. AJP. 1999 Dec 1;156(12):1986–8.

144. Blumberg HP, Leung HC, Skudlarski P, Lacadie CM, Fredericks CA, Harris BC, et al. A functional magnetic resonance imaging study of bipolar disorder: state- and trait-related dysfunction in ventral prefrontal cortices. Arch Gen Psychiatry. 2003 Jun;60(6):601–9.

145. Bøen E, Hjørnevik T, Hummelen B, Elvsåshagen T, Moberget T, Holtedahl JE, et al. Patterns of altered regional brain glucose metabolism in borderline personality disorder and bipolar II disorder. Acta Psychiatrica Scandinavica. 2019;139(3):256–68.

146. Brady RO, Masters GA, Mathew IT, Margolis A, Cohen BM, Öngür D, et al. State dependent cortico-amygdala circuit dysfunction in bipolar disorder. Journal of Affective Disorders. 2016 Sep 1;201:79–87.

147. Brandt CL, Eichele T, Melle I, Sundet K, Server A, Agartz I, et al. Working memory networks and activation patterns in schizophrenia and bipolar disorder: comparison with healthy controls. The British Journal of Psychiatry. 2014 Apr;204(4):290–8.

148. Brooks JO, Hoblyn JC, Woodard SA, Rosen AC, Ketter TA. Corticolimbic metabolic dysregulation in euthymic older adults with bipolar disorder. Journal of Psychiatric Research. 2009 Feb 1;43(5):497–502.

149. Brooks JO, Rosen AC, Hoblyn JC, Woodard SA, Krasnykh O, Ketter TA. Resting Prefrontal Hypometabolism and Paralimbic Hypermetabolism Related to Verbal Recall Deficits in Euthymic Older Adults With Bipolar Disorder. The American Journal of Geriatric Psychiatry. 2009 Dec 1;17(12):1022–9.

150. Brooks JO, Wang PW, Bonner JC, Rosen AC, Hoblyn JC, Hill SJ, et al. Decreased prefrontal, anterior cingulate, insula, and ventral striatal metabolism in medication-free depressed outpatients with bipolar disorder. Journal of Psychiatric Research. 2009 Jan 1;43(3):181–8.

151. Brooks JO, Bearden CE, Hoblyn JC, Woodard SA, Ketter TA. Prefrontal and paralimbic metabolic dysregulation related to sustained attention in euthymic older adults with bipolar disorder. Bipolar Disorders. 2010;12(8):866–74.

152. Brooks JO, Hoblyn JC, Ketter TA. Metabolic evidence of corticolimbic dysregulation in bipolar mania. Psychiatry Research: Neuroimaging. 2010 Feb 28;181(2):136–40.

153. Bürger C, Redlich R, Grotegerd D, Meinert S, Dohm K, Schneider I, et al. Differential Abnormal Pattern of Anterior Cingulate Gyrus Activation in Unipolar and Bipolar Depression: an fMRI and Pattern Classification Approach. Neuropsychopharmacology. 2017 Jun;42(7):1399–408.

154. Cantisani A, Stegmayer K, Bracht T, Federspiel A, Wiest R, Horn H, et al. Distinct resting-state perfusion patterns underlie psychomotor retardation in unipolar vs. bipolar depression. Acta Psychiatrica Scandinavica. 2016;134(4):329–38.

155. Caseras X, Lawrence NS, Murphy K, Wise RG, Phillips ML. Ventral Striatum Activity in Response to Reward: Differences Between Bipolar I and II Disorders. AJP. 2013 May 1;170(5):533–41.

156. Cerullo MA, Fleck DE, Eliassen JC, Smith MS, DelBello MP, Adler CM, et al. A longitudinal functional connectivity analysis of the amygdala in bipolar I disorder across mood states. Bipolar Disorders. 2012 Mar 1;14(2):175–84.

157. Chai XJ, Whitfield-Gabrieli S, Shinn AK, Gabrieli JDE, Nieto Castañón A, McCarthy JM, et al. Abnormal Medial Prefrontal Cortex Resting-State Connectivity in Bipolar Disorder and Schizophrenia. Neuropsychopharmacology. 2011 Sep;36(10):2009–17.

158. Chakirova G, Whalley HC, Thomson PA, Hennah W, Moorhead TWJ, Welch KA, et al. The effects of DISC1 risk variants on brain activation in controls, patients with bipolar disorder and patients with schizophrenia. Psychiatry Research: Neuroimaging. 2011 Apr 30;192(1):20–8.

159. Chen G, Zhao L, Jia Y, Zhong S, Chen F, Luo X, et al. Abnormal cerebellum-DMN regions connectivity in unmedicated bipolar II disorder. Journal of Affective Disorders. 2019 Jan 15;243:441–7.

160. Curtis VA, Thompson JM, Seal ML, Monks PJ, Lloyd AJ, Harrison L, et al. The nature of abnormal language processing in euthymic bipolar I disorder: evidence for a relationship between task demand and prefrontal function. Bipolar Disorders. 2007;9(4):358–69.

161. Deckersbach T, Rauch SL, Buhlmann U, Ostacher MJ, Beucke JC, Nierenberg AA, et al. An fMRI investigation of working memory and sadness in females with bipolar disorder: a brief report. Bipolar Disorders. 2008;10(8):928–42.

162. Dell’Osso B, Cinnante C, Di Giorgio A, Cremaschi L, Palazzo MC, Cristoffanini M, et al. Altered prefrontal cortex activity during working memory task in Bipolar Disorder: A functional Magnetic Resonance Imaging study in euthymic bipolar I and II patients. Journal of Affective Disorders. 2015 Sep 15;184:116–22.

163. Delvecchio G, Dima D, Frangou S. The effect of ANK3 bipolar-risk polymorphisms on the working memory circuitry differs between loci and according to risk-status for bipolar disorder. American Journal of Medical Genetics Part B: Neuropsychiatric Genetics. 2015;168(3):188–96.

164. Frangou S. Risk and resilience in bipolar disorder: rationale and design of the Vulnerability to Bipolar Disorders Study (VIBES). Biochemical Society Transactions. 2009 Sep 21;37(5):1085–9.

165. Deng W, Zhang B, Zou W, Zhang X, Cheng X, Guan L, et al. Abnormal Degree Centrality Associated With Cognitive Dysfunctions in Early Bipolar Disorder. Front Psychiatry [Internet]. 2019 [cited 2020 Dec 6];10. Available from: https://www.frontiersin.org/articles/10.3389/fpsyt.2019.00140/full

166. Fateh AA, Long Z, Duan X, Cui Q, Pang Y, Farooq MU, et al. Hippocampal functional connectivity-based discrimination between bipolar and major depressive disorders. Psychiatry Research: Neuroimaging. 2019 Feb 28;284:53–60.

167. Favre P, Baciu M, Pichat C, Bougerol T, Polosan M. fMRI evidence for abnormal resting-state functional connectivity in euthymic bipolar patients. Journal of Affective Disorders. 2014 Aug 20;165:182–9.

168. Favre P, Polosan M, Pichat C, Bougerol T, Baciu M. Cerebral Correlates of Abnormal Emotion Conflict Processing in Euthymic Bipolar Patients: A Functional MRI Study. PLOS ONE. 2015 Aug 5;10(8):e0134961.

169. Fournier JC, Keener MT, Almeida J, Kronhaus DM, Phillips ML. Amygdala and whole-brain activity to emotional faces distinguishes major depressive disorder and bipolar disorder. Bipolar Disorders. 2013;15(7):741–52.

170. Fournier JC, Chase HW, Almeida J, Phillips ML. Within- and Between-Session Changes in Neural Activity During Emotion Processing in Unipolar and Bipolar Depression. Biological Psychiatry: Cognitive Neuroscience and Neuroimaging. 2016 Nov 1;1(6):518–27.

171. Frangou S, Kington J, Raymont V, Shergill SS. Examining ventral and dorsal prefrontal function in bipolar disorder: A functional magnetic resonance imaging study. European Psychiatry. 2008 Jun 1;23(4):300–8.

172. Frangou S, Dima D, Jogia J. Towards person-centered neuroimaging markers for resilience and vulnerability in Bipolar Disorder. NeuroImage. 2017 Jan 15;145:230–7.

173. Grant K, Hassel S, Bobyn JA, Hall GBC, MacQueen GM. A novel task for examining the neural basis of Theory of Mind deficits in bipolar disorder. Psychiatry Research: Neuroimaging. 2018 Dec 30;282:143–50.

174. Hall J, Whalley HC, Marwick K, McKirdy J, Sussmann J, Romaniuk L, et al. Hippocampal function in schizophrenia and bipolar disorder. 2010 May [cited 2020 Dec 6]; Available from: https://era.ed.ac.uk/handle/1842/3470

175. Hamilton LS, Altshuler LL, Townsend J, Bookheimer SY, Phillips OR, Fischer J, et al. Alterations in functional activation in euthymic bipolar disorder and schizophrenia during a working memory task. Human Brain Mapping. 2009;30(12):3958–69.

176. Han X, Liu X, Li L, Xie B, Fan B, Qiu Y, et al. Neural Activation During Tonic Pain and Interaction Between Pain and Emotion in Bipolar Disorder: An fMRI Study. Front Psychiatry [Internet]. 2018 [cited 2020 Dec 6];9. Available from: https://www.frontiersin.org/articles/10.3389/fpsyt.2018.00555/full

177. Han S, He Z, Duan X, Tang Q, Chen Y, Yang Y, et al. Dysfunctional connectivity between raphe nucleus and subcortical regions presented opposite differences in bipolar disorder and major depressive disorder. Progress in Neuro-Psychopharmacology and Biological Psychiatry. 2019 Jun 8;92:76–82.

178. He Z, Sheng W, Lu F, Long Z, Han S, Pang Y, et al. Altered resting-state cerebral blood flow and functional connectivity of striatum in bipolar disorder and major depressive disorder. Progress in Neuro-Psychopharmacology and Biological Psychiatry. 2019 Mar 2;90:177–85.

179. Horacek J, Mikolas P, Tintera J, Novak T, Palenicek T, Brunovsky M, et al. Sad mood induction has an opposite effect on amygdala response to emotional stimuli in euthymic patients with bipolar disorder and healthy controls. J Psychiatry Neurosci. 2015 Mar;40(2):134–42.

180. Hulvershorn LA, Karne H, Gunn AD, Hartwick SL, Wang Y, Hummer TA, et al. Neural Activation During Facial Emotion Processing in Unmedicated Bipolar Depression, Euthymia, and Mania. Biological Psychiatry. 2012 Apr 1;71(7):603–10.

181. Hummer TA, Hulvershorn LA, Karne HS, Gunn AD, Wang Y, Anand A. Emotional Response Inhibition in Bipolar Disorder: A Functional Magnetic Resonance Imaging Study of Trait- and State-Related Abnormalities. Biological Psychiatry. 2013 Jan 15;73(2):136–43.

182. Ives-Deliperi VL, Howells F, Stein DJ, Meintjes EM, Horn N. The effects of mindfulness-based cognitive therapy in patients with bipolar disorder: A controlled functional MRI investigation. Journal of Affective Disorders. 2013 Sep 25;150(3):1152–7.

183. Jogia J, Dima D, Frangou S. Sex differences in bipolar disorder: a review of neuroimaging findings and new evidence. Bipolar Disorders. 2012;14(4):461–71.

184. Kaladjian A, Jeanningros R, Azorin JM, Nazarian B, Roth M, Anton JL, et al. Remission from mania is associated with a decrease in amygdala activation during motor response inhibition. Bipolar Disorders. 2009;11(5):530–8.

185. Keener MT, Fournier JC, Mullin BC, Kronhaus D, Perlman SB, LaBarbara E, et al. Dissociable patterns of medial prefrontal and amygdala activity to face identity versus emotion in bipolar disorder. Psychol Med. 2012 Sep;42(9):1913–24.

186. Kim E, Jung YC, Ku J, Kim JJ, Lee H, Kim SY, et al. Reduced activation in the mirror neuron system during a virtual social cognition task in euthymic bipolar disorder. Progress in Neuro-Psychopharmacology and Biological Psychiatry. 2009 Nov 13;33(8):1409–16.

187. Kirschner M, Cathomas F, Manoliu A, Habermeyer B, Simon JJ, Seifritz E, et al. Shared and dissociable features of apathy and reward system dysfunction in bipolar I disorder and schizophrenia. Psychological Medicine. 2020 Apr;50(6):936–47.

188. Lagopoulos J, Ivanovski B, Malhi GS. An event-related functional MRI study of working memory in euthymic bipolar disorder. J Psychiatry Neurosci. 2007 May;32(3):174–84.

189. Lee J, Reavis EA, Engel SA, Altshuler LL, Cohen MS, Glahn DC, et al. fMRI evidence of aberrant neural adaptation for objects in schizophrenia and bipolar disorder. Hum Brain Mapp. 2019 01;40(5):1608–17.

190. Li G, Liu P, Andari E, Zhang A, Zhang K. The Role of Amygdala in Patients With Euthymic Bipolar Disorder During Resting State. Front Psychiatry [Internet]. 2018 [cited 2020 Dec 6];9. Available from: https://www.frontiersin.org/articles/10.3389/fpsyt.2018.00445/full

191. Liu C, Ma X, Zhen Y, Zhang Y, Tang L, Feng L, et al. The effect of lithium on resting-state brain networks in patients with bipolar depression. Journal of Translational Neuroscience. 2016 Oct 14;1(1):43–51.

192. Luo X, Chen G, Jia Y, Gong J, Qiu S, Zhong S, et al. Disrupted Cerebellar Connectivity With the Central Executive Network and the Default-Mode Network in Unmedicated Bipolar II Disorder. Front Psychiatry [Internet]. 2018 [cited 2020 Dec 6];9. Available from: https://www.frontiersin.org/articles/10.3389/fpsyt.2018.00705/full

193. Magioncalda P, Martino M, Conio B, Escelsior A, Piaggio N, Presta A, et al. Functional connectivity and neuronal variability of resting state activity in bipolar disorder—reduction and decoupling in anterior cortical midline structures. Human Brain Mapping. 2015;36(2):666–82.

194. Maïza O, Razafimandimby A, Brazo P, Lecardeur L, Delamillieure P, Mazoyer B, et al. Functional deficit in the medial prefrontal cortex in patients with chronic schizophrenia, first psychotic episode, and bipolar disorders. Bipolar Disord. 2010 Jun;12(4):450–2.

195. Malhi GS, Lagopoulos J, Das P, Moss K, Berk M, Coulston CM. A functional MRI study of Theory of Mind in euthymic bipolar disorder patients. Bipolar Disord. 2008 Dec;10(8):943–56.

196. Marchand WR, Lee JN, Garn C, Thatcher J, Gale P, Kreitschitz S, et al. Aberrant emotional processing in posterior cortical midline structures in bipolar II depression. Progress in Neuro-Psychopharmacology and Biological Psychiatry. 2011 Aug 15;35(7):1729–37.

197. Marotta G, Delvecchio G, Pigoni A, Mandolini G, Ciappolino V, Oldani L, et al. The metabolic basis of psychosis in bipolar disorder: A positron emission tomography study. Bipolar Disorders. 2019;21(2):151–8.

198. Martino M, Magioncalda P, Saiote C, Conio B, Escelsior A, Rocchi G, et al. Abnormal functional–structural cingulum connectivity in mania: combined functional magnetic resonance imaging-diffusion tensor imaging investigation in different phases of bipolar disorder. Acta Psychiatrica Scandinavica. 2016;134(4):339–49.

199. Mason L, O’Sullivan N, Montaldi D, Bentall RP, El-Deredy W. Decision-making and trait impulsivity in bipolar disorder are associated with reduced prefrontal regulation of striatal reward valuation. Brain. 2014 Aug 1;137(8):2346–55.

200. McKenna BS, Sutherland AN, Legenkaya AP, Eyler LT. Abnormalities of brain response during encoding into verbal working memory among euthymic patients with bipolar disorder. Bipolar Disorders. 2014;16(3):289–99.

201. Mechelli A, Fusar-Poli P, Prata D, Papagni SA, Tognin S, Kambeitz J, et al. Genetic vulnerability to psychosis and cortical function: epistatic effects between DAAO and G72. Curr Pharm Des. 2012;18(4):510–7.

202. Mullin BC, Perlman SB, Versace A, de Almeida JRC, LaBarbara EJ, Klein C, et al. An fMRI study of attentional control in the context of emotional distracters in euthymic adults with bipolar disorder. Psychiatry Research: Neuroimaging. 2012;201(3):196–205.

203. Nusslock R, Almeida JR, Forbes EE, Versace A, Frank E, LaBarbara EJ, et al. Waiting to win: elevated striatal and orbitofrontal cortical activity during reward anticipation in euthymic bipolar disorder adults. Bipolar Disorders. 2012;14(3):249–60.

204. Oertel‐Knöchel V, Reinke B, Feddern R, Knake A, Knöchel C, Prvulovic D, et al. Episodic memory impairments in bipolar disorder are associated with functional and structural brain changes. Bipolar Disorders. 2014;16(8):830–45.

205. Oertel-Knöchel V, Reinke B, Matura S, Prvulovic D, Linden DEJ, Ven V van de. Functional connectivity pattern during rest within the episodic memory network in association with episodic memory performance in bipolar disorder. Psychiatry Research: Neuroimaging. 2015 Feb 28;231(2):141–50.

206. Palaniyappan L, Mota NB, Oowise S, Balain V, Copelli M, Ribeiro S, et al. Speech structure links the neural and socio-behavioural correlates of psychotic disorders. Progress in Neuro-Psychopharmacology and Biological Psychiatry. 2019 Jan 10;88:112–20.

207. Penfold C, Vizueta N, Townsend JD, Bookheimer SY, Altshuler LL. Frontal lobe hypoactivation in medication-free adults with bipolar II depression during response inhibition. Psychiatry Research: Neuroimaging. 2015 Mar 30;231(3):202–9.

208. Qiu S, Chen F, Chen G, Jia Y, Gong J, Luo X, et al. Abnormal resting-state regional homogeneity in unmedicated bipolar II disorder. Journal of Affective Disorders. 2019 Sep 1;256:604–10.

209. Reinke B, Ven VV de, Matura S, Linden DEJ, Oertel-Knöchel V. Altered Intrinsic Functional Connectivity in Language-Related Brain Regions in Association with Verbal Memory Performance in Euthymic Bipolar Patients. Brain Sciences. 2013 Sep;3(3):1357–73.

210. Rive MM, Mocking RJT, Koeter MWJ, van Wingen G, de Wit SJ, van den Heuvel OA, et al. State-Dependent Differences in Emotion Regulation Between Unmedicated Bipolar Disorder and Major Depressive Disorder. JAMA Psychiatry. 2015 Jul;72(7):687–96.

211. Rive MM, Koeter MWJ, Veltman DJ, Schene AH, Ruhé HG. Visuospatial planning in unmedicated major depressive disorder and bipolar disorder: distinct and common neural correlates. Psychological Medicine. 2016 Aug;46(11):2313–28.

212. Rosenfeld ES, Pearlson GD, Sweeney JA, Tamminga CA, Keshavan MS, Nonterah C, et al. Prolonged hemodynamic response during incidental facial emotion processing in inter-episode bipolar I disorder. Brain Imaging and Behavior. 2014 Mar 1;8(1):73–86.

213. Roth RM, Koven NS, Randolph JJ, Flashman LA, Pixley HS, Ricketts SM, et al. Functional magnetic resonance imaging of executive control in bipolar disorder. NeuroReport. 2006 Jul 31;17(11):1085–9.

214. Ryan KA, Dawson EL, Kassel MT, Weldon AL, Marshall DF, Meyers KK, et al. Shared dimensions of performance and activation dysfunction in cognitive control in females with mood disorders. Brain. 2015 May 1;138(5):1424–34.

215. Skåtun KC, Kaufmann T, Tønnesen S, Biele G, Melle I, Agartz I, et al. Global brain connectivity alterations in patients with schizophrenia and bipolar spectrum disorders. J Psychiatry Neurosci. 2016 Sep;41(5):331–41.

216. Strakowski SM, Adler CM, Holland SK, Mills NP, DelBello MP, Eliassen JC. Abnormal fMRI Brain Activation in Euthymic Bipolar Disorder Patients During a Counting Stroop Interference Task. AJP. 2005 Sep 1;162(9):1697–705.

217. Strakowski SM, Eliassen JC, Lamy M, Cerullo MA, Allendorfer JB, Madore M, et al. Functional Magnetic Resonance Imaging Brain Activation in Bipolar Mania: Evidence for Disruption of the Ventrolateral Prefrontal-Amygdala Emotional Pathway. Biological Psychiatry. 2011 Feb 15;69(4):381–8.

218. Surguladze SA, Marshall N, Schulze K, Hall MH, Walshe M, Bramon E, et al. Exaggerated neural response to emotional faces in patients with bipolar disorder and their first-degree relatives. NeuroImage. 2010 Oct 15;53(1):58–64.

219. Syan SK, Minuzzi L, Smith M, Costescu D, Allega OR, Hall GBC, et al. Brain Structure and Function in Women with Comorbid Bipolar and Premenstrual Dysphoric Disorder. Front Psychiatry [Internet]. 2018 [cited 2020 Dec 6];8. Available from: https://www.frontiersin.org/articles/10.3389/fpsyt.2017.00301/full

220. Tecelão D, Mendes A, Martins D, Bramon E, Toulopoulou T, Kravariti E, et al. The impact of psychosis genome-wide associated ZNF804A variation on verbal fluency connectivity. Journal of Psychiatric Research. 2018 Mar 1;98:17–21.

221. Tesli M, Kauppi K, Bettella F, Brandt CL, Kaufmann T, Espeseth T, et al. Altered Brain Activation during Emotional Face Processing in Relation to Both Diagnosis and Polygenic Risk of Bipolar Disorder. PLOS ONE. 2015 Jul 29;10(7):e0134202.

222. Torrisi S, Moody TD, Vizueta N, Thomason ME, Monti MM, Townsend JD, et al. Differences in resting corticolimbic functional connectivity in bipolar I euthymia. Bipolar Disord. 2013 Mar;15(2):156–66.

223. Tseng WL, Thomas LA, Harkins E, Stoddard J, Zarate CA, Pine DS, et al. Functional connectivity during masked and unmasked face emotion processing in bipolar disorder. Psychiatry Research: Neuroimaging. 2016 Dec 30;258:1–9.

224. Usnich T, Spengler S, Sajonz B, Herold D, Bauer M, Bermpohl F. Perception of social stimuli in mania: An fMRI study. Psychiatry Research: Neuroimaging. 2015 Jan 30;231(1):71–6.

225. Vargas C, Pineda J, Calvo V, López-Jaramillo C. Brain activitivation of euthymic patients with Type I bipolar disorder in resting state Default Mode Network. Revista Colombiana de Psiquiatría. 2014 Jul;43(3):154–61.

226. Wang Y, Zhong S, Jia Y, Zhou Z, Zhou Q, Huang L. Reduced interhemispheric resting-state functional connectivity in unmedicated bipolar II disorder. Acta Psychiatrica Scandinavica. 2015;132(5):400–7.

227. Wang Y, Zhong S, Chen G, Liu T, Zhao L, Sun Y, et al. Altered cerebellar functional connectivity in remitted bipolar disorder: A resting-state functional magnetic resonance imaging study. Aust N Z J Psychiatry. 2018 Oct 1;52(10):962–71.

228. Wang X, Zhao N, Shi J, Wu Y, Liu J, Xiao Q, et al. Discussion on Patients with Bipolar Disorder and Depressive Episode by Ratio Low Frequency Amplitude Combined with Grey Matter Volume Analysis. J Med Syst. 2019 Mar 23;43(5):117.

229. Wessa M, Houenou J, Paillère-Martinot ML, Berthoz S, Artiges E, Leboyer M, et al. Fronto-Striatal Overactivation in Euthymic Bipolar Patients During an Emotional Go/NoGo Task. AJP. 2007 Apr 1;164(4):638–46.

230. Whittaker JR, Foley SF, Ackling E, Murphy K, Caseras X. The Functional Connectivity Between the Nucleus Accumbens and the Ventromedial Prefrontal Cortex as an Endophenotype for Bipolar Disorder. Biol Psychiatry. 2018 Dec 1;84(11):803–9.

231. Yin Z, Chang M, Wei S, Jiang X, Zhou Y, Cui L, et al. Decreased Functional Connectivity in Insular Subregions in Depressive Episodes of Bipolar Disorder and Major Depressive Disorder. Front Neurosci [Internet]. 2018 [cited 2020 Dec 6];12. Available from: https://www.frontiersin.org/articles/10.3389/fnins.2018.00842/full

232. Yip SW, Mackay CE, Goodwin GM. Increased temporo-insular engagement in unmedicated bipolar II disorder: an exploratory resting state study using independent component analysis. Bipolar Disord. 2014 Nov;16(7):748–55.

233. Yu HL, Liu WB, Wang T, Huang PY, Jie LY, Sun JZ, et al. Difference in resting-state fractional amplitude of low-frequency fluctuation between bipolar depression and unipolar depression patients. Eur Rev Med Pharmacol Sci. 2017;21(7):1541–50.

234. Zhang K, Liu Z, Cao X, Yang C, Xu Y, Xu T, et al. Amplitude of low-frequency fluctuations in first-episode, drug-naïve depressive patients: A 5-year retrospective study. PLoS One [Internet]. 2017 Apr 6 [cited 2020 Dec 6];12(4). Available from: https://www.ncbi.nlm.nih.gov/pmc/articles/PMC5383053/

235. Zhang L, Ai H, Opmeer EM, Marsman JBC, Meer L van der, Ruhé HG, et al. Distinct temporal brain dynamics in bipolar disorder and schizophrenia during emotion regulation. Psychological Medicine. 2020 Feb;50(3):413–21.

236. Zhao L, Wang Y, Jia Y, Zhong S, Sun Y, Qi Z, et al. Altered interhemispheric functional connectivity in remitted bipolar disorder: A Resting State fMRI Study. Scientific Reports. 2017 Jul 5;7(1):4698.

237. Zhou Q, Womer FY, Kong L, Wu F, Jiang X, Zhou Y, et al. Trait-Related Cortical-Subcortical Dissociation in Bipolar Disorder: Analysis of Network Degree Centrality. J Clin Psychiatry. 2017 May 24;78(5):584–91.

1. In ALE terminology, a “paper” or “study” refers to a published item whereas an “experiment” refers to a set of coordinates corresponding to a specific contrast analysis. [↑](#footnote-ref-1)
2. One study(23) in which 7/8 participants were unmedicated was coded as unmedicated. [↑](#footnote-ref-2)
3. Mean ages and standard deviations for euthymic and depressed samples were averaged. [↑](#footnote-ref-3)
4. Mean ages and standard deviations for depressed and hypo/manic samples were averaged. [↑](#footnote-ref-4)
5. Mean ages and standard deviations for BD groups (with/without psychosis history) were averaged. [↑](#footnote-ref-5)
6. Mean ages and standard deviations for unmedicated and medicated groups were averaged. [↑](#footnote-ref-6)
7. Mean ages and standard deviations for hypo/manic and euthymic groups were averaged. [↑](#footnote-ref-7)
8. Authors report “all but three patients were medicated” but did not indicate from which patient group; this study was coded as greater than 75% medicated assuming the 3 unmedicated participants had BD. [↑](#footnote-ref-8)
9. Mean ages and standard deviations for BD I and II groups were averaged. [↑](#footnote-ref-9)
10. Mean ages and standard deviations for hypo/manic and depressed groups were averaged. [↑](#footnote-ref-10)
11. Authors reported “most” BD patients were medicated, thus this study was coded as greater than 75% medicated. [↑](#footnote-ref-11)
12. 7/8 BD participants were unmedicated, thus this study was coded as unmedicated. [↑](#footnote-ref-12)
13. Mean ages and standard deviations for depressed, euthymic, and hypo/manic groups were averaged. [↑](#footnote-ref-13)
14. Mean ages and standard deviations for depressed, euthymic, and hypo/manic groups were averaged. [↑](#footnote-ref-14)
15. Mean ages and standard deviations for BD groups with and without lithium treatment were averaged. [↑](#footnote-ref-15)
16. Mean ages and standard deviations for hypo/manic and depressed groups were averaged. [↑](#footnote-ref-16)
17. Mean ages and standard deviations for groups with and without psychosis history were averaged. [↑](#footnote-ref-17)
18. Mean ages and standard deviations of high-risk and low-risk groups were averaged. [↑](#footnote-ref-18)
19. Authors reported “most” BD patients were medicated, thus this study was coded as greater than 75% medicated. [↑](#footnote-ref-19)
20. Mean ages and standard deviations for hypo/manic, euthymic, and depressed groups were averaged. [↑](#footnote-ref-20)
21. Same cohort scanned during different mood states. [↑](#footnote-ref-21)
22. Mean ages and standard deviations for depressed and euthymic groups were averaged. [↑](#footnote-ref-22)
23. 2 participants missing from the analysis. [↑](#footnote-ref-23)
24. 2 participants missing from the analysis. [↑](#footnote-ref-24)
25. Mean ages and standard deviations for depressed and hypo/manic groups were averaged. [↑](#footnote-ref-25)
26. Medication data missing for 6 participants. [↑](#footnote-ref-26)
27. Mean ages and standard deviations for the three genetic groups were averaged. [↑](#footnote-ref-27)
28. Mean ages and standard deviations for BD and BD with comorbid PMDD were averaged. [↑](#footnote-ref-28)
29. PANSS-P score on day of scanning only available for 44.7% of the BD sample. [↑](#footnote-ref-29)
30. Mean ages and standard deviations for BD and BD/comorbid ADHD groups were averaged. [↑](#footnote-ref-30)
31. Mean ages and standard deviations for groups taking and not taking medication were averaged. [↑](#footnote-ref-31)
